# Efficient blockLASSO for polygenic scores with applications to All of Us and UK Biobank

**DOI:** 10.1186/s12864-025-11505-0

**Published:** 2025-03-27

**Authors:** Timothy G. Raben, Louis Lello, Erik Widen, Stephen D. H. Hsu

**Affiliations:** 1 Department of Physics and Astronomy, Michigan State University, East Lansing, USA; 2 Genomic Prediction, Inc., North Brunswick, NJ USA

## Abstract

We develop a “block” LASSO (blockLASSO) approach for training polygenic scores (PGS) and demonstrate its use in All of Us (AoU) and the UK Biobank (UKB). blockLASSO utilizes the approximate block diagonal structure (due to chromosomal partition of the genome) of linkage disequilibrium (LD). The new implementation can be used for exploratory and methods research where repeated PGS training is necessary and expensive. For 11 different phenotypes, in two different biobanks, and across 5 different ancestry groups (African, American, East Asian, European, and South Asian) – we demonstrate that blockLASSO is generally as effective for training PGS as a (global) LASSO. Previous work has shown penalized regression methods produce competitive PGS to alternative approaches. It has been shown that some phenotypes are more/less polygenic than others. Using sparse algorithms, an accurate PGS can be trained for type 1 diabetes (T1D) using ${\sim }100$ single nucleotide variants (SNVs), but a PGS for body mass index (BMI) would need more than 10k SNVs. blockLASSO produces similar PGS for phenotypes while training with just a fraction of the variants per block. Within AoU (using only genetic information) block PGS for T1D reaches an AUC of $0.63_{\pm 0.02}$ and for BMI a correlation of $0.21_{\pm 0.01}$, whereas a global LASSO approach which finds for T1D an AUC $0.65_{\pm 0.03}$ and BMI a correlation $0.19_{\pm 0.03}$. This new block approach is more computationally efficient and scalable than naive global machine learning approaches and makes it ideal for exploratory methods investigations based on penalized regression.

**Supplementary Information:**

The online version contains supplementary material available at 10.1186/s12864-025-11505-0.

## Introduction

Polygenic scores (PGS) are becoming important tools for understanding genetic architecture [1–3], identifying potential genetic risk of disease [4, 5], and now have clinical applications [6]. PGS are traditionally built in one of two approaches: (1) starting from single marker regression and adding in additional genomic information or (2) training algorithms executed directly on a subset of the genome. In this paper we present an approximate implementation to approach (2) that can be used in exploratory stages and methods development to very efficiently estimate the performance of polygenic scores without high computational costs.

The approach (1) principally relies on the results of single marker regression, or genome wide association studies (GWAS). The GWAS results are then re-weighted using linkage disequilibrium (LD) structure (i.e., the correlation structure of single nucleotide variants or SNVs), functional information, fine mapping, meta-analyses, or ancestry specific effects. This approach has the advantage that GWAS is computationally efficient (it can be run completely in parallel) and that parts of the additional information can be computed separately. Examples of this approach include PRS-cs [7, 8] and LDpred [9–11] which uses as inputs the results of a GWAS and the LD information, and then re-weights the SNPs using continuous shrinkage and sparse priors respectively. This LD information can be computed a single time for a population and used in the creation of many predictors. Because LD matrices are computed in blocks (e.g., chromosome-by-chromosome), these methods can already utilize the block diagonal structure. The disadvantage of this approach is that the GWAS and LD matrices represent approximations of the genome level data and that information can be lost in this process.

The second approach relies on applying machine learning algorithms – penalized frequentist/Bayesian regressions, neural networks , decision trees, etc. [12–16] – directly on genome level data. The advantage here is that the algorithms train directly on genomes and don’t have to approximate any structure. The disadvantage is that this requires loading large genetic matrices into computer memory. For example, loading a matrix of 64 bit floats that includes 50,000 SNPs for 500,000 people requires roughly 200 gigabytes (GB) of memory. Training a PGS model then requires holding several of these matrices in memory. While there is no universally best method, simple LASSO and penalized regression based methods have routinely been found among the top performing methods when testing in different ancestry groups [15, 17].

**Fig. 1:**
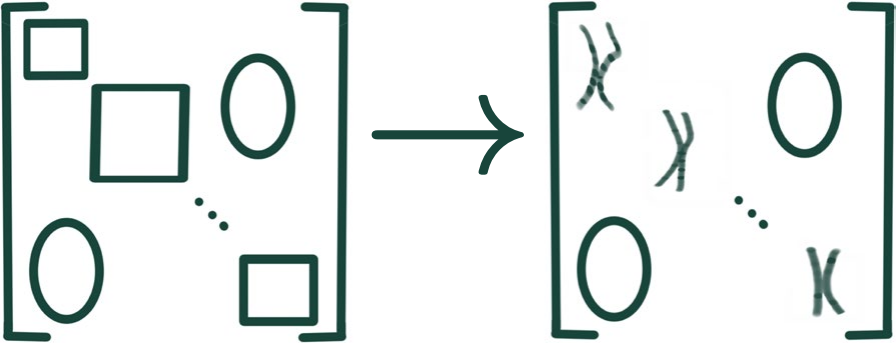
(Left) a generic block diagonal matrix. (Right) block diagonal matrices resulting from negligible correlations across (as opposed to within) chromosomes

Over the past decade there have been efforts to speed up LASSO computations by using “safe” [18] and “strong” [19] screening rules. Screening refers to identifying features that will remain with zero weight at successive LASSO hyper-parameter steps, thus reducing the effective dimensionality of the problem at that step. Several works have shown possible improved computational efficiency [20–22]. It was shown that the improvements from these screening rules depend on how they are implemented [23]. For example, some previous work that used the “safe” rules in a custom Julia implementation [24, 25] found similar results and similar computation times to a Python implementation using scikit-learn without the “safe” rules [17, 26, 27]. More recently these techniques have been applied to genomic analyses. First, Privé et al. [28] developed the “bigstatsr” R package which incorporated “strong” screening to do penalized regression, and then Qian et al. [29] implemented a *Batch Screening Iterative Lasso* (BASIL) using the “strong” rules. The implementation by Privé et al. implements “Strong” screening rules coupled with an “early stopping” procedure and it is demonstrated in the supplement of [15] that their approach is over an order of magnitude faster than BASIL. The “bigstatsr” approach uses “filebacked” big matrices to more efficiently handle large arrays without having to load them into memory. However, there is a trade-off in needing to pre-compute these matrices with a large amount of computing resources and hard disk space.

Here we utilize the underlying biological structure to greatly reduce computational time and resource requirements by computing an *approximate* LASSO solution. The main idea is that over large distances (e.g., from one chromosome to another) SNVs are uncorrelated. In other words, the correlation structure is block diagonal as in Fig. 1. From this assumption we can run LASSO (or another algorithm) on each block independently and then perform a re-weighting to find the relative importance of each block. While the *exact* LASSO solution is not recovered, the majority of the predictive power appears to remain and the computation times are 3–4 orders of magnitude faster than our traditional calculation and requires less than 5 times the memory of exact computations like in [15, 29]. Little attention has been paid toward approximations of the LASSO problem in its application to genetics, but there are some tangential projects that aim to approximate the LASSO [30, 31]. Additionally we choose to focus on developing this approach in Python as we feel that it is underdeveloped compared to other languages but readily available on third party cloud computing platforms offered by the UKB, AoU, and others. Because of the relative simplicity of our approach, it is straightforward to adapt to other languages.

## Results

**Fig. 2:**
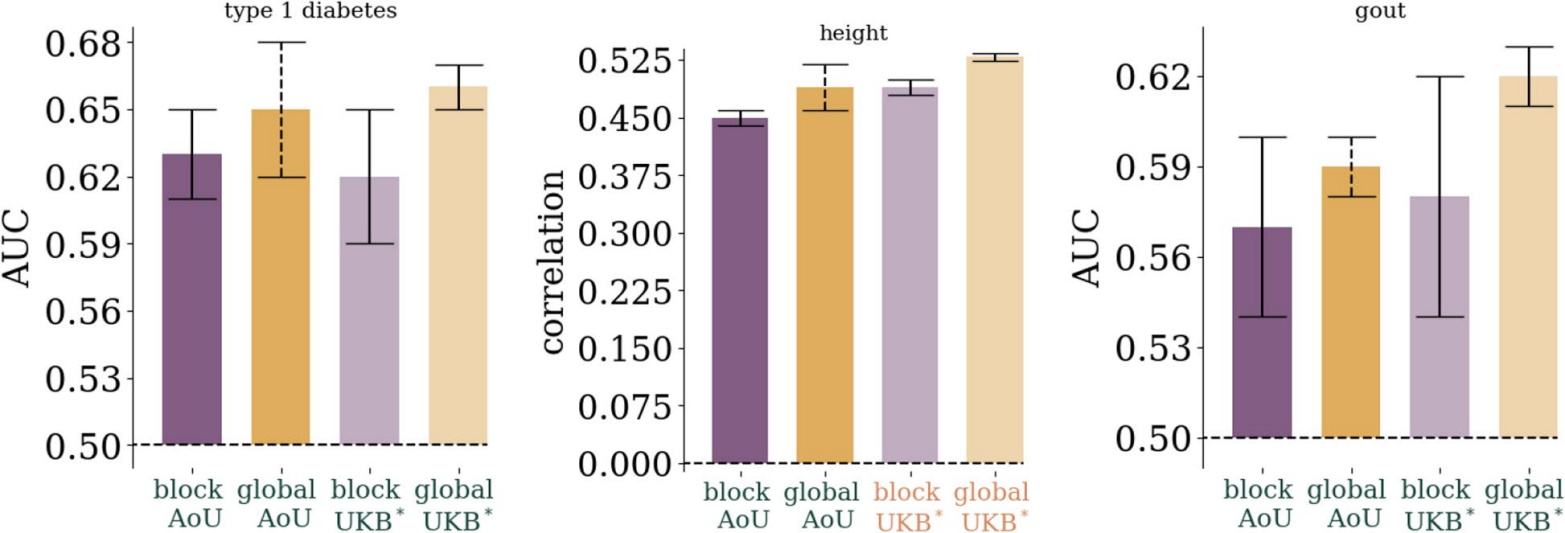
Comparison of block vs global LASSO in AoU and UKB$^*$ (UKB reduced to training/testing sets comparable to AoU). We see that for many conditions both the block and global results are in agreement in both biobanks *and* the results are in agreement between biobanks. Uncertainties reflect one standard deviation computed from 5-fold cross-validation and computing AUC/correlation with finite sample sizes. For the “global AoU” measurement only one training fold was run so the uncertainty is the larger of the finite size effect, or the corresponding uncertainty found in the UKB

**Fig. 3:**
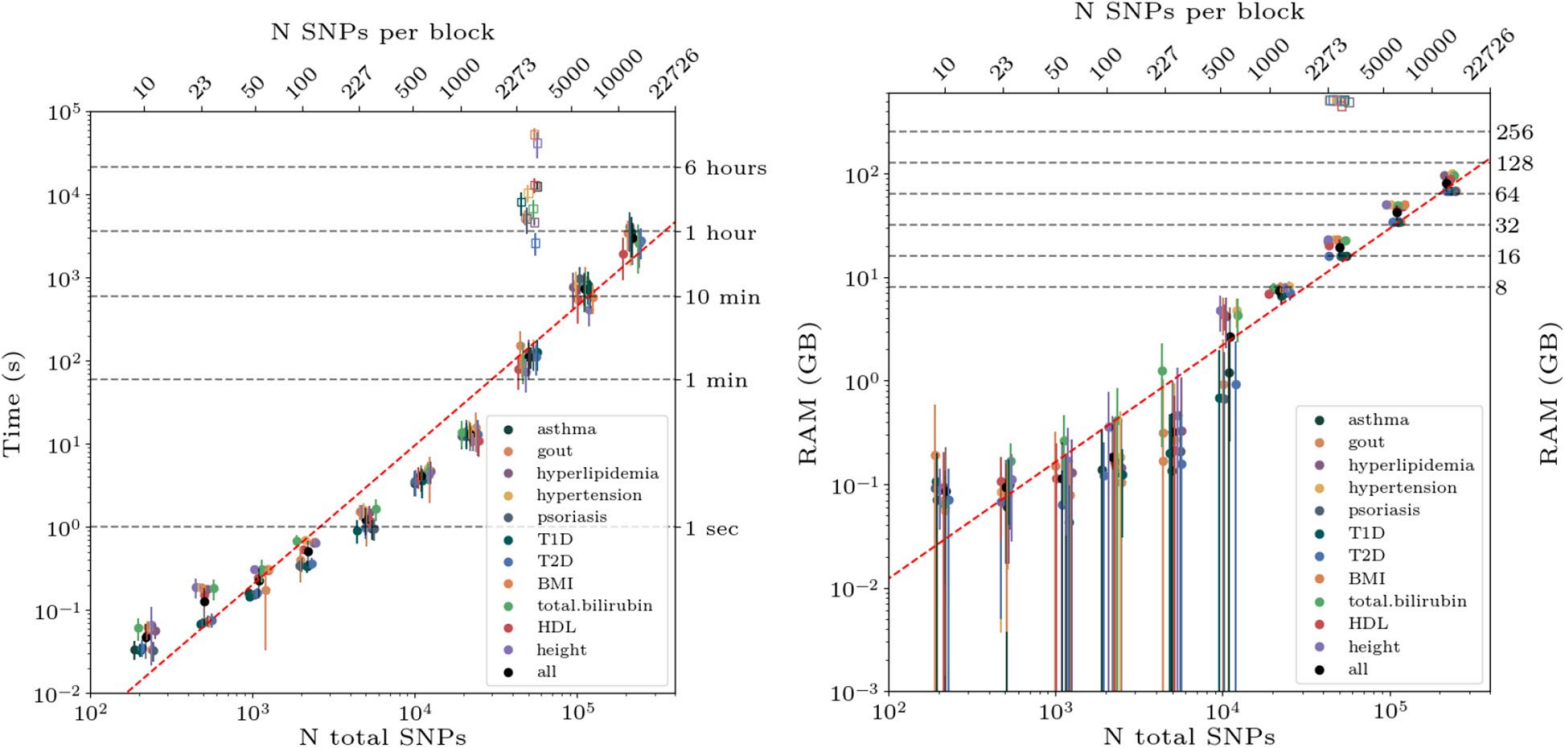
Computational details for the blockLASSO (filled in circles) in the UKB. Because the block algorithm runs each chromosome in parallel the results are averaged over both the chromosomes and cross-validation folds. The top axis indicates the number of features on each block (each chromosome in this case) and the bottom axis indicates the total number of features included in the algorithm. In this case the bottom axis is simple 23 times the top axis. Left: computation time (seconds) as a function of number of SNVs per chromosome. Error bars are standard deviation over runs. blockLASSO in the specific case of 2,273 SNVs per autosomal chromosome is compared to the Python based global LASSO (open squares) using 50,000 SNVs (or SNPs) and corresponds to an average ${\sim }133\times$ reduction in computation time. Right: RAM used per blockLASSO SNVs per chromosome. Again we compare the block approach with 2,273 SNVs per autosomal chromosome to the Python based global LASSO using 50,000 SNVs and find ${\sim }26\times$ less memory needed. For both plots error bars are standard deviations over runs. For most phenotypes and SNV sizes the error bars are very small

The main results can be seen in Table 1 and a subset are displayed in Fig. 2, while the full set of results are collected in the Supplementary Information. Results are presented in terms of Area Under the Receiver Operating Characteristic Curve (AUC) for case control phenotypes and phenotype to PGS correlation (corr.) for quantitative phenotypes. All uncertainties are standard deviations and standard errors of cross-validation folds and computing metrics with finite data sizes. *Global LASSO* refers to our standard Python based LASSO algorithm run with 50k features spread throughout the autosome [17]. *blockLASSO* refers to small LASSOs run chromosome by chromosome and then stitched together as described in “Methods” section. Table 1 shows the results for the blockLASSO vs a global LASSO for training in All of Us (AoU), the full UK Biobank (UKB), and the UKB with training/testing sets reduced to the sizes found in AoU (UKB$^*$), i.e., to give a more direct comparison. The global UKB$^*$ column is computed from the Monte Carlo bounds set by interpolating between training sizes [17]. Table cells highlighted in  indicate that the global LASSO and blockLASSO are not significantly different from each other, i.e., the error bars overlap between block and global approaches. We refer to this as *full* agreement. In AoU this is true for 4 case control conditions and 2 continuous phenotypes. The other agreement metric we use is marked in  . This indicates the overall signal loss from using the block strategy is less than $20\%$, e.g., $(\text {corr}_{\text {global}} - \text {corr}_{\text {block}})/\text {corr}_{\text {global}} \le 0.2$ and $(\text {AUC}_{\text {global}} - \text {AUC}_{\text {block}})/(\text {AUC}_{\text {global}}-0.5)\le 0.2$. We refer to this as *approximate* agreement. This is true for the other two continuous phenotypes in AoU. This color coding, to indicate the different types of agreement, is used in Table 1, Fig. 3, the Supplementary figures 19–21, and throughout the text.

For the reduced UK Biobank training (UKB$^*$) we see that 5 case-control and 3 continuous phenotypes are in full agreement between block and global LASSO. The remaining two phenotypes have a signal fall-off of less than $20\%$ and are in approximate agreement. Psoriasis was omitted as it is the one phenotype where there are more cases in AoU than in the UKB. In principle we could limit the number of cases on the AoU side, but this would be a different calculation than the others presented. In addition we see 9 different measurements are labeled in bold indicating that the results between AoU and UKB$^*$ have overlapping error bars. There are known phenotyping errors within AoU. For example, there are BMI measurements that are ${\sim }10^6\, \text {kg}/\text {m}^2$. Although these clearly unrealistic outlying measurements are filtered before training, there is still the possibility of residual phenotyping errors. Despite these possible phenotyping errors and using different SNV sets, the bolded results indicate that a similar level of polygenic prediction was found for 9 measurements between the two different biobanks.

Finally we see that for maximal training within the UKB 4 phenotypes have full agreement between block and global LASSO and 6 phenotypes have approximate agreement. Training with the full UKB uses much larger training and testing sets which is reflected in the smaller uncertainties.

**Table 1:**
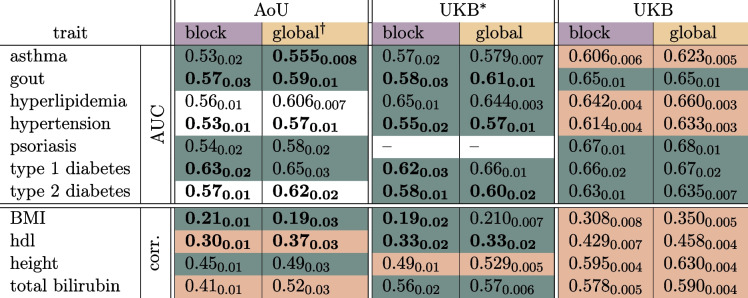
Summary of main PGS metrics for results in All of Us (AoU), the UK Biobank trained with sets matching the size of those found in AoU (UKB$^*$), and for the UK Biobank using the maximum possible training sizes (UKB). For one trait, psoriasis, there are actually more cases in AoU than in the UKB so the UKB$^*$ computation is left blank. All predictors are trained and tested on European populations. Blocks colored  indicate that the block and traditional results are not statistically different and the error bar values overlap. Blocks colored  indicate that the block and traditional results disagree by less than $20\%$. Finally, **bold text** indicates that the results between AoU and UKB (either block or traditional) are in agreement within uncertainty

**Fig. 4:**
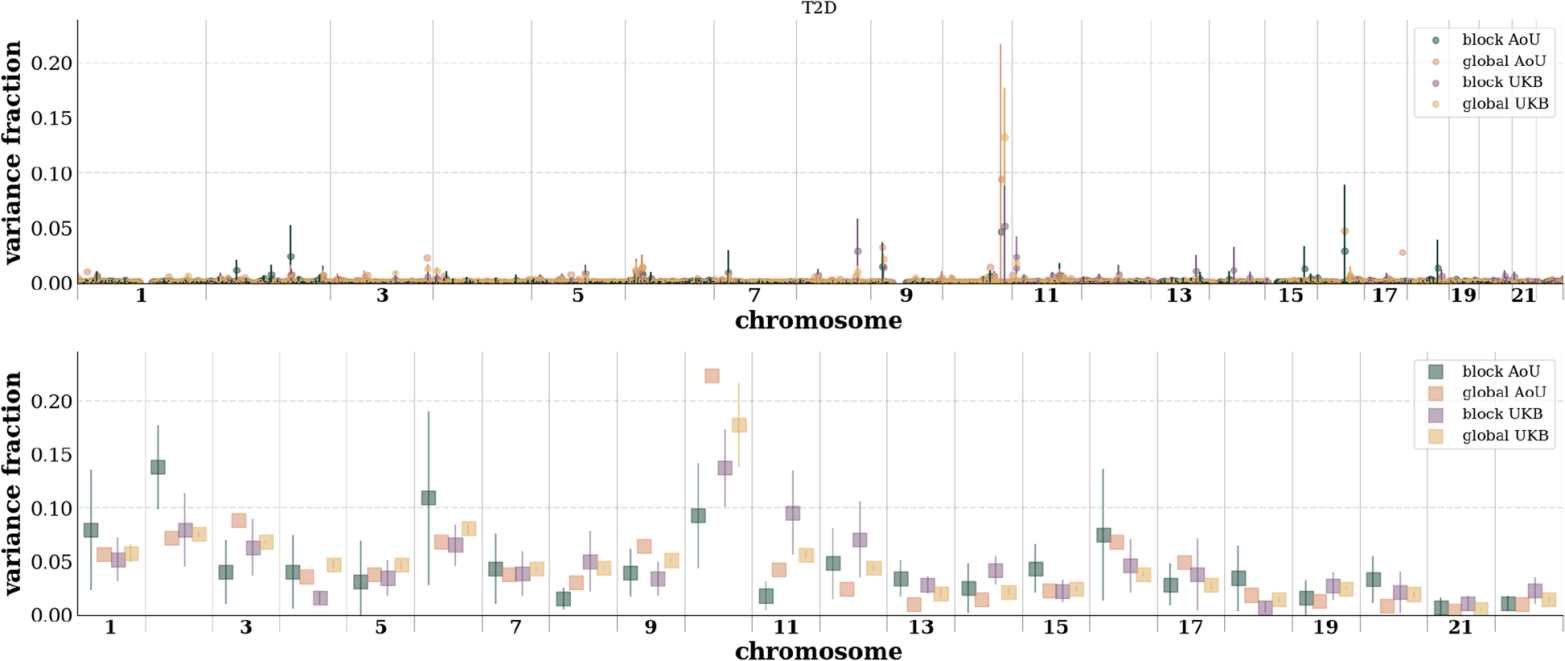
Comparison of block vs global LASSO binned variance per base pair position (top) and per chromosome (bottom) in AoU and the UKB for type 2 diabetes. This approximate variance is identified by computing the PGS covariance matrix and summing over rows as detailed in “PGS variance distributions” section. Error bars are standard deviations over cross-validation folds and then propagated in quadrature as nearby contributions are binned (top) or summed over the entire chromosome (bottom). For global LASSO in AoU only a single CV fold was computed. Similar plots for the other phenotypes can be found in Supplementary Information

Traditional global LASSO is limited by computation time, memory requirements, and cost, all three of which can limit the number of features (SNVs) and samples (individuals) used to train models. In previous research [17, 24, 25, 27, 32], a typical global LASSO was run on ${\sim }50$k SNPs and ${\sim }400$k people. These computations required between 500–700 GB of RAM and would complete after 8–24 hours of computational time. The computations were mostly done on 28 core Intel(R) Xeon(R) CPU E5-2680 v4 (2.40GHz) processors. Some older computations were done on 20 core Intel(R) Xeon(R) CPU E5-2670 v2 (2.50GHz) processors.

As seen in Fig. 3 the blockLASSO approach is much faster and uses much less memory than the naive implementation of the global LASSO. Using the full UKB we trained blockLASSO predictors using different numbers of SNPs per chromosome. Block results that obtain full agreement tend to happen for sparser predictors – gout, psoriasis, and type 1 diabetes – and this agreement is achieved with an average of 133 SNVs per chromosome, using 2.63 GB of RAM per chromosome, and running in 0.924 s. A single chromosome uses${\sim }190\times$ less RAM and runs in ${\sim }15,000\times$ less time compared to the scikit-learn based global LASSO with 50k SNVs. On average for *all phenotypes* we find that the block approach achieves the approximate agreement of $80\%$ of the global signal while training with 203 SNVs per chromosome, a single chromosome also runs with $\sim 190\times$ less memory and a single chromosome runs ${\sim }7,000\times$ faster than the scikit-learn based global LASSO with 50k SNVs. On the right hand side of Fig. 3 we see that the memory requirements increase at most linearly with the number of features. We can also compare the block and Python based global approach at the same total feature size, i.e., 2,273 SNVs per chromosome vs 50,000 SNVs across the autosome. Here the block approach uses ${\sim } 26\times$ less memory (largely a reflection of splitting the autosome into 22 blocks) and runs in ${\sim }133\times$ less times. Note that a similar order of magnitude increase in computational efficiency has been found by implementing “strong screening” (i.e., pre-feature selection) and an early stopping criterion [15]. While both of these might not be applicable for pure methods investigations, they can be combined with a blocked approach for even greater increased computational efficiency. The majority of the LASSO computational time is spent in the least sparse regions of the hyper-parameter space, i.e., where a larger number of features are given non-zero weights. An early stopping criterion can be helpful in avoiding this region, but as seen in the Supplementary Information it can be difficult to define an early stopping point with weaker signals or if testing in different ancestry groups.

Note that a similar order of magnitude increase in computational efficiency has been found by implementing “strong screening” (i.e., pre-feature selection) and an early stopping criterion [15]. While both of these might not be applicable for pure methods investigations, they can be combined with a blocked approach for even greater increased computational efficiency. The majority of the LASSO computational time is spent in the least sparse regions of the hyper-parameter space. An early stopping criterion can be helpful in avoiding this region, but as seen in the Supplementary Information it can be difficult to define an early stopping point with weaker signals or if testing in different ancestry groups.

The block and global approaches use different input SNVs. To see if they are capturing the same features we can look at the variance explained of the different PGSs. In Fig. 4 we see in the top panel the effective variance fraction explained by variants across the autosome for a type 2 diabetes PGS. Similar plots for the remaining phenotypes are in the Supplementary Information. As described in “PGS variance distributions” section, this approximate variance per location is computed by summing over one dimension of the feature correlation matrix. Error bars reflect the uncertainty over cross-validation folds, but only one fold is used for the global AoU results. Binning, and adding error bars in quadrature, of nearby positions is done to make the plot human readable. In the bottom panel, these contributions are summed to show the variance fraction explained per chromosome. Again, the error bars come from using multiple cross-validation folds. By comparing the blockLASSO to the traditional LASSO we see that the block approach recovers the same important regions with similar weights. Also analogously, by comparing the results from the UKB and AoU we see similar variance fractions explained in similar regions.

## Methods

### Phenotypes

The phenotypes used are a combination of self-report/survey data, ICD9/10 code diagnoses, and laboratory measurements. Exact combinations that make up each phenotype in both AoU and UKB can be found in the Supplementary Information. Additionally, we include the number of samples for each phenotype used in training. In general we do not use exclusion criteria, i.e. we have no criteria that would exclude a documented case or control (e.g., age of onset or minimum age). The only exception is for *psoriasis in AoU* where we found a less inclusive definition of cases and controls allowed for better results. While there are accurate PGS for psoriasis (e.g., [33–35]), it is also known that there are various types of psoriasis that can have separate genetic signatures [36–38]. For this phenotype alone, we use a set of exclusion criteria for both cases and controls.

Some pre-filtering is done on continuous phenotypes to exclude values that may reflect a rare genetic condition (e.g., dwarfism), possibly reflective of extreme lifestyle choices (extreme obesity), and to exclude obvious data errors. In AoU we use the following cuts on continuous phenotypes: BMI <60 kg/m$^{2}$ to filter implausible values, total bilirubin < 2.5mg/dl to filter unrealistic values, and hdl <200mg/dl to filter unrealistic values. In the UKB we require that measurements be positive values and that height >144cm.

After regressing phenotypes on covariates (age, sex, and principal components), we subtract off the covariate contribution from the raw phenotype. These residual distributions, plotted in the Supplementary Information, are used in the (block)LASSO training. Kolmogorov-Smirnov or Shapiro-Wilk tests show that these distributions are not Gaussian. However, this non-Gaussianity is displayed in several ways. First, for phenotypes where there is a strong covariate contribution, the residuals are multimodal. For most distributions the values are centrally peaked and have light-tails. In the Supplementary Information we show the residual distributions, including QQ-plots, and report the skewness and kurtosis. Even in the presence of non-Gaussian distributions, if the true genetic signals are sparse and linear then exact signal recovery is possible via $L_1$/compressed-sensing methods [39, 40]. Before the Donoho-Tanner phase boundary [41, 42] (where is exact signal recovery is possible) is crossed then LASSO can be sensitive to the features of the sample-feature distributions. This is especially true for extremely sparse signals [43] and heavy tailed distributions [44, 45]. However the phenotypes in this work were selected because $L_1$ methods have tended to perform similarly or better to other methods [15, 17].

### Populations

Within the UKB we largely relied on self-reported “Ethnic background” responses to identify ancestry. We used the following mapping from UKB fields: Asian or Asian British, Indian, Pakistani, Bangladeshi, Any other Asian background – South Asian (SAS); Black or Black British, Caribbean, African, Any other Black background – African (AFR); Chinese – East Asian (EAS); and White, British, Irish, Any other white background – European (EUR).

For both UKB and AoU we also computed genetic ancestry via ADMIXTURE [46]. This was used to identify a generalized American or Hispanic group in UKB and also general identification in AoU. We implemented Admixture on “1000 Genomes” [47] with 5 groupings – this generated an ancestry fraction for each individual for each of the 5 groups and we found that the ancestry fractions corresponded with their respective super-populations (EUR, AFR, AMR, SAS, EAS). These results were then applied to the UK Biobank where it was found that the ancestry fractions largely aligned with the 4 self-reported categories. Individuals with at least 0.35 ancestry fraction in the fifth grouping, which corresponded to AMR in 1000 Genomes, were labeled as American (AMR) and excluded from the other groupings. Within AoU we relied exclusively on the admixture approach but compared ancestry fractions against survey results for consistency. Again 5 groupings were used with ancestry fraction cuts of 0.7 for AFR, 0.3 for AMR, 0.8 for EAS, 0.9 for EUR, and 0.6 for SAS.

Within the UKB we identify genetic siblings to be used as a testing set (not used in training). Genetic siblings can be assumed to have more similar environmental backgrounds than random pairs. This leads to slightly reduced PGS accuracy as discussed in [26, 48]. The method for identifying siblings is described in [26] in Supplemental section C.

### Polygenic scores

This project demonstrates an efficient approach to building PGS that is applied block by block on the genome. To this end we are interested in *only the genetic component* of the PGS. Future work will be aimed at combining this approach with other effects (environmental, gene $\times$ environment interactions, etc.) to generate maximal possible prediction accuracy. To train these genetic-only predictors we take an approach to try to be as conservative as possible. The process can be briefly summarized as follows: (1) separate samples into training, model selection (validation), and testing sets; (2) regress phenotype on covariates and build residual phenotype; (3) identify candidate SNPs (4) perform block by block LASSO (5) re-weight relative blockLASSO results (6) evaluate on completely withheld testing sets.

(1) For all phenotypes we split the samples into training, model-selection/re-weighting, and testing sets. In both biobanks we used the EUR cohort for training as it had the largest sample sizes. We remove a small subset of the EUR group and the entire AFR, AMR, EAS, and SAS groups to be used for final testing sets. In the UKB the EUR testing set is a group of sibling pairs as described above. To avoid over-fitting, the testing sets in both biobanks, were completely withheld and only used to evaluate the final PGS performance. The testing sets were not used in any fitting, hyper-parameter selection, or parameter tuning. After removing the testing sets we assemble training and model-selection sets using 5-fold cross-validation. Exact set sizes are given in the Supplementary Information.

Next we build residual phenotypes as step (2) using the training sets. For a case-control phenotype we regress the raw phenotype on covariates – age, sex, and principal components (top 16 in AoU and top 20 in UKB). For continuous phenotypes we do sex-specific z-scoring so that we can combine the sex assorted data into a single, much larger training set. We then use linear regression on age and principal components. For all phenotypes we then subtract off the effect of these covariates to make a residual phenotype. Building residual phenotypes allow us to give *conservative* estimates of the genetic effects since we first assume that the covariates have a *maximal* impact and then we train the genetics on just the remaining information. Additionally, because PRS have not be extensively trained in AoU we did global LASSO training for case-control phenotypes on the raw phenotype allowing us to be conservative about our statement of agreement between block and global results.

Step (3) involves identifying candidate features (SNVs). For both global LASSO and blockLASSO approaches we perform a GWAS using the training set and rank SNVs by p-values across the entire autosome or block (chromosome) respectively. This GWAS is used purely to rank SNVs and *none* of the other GWAS information (e.g., p-values, odds ratios, weights) is retained or used. In previous research involving case-control phenotypes in the UKB it was found that rank ordering results from a GWAS on the *raw phenotype* (e.g., [25, 26]) lead to similar results as rank ordering results from a GWAS on *residual* phenotype (e.g., [17]). For the block approach, *within the UKB*, this remained true. However, when we moved to AoU, we found, only for the block approach, that selecting features based on a GWAS of the raw phenotype under-performed selecting features based on a GWAS of the residual phenotype. This is likely a result of the AoU biobank being more diverse both environmentally and ancestrally causing these covariates to have a much larger effect. When selecting the top features we used a cut off to include only SNVs with allele frequency $>0.001$ in the training population to avoid spurious associations.

**Fig. 5:**
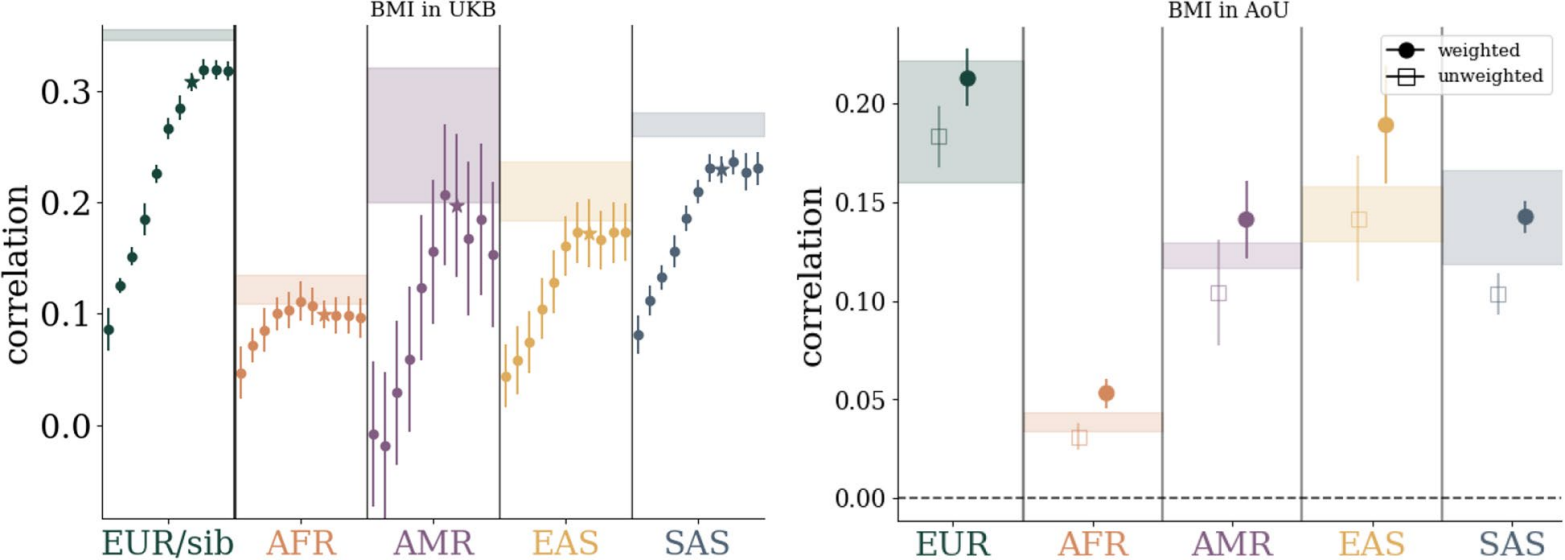
Left: performance as a function of training SNV size in UKB and applied to different ancestry groups. Within each ancestry group, dots correspond to training with $\{10, 23, 50, 100, 227, 500, 1000, 2273, 5000, 10 000, 22727\}$ SNVs per chromosome from left to right respectively. The starred data points correspond to 2273 SNVs per chromosome which is roughly equivalent to 50k SNVs across the autosome. Right: performance before and after the re-weighting step of the blockLASSO. While re-weighting is trained within the EUR group, the effect of re-weighting improves prediction accuracy across all tested ancestry groupings. Colored bands indicate one standard deviation bounds for the global result. For the UKB this includes a contribution from 5 CV folds and finite sample sizes, but for AoU it only includes the finite sample contribution

For the global approach it has been previously shown that the top 50k SNVs are more than sufficient to build a PGS [24, 25]. It was shown that the resultant sparse PGS included SNVs all throughout the top 50k SNVs, but that increasing the total number of SNVs (e.g., to 100k [24]) did not noticeably improve results, even for the least sparse predictors. For the block approach, the ideal number of features is not a priori known. It will not necessarily be the case that the set of SNVs from the 50k top ranked SNVs *across the entire autosome* will be the same set as the collection of the top 2,273 SNVs on each autosomal chromosome. To this end we tested different block sizes: {10; 23; 50; 100; 227; 500; 1,000; 2,273; 5,000; 10,000; 22,727} SNVs. The performance of training with different block sizes within the UKB can be seen in Fig. 5 and in the Supplementary Information. We see that performance tends to plateau near 2,273 SNVs per chromosome (50,006 SNVs over the entire autosome) which is roughly equivalent to the size of the global LASSO training. This plateau behavior generally persists when tested in other ancestry groups.

After feature selection, step (4) involves running the LASSO algorithm. We used Scikit-Learn with settings specified in Supplementary Information. Model selection is done by selecting the maximal performance in the validation/model-selection set. The LASSO algorithm can be described by minimizing the objective function

1 $$\begin{aligned} \mathcal {O}(\lambda ) = \frac{1}{2N}||\vec {y^*}-\bar{X}\cdot \vec {\beta }||^2_{L_2}+\lambda |\vec {\beta }|_{L_1} \, , \end{aligned}$$

where $\vec {y^*}$ is the residualized phenotype, $\bar{X}$ is the genotype matrix, $\vec {\beta }$ are the LASSO weights, and $\lambda$ is the LASSO hyper-parameter. If we expand the first term we see that the objective function is a sum of effects at each feature (SNV) location along with a term $\beta ^T X^TX\beta$ which encodes the feature correlation information. In the blockLASSO procedure we assume that this correlation structure is block diagonal so that we can minimize the objective function for each block and then compute the relative normalization between the blocks. Additional details for how the LASSO was implemented can be found in the Supplementary Information and in the accompanying code examples.

Step (5) is only for the blockLASSO. Using the validation/model-selection set we now do a linear regression of block scores against the raw phenotype. In principle, the re-weighting does not need to be done with linear regression, but because there are so few features in the current example (i.e., 22 chromosomes), testing with more advanced algorithms yielded the same results as linear regression. An example of the effect of this re-weighting, for all 5 ancestry groups, can be seen on the right side of Fig. 5.

Finally, in step (6) the models (i.e., individual SNV weights *and* block weights) are applied to the testing sets, which have been withheld from all training steps. A model from each cross-validation fold is applied to the final testing set to create a distribution and estimate the uncertainty in the model.

Computational resources used in both the blockLASSO and global approach were monitored for the UKB calculations. The computing cluster used for this analysis uses a SLURM [49] workload manager and the computational details were tracked via the seff command.

To cross-check the pipeline we used simulated genotypes and phenotypes. We generated simulated genotype data for 20,000 samples using *HaploDynamics* [50]. We generated data for 22 chromosomes with 5,001 features per chromosome. Each chromosome had a random starting position, a total size of 100 megabase pairs (Mbp), and 50 genetic positions per region (set with the “schema” parameter). We set the LD strength parameter to 1 for maximal LD within each chromosome and the population parameter to 0.1 to set the scale of the allele frequency distribution. To simulate the phenotype we used a simple linear model: phenotype = true_linear_effects + error. On each simulated chromosome, *X*, we selected 250 representative features and then a subset of these features were selected and randomly given true effect weights, $\beta$ from a uniform distribution effect distribution, $U_{\left[ 0,1\right) }$. We added to this an error term that was drawn from a uniform effect distribution of the size of the true genetic effects, but with an arbitrary scale to set the desired heritability: $a\times U_{\left[ 0,\sigma _{\beta \cdot X}\right) }$. As seen in Table 2 we simulated phenotypes with heritability ${\sim }33\%$ and ${\sim }75\%$ to check the pipeline. These heritabilities were selected as there is a large history of literature recording human phenotype heritabilties in this range [51]. Similarly we selected the number of true weights (50 and 250) because this corresponded to relative polygenicities of LASSO based predictors commonly found in previous work [25].

**Table 2:** Block vs global pipeline on simulated data. All results here presented involve 250 features per block and 2,500 samples

N true	Error	Heritability	Global LASSO	Global	True	BlockLASSO	Unweighted block	Weighted block
Weights	Scale		Memory (MB)	Correlation	Correlation	Memory (MB)	Correlation	Correlation
50	2	$0.75_{0.02}$	2.209	$0.86_{0.03}$	$0.86_{0.01}$	0.102	$0.59_{0.05}$	$0.77_{0.04}$
50	5	$0.32_{0.02}$	5.073	$0.52_{0.05}$	$0.56_{0.02}$	0.101	$0.26_{0.06}$	$0.42_{0.06}$
250	2	$0.75_{0.02}$	4.662	$0.75_{0.04}$	$0.87_{0.01}$	0.101	$0.48_{0.06}$	$0.58_{0.05}$
250	5	$0.34_{0.02}$	4.595	$0.31_{0.06}$	$0.58_{0.02}$	0.101	$0.16_{0.06}$	$0.27_{0.06}$

### PGS performance uncertainties

For almost all of the LASSO approaches, the reported uncertainty is a standard deviation (i.e., reflects the width of the overall possible distribution) and has contributions from 5-fold cross-validation and from computing metrics with finite sample sizes. These two effects are added in quadrature.

2 $$\begin{aligned} \sigma _{\text {AUC}} \approx \sqrt{\left( \frac{1}{3 \sqrt{N_{cases}}}\right) ^2 + \sigma _{cv-SD}^2} \qquad \text {and} \qquad \sigma _{corr.} = \sqrt{\left( \sqrt{\frac{1-\rho ^2}{N-2}}\right) ^2+\sigma _{cv-SD}^2}\, , \end{aligned}$$

where $N_{cases}$ is the number of cases used in training, $\rho$ is the correlation, *N* is the total number of samples (people) used in training, and $\sigma _{cv-SD}$ is the standard deviation over cross-validation folds. The first factor in the AUC uncertainty is empirically determined in [17] and is roughly equivalent with the theoretical result for standard error of AUC computed with exactly Gaussian distributions [52]. We find this approach to be more conservative (i.e., have larger error bars) than simply reporting a confidence interval and make it easier to cross-check results when training with different representative groups in the same biobank.

The only exception is the global LASSO in AoU which is both slow and costly. For this alone we only use a single global LASSO run to compute metrics and the uncertainties are reported as the larger of either the uncertainty from the finite sample size computation of the metric *or* the uncertainty in the UKB with the same training size. Specific definitions for the finite sample size contributions are given in the Supplementary Information.

### PGS variance distributions

In order to estimate the localized contribution to the variance of the PGS we compute an approximate covariance per SNV (feature). To do this we compute the covariance between each feature and sum the contribution from all correlated features. I.e., we build a covariance matrix of features and sum over columns. The sum of the partially summed covariance is the total variance of the PGS. Within each block, the encoded genotype can be written $^{b}X_{i,j}$ where *b* labels the block, *i* labels the sample, and *j* labels the feature (SNV). We then include the weights for each feature, $^{b}{\beta _j}$, and each block, $\alpha _b$: $\alpha _b ^{b}{\beta _j} ^{b}{X_{i,j}}\equiv ^{b}{H_{i,j}}$. Finally we compute the covariance matrix

3 $$\begin{aligned} K_{j,k} = COV(\vec {H}_{j},\vec {H}_{k})\, , \end{aligned}$$

where $\vec {H}_{j}$ is the column of sample values for the $j^{th}$ feature. Note that when computing the covariance between features (SNVs) on different blocks the covariance is assumed 0 by definition.

This approximate variance per locus is a generalized version of “single SNP variance” defined in [17, 32]. When training with *n* samples (e.g., people) and *m* total features (e.g., all SNVs from all blocks), the covariance between features is an $m\times m$ matrix. In Fig. 4, and in the Supplementary Information, we see the result of summing over one axis in the covariance matrix. To make the plot more human-readable we filter out variants accounting for less than 0.01% of the total variance and by binning nearby variants within 1Mbp. After cutting and binning we attribute this partial sum to the effective location of the binned variants (a weighted average over variant location). Error bars are computed by averaging over cross-validation folds.

## Discussion

There are many successful methods for training PGS. Generally, compared to methods that rely on summary statistics or genome wide associations, methods that train directly on genotypes and involve variant/nucleotide level data can produce equivalent (or better) results with much less data (e.g., human height prediction using ${\sim }450$k individuals in [24] vs ${\sim }5.4$ million individuals [53]). Additionally, many of the limitations associated with PGS in general are actually limitations of *GWAS-based* PGS (e.g., [54]). There is an added interest in *sparse* methods, i.e., algorithms that perform feature selection, allowing inferences regarding genetic architecture and pleiotropy. Currently, most commonly used sparse methods perform similarly, with the simple least absolute shrinkage and selection operator (LASSO) regularly performing among the best methods [17]. Sparse methods are often advantageous as performing feature selection can reduce the computational demand. However, training directly on genotypes – even after pre-filtering SNVs – is still very demanding and typically requires hundreds of gigabytes of memory and tens of hours of run time on a computing cluster. Here we present an improved implementation which makes use of block diagonal structure in SNP correlations associated with independent chromosomes (or other large regions). For a PGS that trains with 50k SNPs and 450k samples, this produces a ${\sim }75$x reduction in required memory and compute resources. While the approach advocated here is seemingly very simple, we caution that it is not trivial. As described in the methods section, the block approach appears to be more sensitive to any pre-filtering of variants as a poor initial selection appears to lead to over-fitting.

Because the proposed block procedure selects features *per block*, it is true that the block approach will not necessarily use the same features as the global approach of comparable total size. The advantages of using a block procedure are that you can achieve a speed up (with the total number, but different variants), you can use more total variants by using large blocks, or you can combine both advantages. The obvious follow up question is: if you are using a possibly different set of variants (slightly different selection procedure) and not training directly on the correlation structure (block vs global), can you still recover a meaningful PGS? Because we find similar overall metrics and because the variance explained by features is similar, we feel we have answered this question in the affirmative.

LASSO based predictors have previously been trained in one biobank and tested in a separate biobank with only minimal loss in performance (e.g., [25]). It is encouraging here that we see training and testing a polygenic predictor is largely independent of dataset (i.e. AoU vs UKB) – given equal data size. This suggests confounders – such as intake, genotyping platform/procedure, societal differences, etc. – have diminutive effect on the interpretations of these LASSO based PGS trained directly on genotypes.

The main use case for this new approach is methods development and exploratory research in developing PGS for new phenotypes. Newer biobanks, like AoU, are becoming reliant on third-party cloud computing. Even older biobanks like the UKB are changing their policies to mandate usage of these third party resources. For individual researchers, these third-party resources, while widely accessible, can be very costly compared to university based high performance computing clusters where the cost is typically a one time investment in hardware. As illustrative example, consider [25] where LASSO based PGS were trained for 16 phenotypes. For each phenotype there were 5 cross-validation folds trained. Because LASSO based methods were not widely used at the time this was exploratory research and best practices were not obvious this full training procedure was done several times for each phenotype: e.g., with and without particular covariates (at least 1 additional run per phenotype), mistakes (at least an additional run per phenotype), and at least as many additional phenotypes were tested but not presented in the research (16 additional phenotypes. This leads conservatively leads to $(16+16)\times5\times2\times2=640$ training runs. We can estimate that PGS were trained with at least 600GB of memory for roughly 16 hours. Using current Google Cloud (in use for AoU and future UKB projects) compute engine pricing [55] we can estimate this research would have cost an individual research group: $16\times\$5.69\times640\approx \$58,000$. While this pales in comparison to the cost in actually building a biobank it can be a prohibitive cost to researchers interested in methods development.

We hope that the novel implementation demonstrated in this work can be improved upon. Screening rules – i.e., rules which aid in feature selection – have only resulted in modest improvements in the performance of global LASSO approaches. For example, as shown in the supplement, a global LASSO using the top 50k commons SNPs selected by p-value in the UKB achieves a height PGS correlation of ${\sim }0.63$. Using the bigstatsr package to extend this to 650k common SNPs that pass basic quality controls raises the correlation to ${\sim }0.645$. These are both still short of the saturated estimated of common SNP correlation of ${\sim }0.72$ as shown in [53]. However, it is almost certain that incorporating screening will further speed up block approaches. Some PGS models have been improved via ancestry specific tagging and SNV selection [56, 57], functional information [58–61], and more careful definitions of phenotypes [62–64]. It should be possible to integrate all of these approaches with the new implementation presented here.

Finally we mention that initial research has shown the implementation of PGS in the clinic can lead to significantly better outcomes for some diagnoses [65]. There is also a large literature highlighting potential benefits for identifying those at high risk of particular diseases, aiding in early detection, reducing total cost of care. This is all evidence for PGS as a generally useful tool in clinical practice [25, 66–71]. However, there are still outstanding practical [72–76] and ethical [77–79] challenges related to the widespread adoption of PGS to the practice of clinical medicine.

## Conclusions

This work details the construction of a block-by-block polygenic score using LASSO on individual chromosomes. This new implementation, blockLASSO, generates polygenic scores with similar performance to traditional methods but using orders of magnitude less computer memory and orders of magnitude less time. This new efficient method of polygenic score construction will be useful as more genomic analyses move to cloud computing and the cost of computing traditional polygenic scores becomes prohibitive.

## Supplementary Information

Supplementary Material 1

## Data Availability

Block predictors from both the UKB and AoU and code examples can be found in the Hsu group GitHub: https://github.com/MSU-Hsu-Lab/blockLASSO. Exact code used to generate the AoU results can be found in the “Scalable and efficient polygenic scores in diverse populations” workspace in the AoU “controlled tier” data access tier.

## Abbreviations

AFR: African
AMR: American
AoU: All of Us
AUC: Area under receiver operator curve
BASIL: Batch screening iterative LASSO
BMI: Body mass index
EAS: East Asian
EUR: European
GB: Gigabyte
GWAS: Genome wide association studies
hdl: High-density lipoprotein
LASSO: Least absolute shrinkage and selection operator
LD: Linkage disequilibrium
Mbp: Megabase pair
PGS: Polygenic score
SAS: South American
SNP: Single nucleotide polymorphism
SNV: Single nucleotide variant
T1D: Type 1 diabetes
T2D: Type 2 diabetes
UKB: UK Biobank
