# Supplementary material for: Efficient blockLASSO for polygenic scores with applications to All of Us and UK Biobank
Source: BMC Genomics. 2025 Mar 27;26:302. doi: 10.1186/s12864-025-11505-0 (PMC11948729; doi:10.1186/s12864-025-11505-0)
Supplement: Supplementary file 1 — Supplementary Material 1 [file 12864_2025_11505_MOESM1_ESM.pdf]

Efficient Block Lasso for building polygenic scores with applications to the UK Biobank and All of Us

Timothy G. Raben, Louis Lello, Erik Widen, Stephen D.H. Hsu

January 24, 2025

Contents

1 Data **2**

2 Polygenic score computation **16**

3 Simulations and Benchmarking **19**

4 PGS metrics plots **20**

5 Variance plots **21**

6 Training and re-weighting **27**

7 LASSO validation paths **33**

1 Data

Phenotypes from the UK Biobank (UKB) are based upon the November 20th, 2023 release of UKB data. Case control codes are made from `Non-cancer illness code, self-reported`, `Diagnoses - ICD9`, and `Diagnoses - ICD10` codes. Exact sample counts can be found in **Supplemental Table 1**. As described in the methods section, phenotypes are residualized against covariates. The residual distributions can be seen in **Supplemental Figure 1-Supplemental Figure 9**. While hdl, BMI, and total bilirubin have significant right tails, they are not well modeled by a log-normal distribution as can be seen in **Supplemental Figure 10-Supplemental Figure 12**. The following definitions are used in the UKB:

type 2 diabetes non-cancer codes: 1223
- ICD9: 25000, 25002, 25010, 25012, 25020, 25022, 25030, 25032, 25040, 25042, 25050, 25052, 25060, 25062, 25070, 25072, 25080, 25082, 25090, 25092
- ICD10: E11, E110, E111, E112, E113, E114, E115, E116, E117, E118, E119

asthma non-cancer codes: 1111
- ICD9: 49300, 49309 ,49310 ,49319 ,49390 ,49399
- ICD10: J450,J451,J458

gout noncancer: 1466
- ICD9: 2740, 2741, 2748, 2749, 7120
- ICD10: M1000, M1001, M1002, M1003, M1004, M1005, M1006, M1007, M1008, M1009, E790

psoriasis noncancer: 1453
- ICD9: 6961, 6962, 6968
- ICD10: L400, L401, L404, L405, L408, L409, L413, L414,L415, L418, L419, M0900, M0901, M0902, M0903,M0904, M0905, M0906, M0907, M0908, M0909

hyperlipidemia field ID 30690 (total cholesterol) with value ≥ 6.21 as case

type 1 diabetes noncancer: 1222
- ICD10: E100, E101, E102, E103, E104, E105, E106, E107, E108, E109, O240

hypertension noncancer: 1065,1072,1073
- ICD9: 4010, 4011, 4019, 4050, 4051, 4059, 4160, 6420, 6423, 6429
- ICD10: I10

height field ID: 50

bmi field ID: 21001

total bilirubin field ID: 30840

hdl field ID: 30760

phenotype	training		model selection		testing	
	cases	controls	cases	controls	cases	controls
asthma	48,775	151,225	1,250	1,250	4,589	35,499
gout	7,188	192,812	1,250	1,250	795	39,293
hyperlipidemia	125,414	272,412	1,250	1,250	12,477	27,611
hypertension	167,219	249,072	1,250	1,250	16,039	24,049
psoriasis	7,740	192,260	1,250	1,250	906	39,182
type 1 diabetes	2,629	197,371	1,250	1,250	368	39,720
type 2 diabetes	29,588	170,412	1,250	1,250	2,833	37,255

phenotype	training	model selection	testing
bmi	415,082	2,500	40,088
hdl	364,809	2,500	40,088
height	414,960	2,500	40,088
total bilirubin	396,219	2,500	40,088

Supplemental Table 1: Phenotype sample sizes in UKB. For case control conditions with less than 50k cases, the controls were limited to keep a total of 200k samples to speed up training.

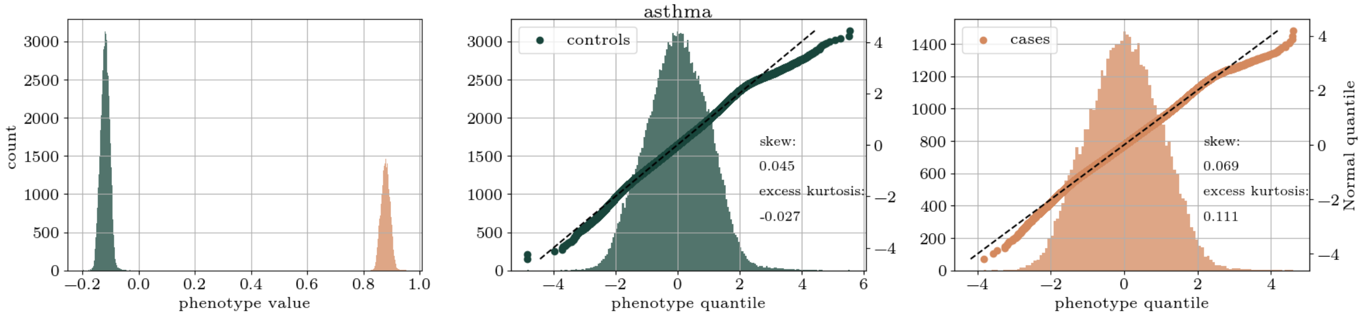

Supplemental Figure 1: Residual distribution of the UKB asthma phenotype.

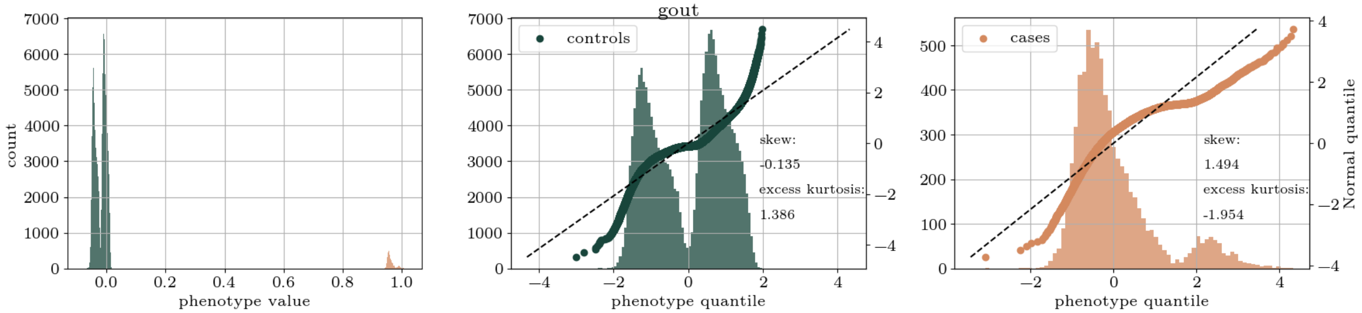

Supplemental Figure 2: Residual distribution of the UKB gout phenotype.

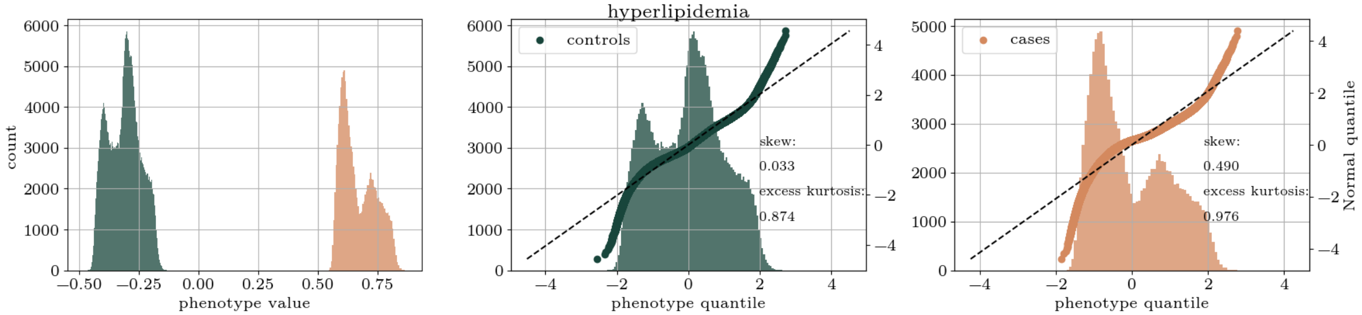

Supplemental Figure 3: Residual distribution of the UKB hyperlipidemia phenotype.

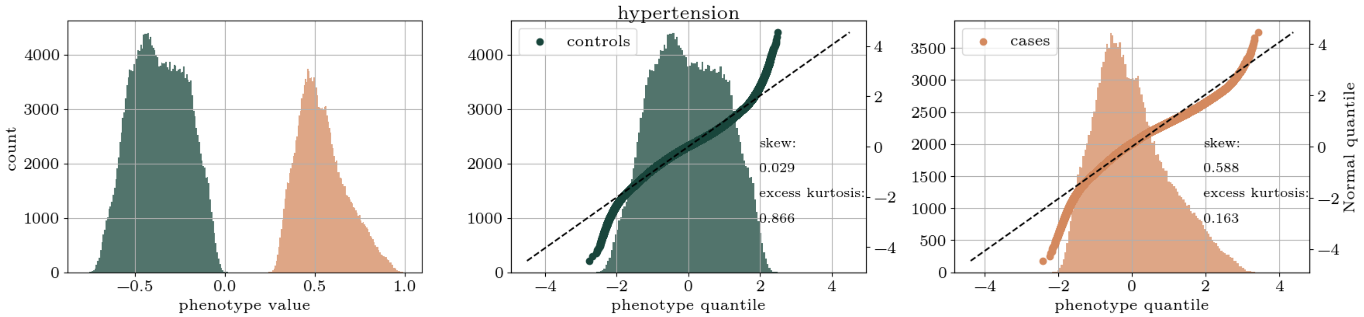

Supplemental Figure 4: Residual distribution of the UKB hypertension phenotype.

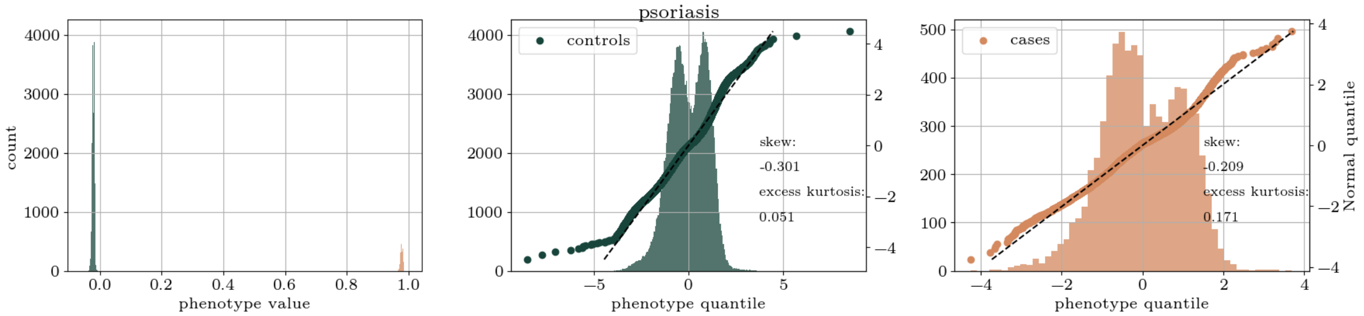

Supplemental Figure 5: Residual distribution of the UKB psoriasis phenotype.

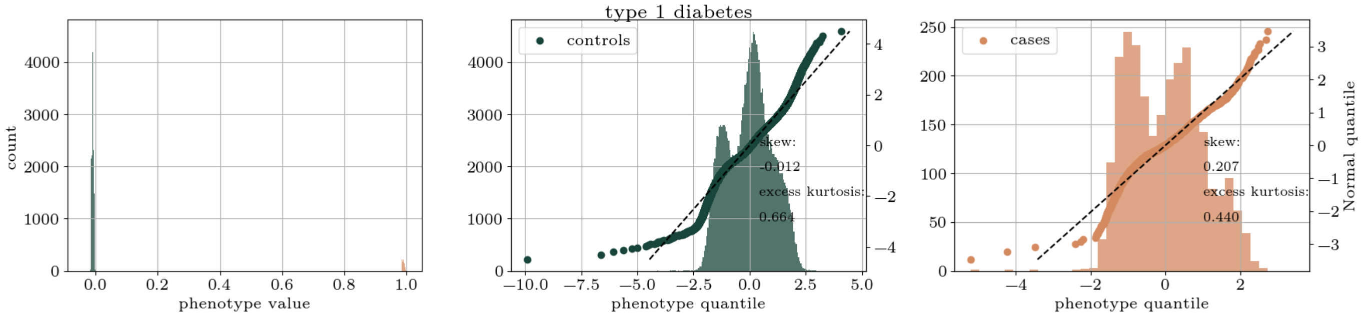

Supplemental Figure 6: Residual distribution of the UKB type 1 diabetes phenotype.

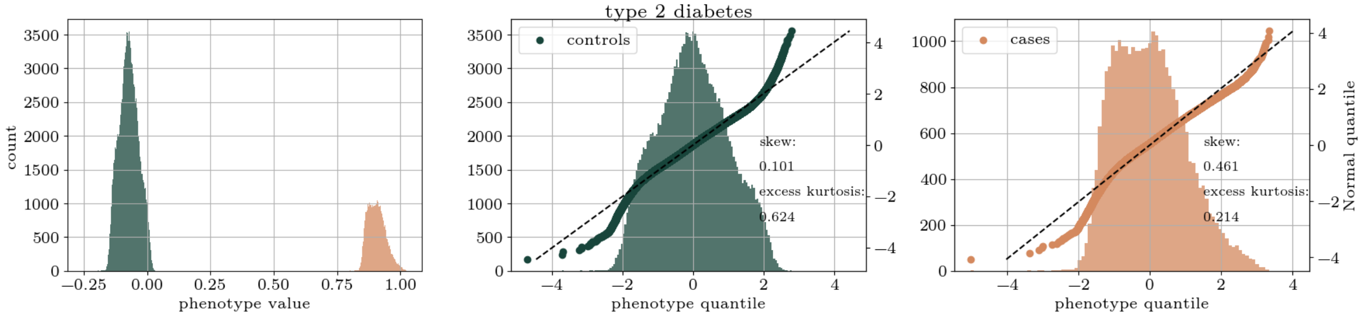

Supplemental Figure 7: Residual distribution of the UKB type 2 diabetes phenotype.

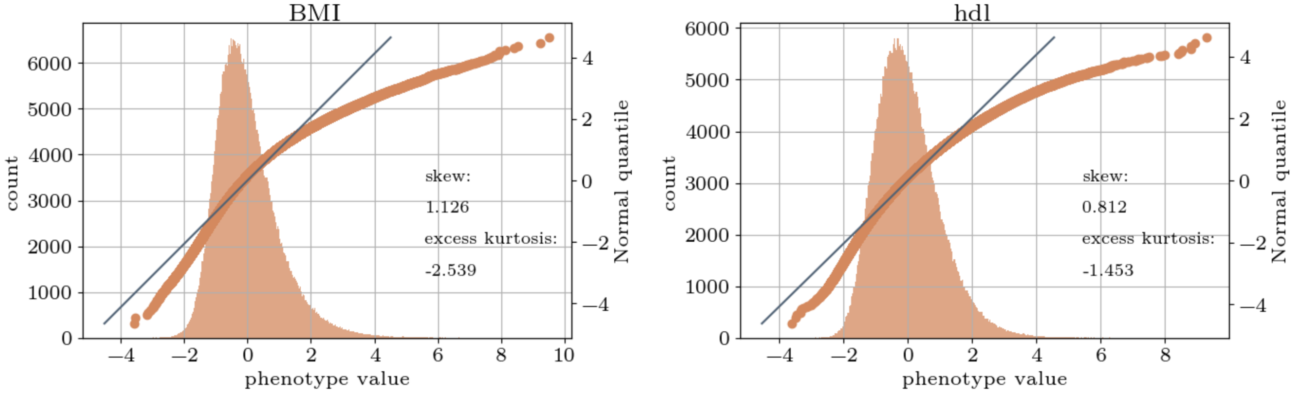

Supplemental Figure 8: Residual distribution of the UKB bmi and hdl phenotypes.

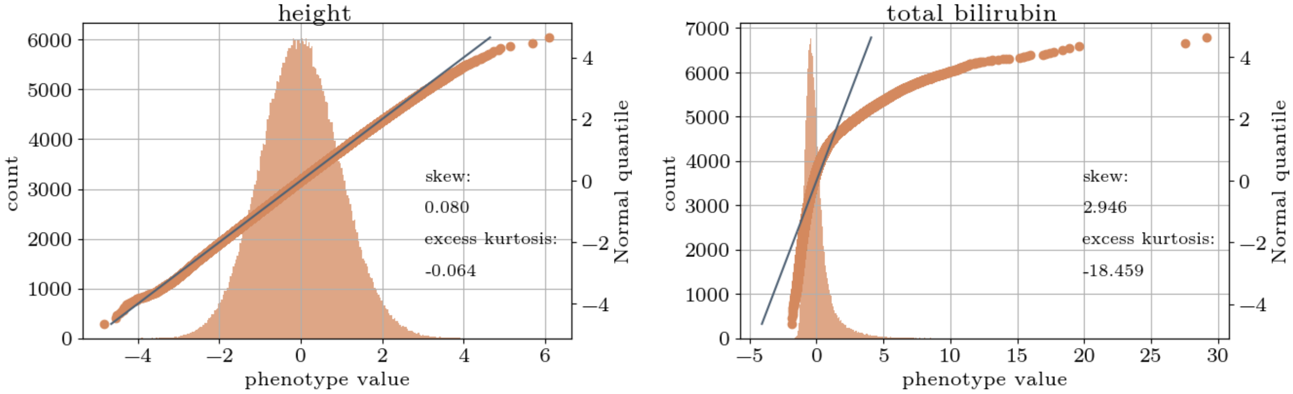

Supplemental Figure 9: Residual distribution of the UKB height and total bilirubin phenotypes.

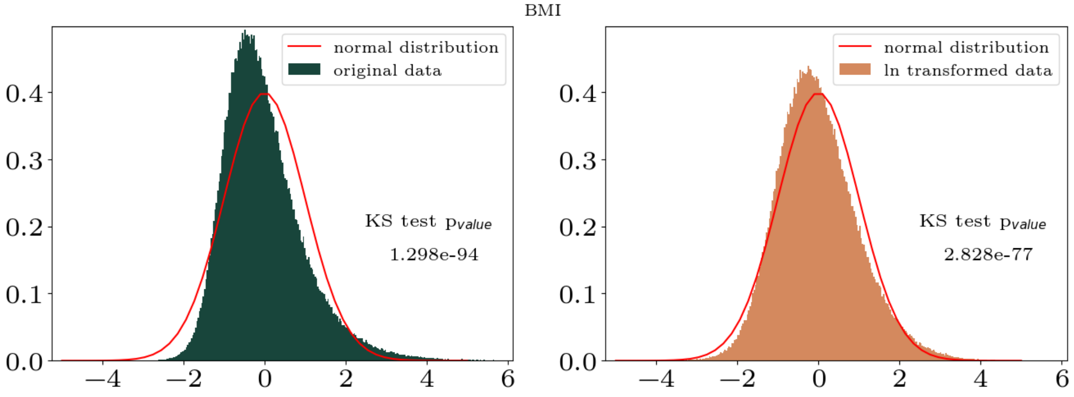

Supplemental Figure 10: Normal and log-normal Kolmogorov-Smirnov tests show that the BMI phenotype is not well modeled with either distribution.

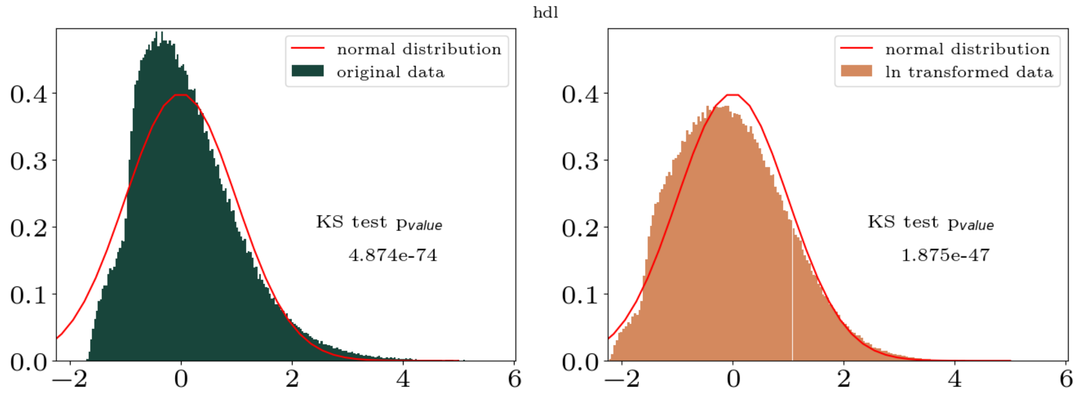

Supplemental Figure 11: Normal and log-normal Kolmogorov-Smirnov tests show that the hdl phenotype is not well modeled with either distribution.

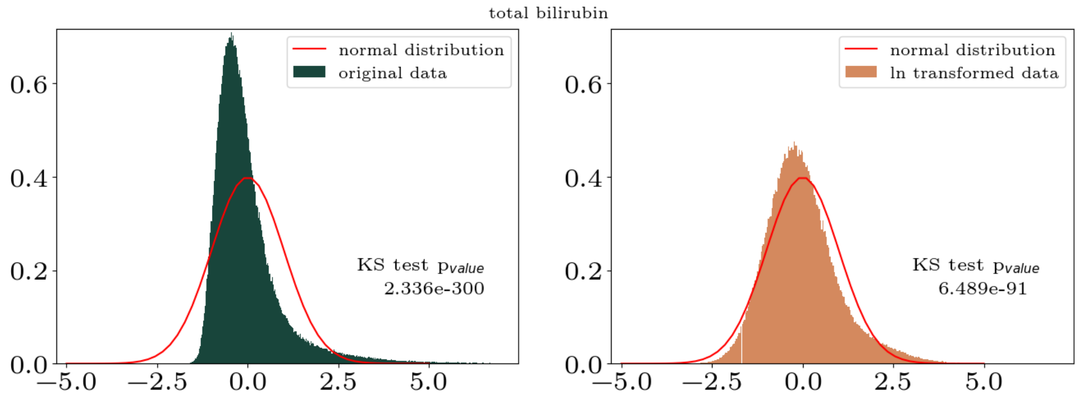

Supplemental Figure 12: Normal and log-normal Kolmogorov-Smirnov tests show that the total bilirubin phenotype is not well modeled with either distribution.

Phenotypes from AoU were constructed using the AoU workbench. Exact sample sizes can be found in **Supplemental Table 2**. As described in the methods section, phenotypes are residualized against covariates. The residual distributions can be seen in **Supplemental Figure 13-Supplemental Figure 21**. Using keyword searchers, most case control phenotypes all included information from “All Surveys” data and continuous phenotypes can be found in the “physical measurements” survey data. Additionally, the following concepts were used in combination with the survey results.

type 2 diabetes Standard Concepts: Acidosis due to type 2 diabetes mellitus; Angina associated with type 2 diabetes mellitus; Arthropathy due to type 2 diabetes mellitus; Autonomic neuropathy due to type 2 diabetes mellitus; Cataract due to diabetes mellitus type 2; Chronic kidney disease due to type 2 diabetes mellitus; Chronic kidney disease stage 2 due to type 2 diabetes mellitus; Chronic kidney disease stage 3 due to type 2 diabetes mellitus; Chronic kidney disease stage 4 due to type 2 diabetes mellitus; Chronic kidney disease stage 5 due to type 2 diabetes mellitus; Coronary artery disease due to type 2 diabetes mellitus; Dermopathy due to type 2 diabetes mellitus; Diabetes mellitus type 2 without retinopathy; Disorder due to type 2 diabetes mellitus; Disorder due to well controlled type 2 diabetes mellitus; Disorder of eye due to type 2 diabetes mellitus; Disorder of nervous system due to type 2 diabetes mellitus; Dyslipidemia due to type 2 diabetes mellitus; End stage renal disease on dialysis due to type 2 diabetes mellitus; Erectile dysfunction due to type 2 diabetes mellitus; Foot ulcer due to type 2 diabetes mellitus; Gangrene due to type 2 diabetes mellitus; Gastroparesis due to type 2 diabetes mellitus; Hyperglycemia due to type 2 diabetes mellitus; Hyperosmolar coma due to type 2 diabetes mellitus; Hyperosmolar non-ketotic state due to type 2 diabetes mellitus; Hypoglycemia due to type 2 diabetes mellitus; Hypoglycemic coma due to type 2 diabetes mellitus; Insulin treated type 2 diabetes mellitus; Ketoacidosis due to type 2 diabetes mellitus; Ketoacidotic coma due to type 2 diabetes mellitus; Macular edema and retinopathy due to type 2 diabetes mellitus; Macular edema due to type 2 diabetes mellitus; Microalbuminuria due to type 2 diabetes mellitus; Mild nonproliferative retinopathy due to type 2 diabetes mellitus; Mixed hyperlipidemia due to type 2 diabetes mellitus; Moderate nonproliferative retinopathy due to type 2 diabetes mellitus; Mononeuropathy due to type 2 diabetes mellitus; Multiple complications due to type 2 diabetes mellitus; Neuropathic arthropathy due to type 2 diabetes mellitus; Neuropathy due to type 2 diabetes mellitus; Nonproliferative retinopathy due to type 2 diabetes mellitus; Peripheral circulatory disorder due to type 2 diabetes mellitus; Peripheral neuropathy due to type 2 diabetes mellitus; Peripheral sensory neuropathy due to type 2 diabetes mellitus; Polyneuropathy due to type 2 diabetes mellitus; Pre-existing type 2 diabetes mellitus; Pre-existing type 2 diabetes mellitus in pregnancy; Pregnancy and type 2 diabetes mellitus; Proliferative retinopathy due to type 2 diabetes mellitus; Proteinuria due to type 2 diabetes mellitus; Renal disorder due to type 2 diabetes mellitus;

phenotype	training		model selection		testing	
	cases	controls	cases	controls	cases	controls
asthma	20,866	90,931	1,000	1,000	1,000	1,000
gout	2,477	112,320	250	250	250	250
hyperlipidemia	51,013	60,784	1,000	1,000	1,000	1,000
hypertension	46,371	65,426	1,000	1,000	1,000	1,000
psoriasis	2,588	84,269	500	500	500	500
type 1 diabetes	1,500	113,297	250	250	250	250
type 2 diabetes	15,522	96,275	1,000	1,000	1,000	1,000

phenotype	training	model selection	testing
bmi	102,949	5,000	5,000
hdl	44,665	5,000	5,000
height	103,202	5,000	5,000
total bilirubin	56,776	5,000	5,000

Supplemental Table 2: Phenotype sample sizes in AoU

Retinal edema due to type 2 diabetes mellitus; Retinopathy due to type 2 diabetes mellitus; Traction detachment of retina due to type 2 diabetes mellitus; Type 2 diabetes mellitus; Type 2 diabetes mellitus controlled by diet; Type 2 diabetes mellitus in nonobese; Type 2 diabetes mellitus in obese; Type 2 diabetes mellitus well controlled; Type 2 diabetes mellitus with peripheral angiopathy; Type 2 diabetes mellitus with ulcer; Type 2 diabetes mellitus without complication; Ulcer of heel due to type 2 diabetes mellitus; Ulcer of left foot due to type 2 diabetes mellitus; Ulcer of lower limb due to type 2 diabetes mellitus; Ulcer of right foot due to type 2 diabetes mellitus; Ulcer of toe due to type 2 diabetes mellitus

Source Concepts: Pre-existing type 2 diabetes mellitus, in childbirth; Pre-existing type 2 diabetes mellitus, in pregnancy; Pre-existing type 2 diabetes mellitus, in pregnancy, childbirth and the puerperium; Pre-existing type 2 diabetes mellitus, in pregnancy, first trimester; Pre-existing type 2 diabetes mellitus, in pregnancy, second trimester; Pre-existing type 2 diabetes mellitus, in pregnancy, third trimester; Pre-existing type 2 diabetes mellitus, in pregnancy, unspecified trimester; Pre-existing type 2 diabetes mellitus, in the puerperium; Type 2 diabetes mellitus; Type 2 diabetes mellitus with circulatory complications; Type 2 diabetes mellitus with diabetic amyotrophy; Type 2 diabetes mellitus with diabetic arthropathy; Type 2 diabetes mellitus with diabetic autonomic (poly)neuropathy; Type 2 diabetes mellitus with diabetic cataract; Type 2 diabetes mellitus with diabetic chronic kidney disease; Type 2 diabetes mellitus with diabetic dermatitis; Type 2 diabetes mellitus with diabetic macular edema, resolved following treatment; Type 2 diabetes mellitus with diabetic macular edema, resolved following treatment, bilateral; Type 2 diabetes mellitus with diabetic macular edema, resolved following treatment, left eye; Type 2 diabetes mellitus with diabetic macular edema, resolved following treatment, right eye; Type 2 diabetes mellitus with diabetic macular edema, resolved following treatment, unspecified eye; Type 2 diabetes mellitus with diabetic mononeuropathy; Type 2 diabetes mellitus with diabetic nephropathy; Type 2 diabetes mellitus with diabetic neuropathic arthropathy; Type 2 diabetes mellitus with diabetic neuropathy, unspecified; Type 2 diabetes mellitus with diabetic peripheral angiopathy with gangrene; Type 2 diabetes mellitus with diabetic peripheral angiopathy without gangrene; Type 2 diabetes mellitus with diabetic polyneuropathy; Type 2 diabetes mellitus with foot ulcer; Type 2 diabetes mellitus with hyperglycemia; Type 2 diabetes mellitus with hyperosmolarity; Type 2 diabetes mellitus with hyperosmolarity with coma; Type 2 diabetes mellitus with hyperosmolarity without nonketotic hyperglycemic-hyperosmolar coma (NKHHC); Type 2 diabetes mellitus with hypoglycemia; Type 2 diabetes mellitus with hypoglycemia with coma; Type 2 diabetes mellitus with hypoglycemia without coma; Type 2 diabetes mellitus with ketoacidosis; Type 2 diabetes mellitus with ketoacidosis with coma; Type 2 diabetes mellitus with ketoacidosis without coma; Type 2 diabetes mellitus with kidney complications; Type 2 diabetes mellitus with mild nonproliferative diabetic retinopathy; Type 2 diabetes mellitus with mild nonproliferative diabetic retinopathy with macular edema; Type 2 diabetes mellitus with mild nonproliferative diabetic retinopathy with macular edema, bilateral; Type 2 diabetes mellitus with mild nonproliferative diabetic retinopathy with macular edema, left eye; Type 2 diabetes mellitus with mild nonproliferative diabetic retinopathy with macular edema, right eye; Type 2 diabetes mellitus with mild nonproliferative diabetic retinopathy with macular edema, unspecified eye; Type 2 diabetes mellitus with mild nonproliferative diabetic retinopathy without macular edema; Type 2 diabetes mellitus with mild nonproliferative diabetic retinopathy without macular edema, bilateral; Type 2 diabetes mellitus with mild nonproliferative diabetic retinopathy without macular edema, left eye; Type 2 diabetes mellitus with mild nonproliferative diabetic retinopathy without macular edema, right eye; Type 2 diabetes mellitus with mild nonproliferative diabetic retinopathy without macular edema, unspecified eye; Type 2 diabetes mellitus with moderate nonproliferative diabetic retinopathy; Type 2 diabetes mellitus with moderate nonproliferative diabetic retinopathy with macular edema; Type 2 diabetes mellitus with moderate nonproliferative diabetic retinopathy with macular edema, bilateral; Type 2 diabetes mellitus with moderate nonproliferative diabetic retinopathy with macular edema, left eye; Type 2 diabetes mellitus with moderate nonproliferative diabetic retinopathy with macular edema, right eye; Type 2 diabetes mellitus with moderate nonproliferative diabetic retinopathy with macular edema, unspecified eye; Type 2 diabetes mellitus with moderate nonproliferative diabetic retinopathy without

macular edema; Type 2 diabetes mellitus with moderate nonproliferative diabetic retinopathy without macular edema, bilateral; Type 2 diabetes mellitus with moderate nonproliferative diabetic retinopathy without macular edema, left eye; Type 2 diabetes mellitus with moderate nonproliferative diabetic retinopathy without macular edema, right eye; Type 2 diabetes mellitus with moderate nonproliferative diabetic retinopathy without macular edema, unspecified eye; Type 2 diabetes mellitus with neurological complications; Type 2 diabetes mellitus with ophthalmic complications; Type 2 diabetes mellitus with oral complications; Type 2 diabetes mellitus with other circulatory complications; Type 2 diabetes mellitus with other diabetic arthropathy; Type 2 diabetes mellitus with other diabetic kidney complication; Type 2 diabetes mellitus with other diabetic neurological complication; Type 2 diabetes mellitus with other diabetic ophthalmic complication; Type 2 diabetes mellitus with other oral complications; Type 2 diabetes mellitus with other skin complications; Type 2 diabetes mellitus with other skin ulcer; Type 2 diabetes mellitus with other specified complication; Type 2 diabetes mellitus with other specified complications; Type 2 diabetes mellitus with periodontal disease; Type 2 diabetes mellitus with proliferative diabetic retinopathy; Type 2 diabetes mellitus with proliferative diabetic retinopathy with combined traction retinal detachment and rhegmatogenous retinal detachment; Type 2 diabetes mellitus with proliferative diabetic retinopathy with combined traction retinal detachment and rhegmatogenous retinal detachment, bilateral; Type 2 diabetes mellitus with proliferative diabetic retinopathy with combined traction retinal detachment and rhegmatogenous retinal detachment, left eye; Type 2 diabetes mellitus with proliferative diabetic retinopathy with combined traction retinal detachment and rhegmatogenous retinal detachment, right eye; Type 2 diabetes mellitus with proliferative diabetic retinopathy with combined traction retinal detachment and rhegmatogenous retinal detachment, unspecified eye; Type 2 diabetes mellitus with proliferative diabetic retinopathy with macular edema; Type 2 diabetes mellitus with proliferative diabetic retinopathy with macular edema, bilateral; Type 2 diabetes mellitus with proliferative diabetic retinopathy with macular edema, left eye; Type 2 diabetes mellitus with proliferative diabetic retinopathy with macular edema, right eye; Type 2 diabetes mellitus with proliferative diabetic retinopathy with macular edema, unspecified eye; Type 2 diabetes mellitus with proliferative diabetic retinopathy with traction retinal detachment involving the macula; Type 2 diabetes mellitus with proliferative diabetic retinopathy with traction retinal detachment involving the macula, bilateral; Type 2 diabetes mellitus with proliferative diabetic retinopathy with traction retinal detachment involving the macula, left eye; Type 2 diabetes mellitus with proliferative diabetic retinopathy with traction retinal detachment involving the macula, right eye; Type 2 diabetes mellitus with proliferative diabetic retinopathy with traction retinal detachment involving the macula, unspecified eye; Type 2 diabetes mellitus with proliferative diabetic retinopathy with traction retinal detachment not involving the macula; Type 2 diabetes mellitus with proliferative diabetic retinopathy with traction retinal detachment not involving the macula, bilateral; Type 2 diabetes mellitus with proliferative diabetic retinopathy with traction retinal detachment not involving the macula, left eye; Type 2 diabetes mellitus with proliferative diabetic retinopathy with traction retinal detachment not involving the macula, right eye; Type 2 diabetes mellitus with proliferative diabetic retinopathy with traction retinal detachment not involving the macula, unspecified eye; Type 2 diabetes mellitus with proliferative diabetic retinopathy without macular edema; Type 2 diabetes mellitus with proliferative diabetic retinopathy without macular edema, bilateral; Type 2 diabetes mellitus with proliferative diabetic retinopathy without macular edema, left eye; Type 2 diabetes mellitus with proliferative diabetic retinopathy without macular edema, right eye; Type 2 diabetes mellitus with proliferative diabetic retinopathy without macular edema, unspecified eye; Type 2 diabetes mellitus with severe nonproliferative diabetic retinopathy; Type 2 diabetes mellitus with severe nonproliferative diabetic retinopathy with macular edema; Type 2 diabetes mellitus with severe nonproliferative diabetic retinopathy with macular edema, bilateral; Type 2 diabetes mellitus with severe nonproliferative diabetic retinopathy with macular edema, left eye; Type 2 diabetes mellitus with severe nonproliferative diabetic retinopathy with macular edema, right eye; Type 2 diabetes mellitus with severe nonproliferative diabetic retinopathy with macular edema, unspecified eye; Type 2 diabetes mellitus with severe nonproliferative diabetic retinopathy without macular edema; Type 2 diabetes mellitus with severe nonproliferative diabetic retinopathy

without macular edema, bilateral; Type 2 diabetes mellitus with severe nonproliferative diabetic retinopathy without macular edema, left eye; Type 2 diabetes mellitus with severe nonproliferative diabetic retinopathy without macular edema, right eye; Type 2 diabetes mellitus with severe nonproliferative diabetic retinopathy without macular edema, unspecified eye; Type 2 diabetes mellitus with skin complications; Type 2 diabetes mellitus with stable proliferative diabetic retinopathy; Type 2 diabetes mellitus with stable proliferative diabetic retinopathy, bilateral; Type 2 diabetes mellitus with stable proliferative diabetic retinopathy, left eye; Type 2 diabetes mellitus with stable proliferative diabetic retinopathy, right eye; Type 2 diabetes mellitus with stable proliferative diabetic retinopathy, unspecified eye; Type 2 diabetes mellitus with unspecified complications; Type 2 diabetes mellitus with unspecified diabetic retinopathy; Type 2 diabetes mellitus with unspecified diabetic retinopathy with macular edema; Type 2 diabetes mellitus with unspecified diabetic retinopathy without macular edema; Type 2 diabetes mellitus without complications

asthma Standard Concept: Asthma

Source Concepts: Asthma; Asthma, unspecified; Asthma, unspecified type, unspecified; Asthma, unspecified type, with (acute) exacerbation; Asthma, unspecified type, with status asthmaticus; Chronic obstructive asthma; Chronic obstructive asthma with (acute) exacerbation; Chronic obstructive asthma with status asthmaticus; Chronic obstructive asthma, unspecified; Cough variant asthma; Cough variant asthma; Eosinophilic asthma; Extrinsic asthma; Extrinsic asthma with (acute) exacerbation; Extrinsic asthma with status asthmaticus; Extrinsic asthma, unspecified; Intrinsic asthma; Intrinsic asthma with (acute) exacerbation; Intrinsic asthma with status asthmaticus; Intrinsic asthma, unspecified; Mild intermittent asthma; Mild intermittent asthma with (acute) exacerbation; Mild intermittent asthma with status asthmaticus; Mild intermittent asthma, uncomplicated; Mild persistent asthma; Mild persistent asthma with (acute) exacerbation; Mild persistent asthma with status asthmaticus; Mild persistent asthma, uncomplicated; Moderate persistent asthma; Moderate persistent asthma with (acute) exacerbation; Moderate persistent asthma with status asthmaticus; Moderate persistent asthma, uncomplicated; Other and unspecified asthma; Other asthma; Other forms of asthma; Severe persistent asthma; Severe persistent asthma with (acute) exacerbation; Severe persistent asthma with status asthmaticus; Severe persistent asthma, uncomplicated; Unspecified asthma; Unspecified asthma with (acute) exacerbation; Unspecified asthma with status asthmaticus; Unspecified asthma, uncomplicated

gout Source Concepts: Gout due to renal impairment; Gout, unspecified; Idiopathic gout

psoriasis *cases:*

Source Concepts: Other psoriasis; Other psoriasis and similar disorders; Psoriasis; Psoriatic arthropathy

control exclusions:

Source Concepts: Adult-onset Still's disease; Bullous dermatoses; Bullous disorder, unspecified; Bullous disorders in diseases classified elsewhere; Chronic postrheumatic arthropathy [Jaccoud]; Congenital cutaneous mastocytosis; Congenital ichthyosis; Congenital malformation of skin, unspecified; Dermatitis factitia [artefacta]; Dermatitis herpetiformis; Dermatoglyphic anomalies; Discoid lupus erythematosus of eyelid; Discoid lupus erythematosus of eyelid; Dyschromia; Ectodermal dysplasia (anhidrotic); Epidermolysis bullosa; Erythema annulare centrifugum; Erythema in diseases classified elsewhere; Erythema intertrigo; Erythema marginatum; Erythema multiforme; Erythema nodosum; Erythematosquamous dermatosis; Erythematous condition, unspecified; Erythematous conditions; Exfoliation due to erythematous conditions according to extent of body surface involved; Exfoliative dermatitis; Factitial dermatitis; Febrile neutrophilic dermatosis [Sweet]; Felty's syndrome; Granuloma annulare; Ichthyosis congenita; Incontinentia pigmenti; Infantile (acute) (chronic) eczema; Infective dermatitis; Inflammatory polyarthropathy; Juvenile arthritis; Lennox-Gastaut syndrome; Lichen; Lichen nitidus; Lichen planopilaris; Lichen planus; Lichen simplex chronicus and prurigo; Lichen striatus; Lichenification and lichen simplex chronicus; Lupus erythematosus; Meningitis

in sarcoidosis; Other autoinflammatory syndromes; Other chronic figurate erythema; Other disorders of pigmentation; Other rheumatoid arthritis with rheumatoid factor; Other specified bullous disorders; Other specified congenital anomalies of skin; Other specified congenital malformations of skin; Other specified erythematous conditions; Other specified rheumatoid arthritis; Other vasculitis limited to the skin; Parapsoriasis; Pemphigoid; Pemphigus; Pityriasis alba; Pityriasis rosea; Pityriasis rubra pilaris; Poikiloderma of Civatte; Poikiloderma vasculare atrophicans; Polyarthritis, unspecified; Prurigo; Psoriasis; Psoriasis and similar disorders; Rheumatoid arthritis and other inflammatory polyarthropathies; Rheumatoid arthritis with involvement of other organs and systems; Rheumatoid arthritis with rheumatoid factor without organ or systems involvement; Rheumatoid arthritis with rheumatoid factor, unspecified; Rheumatoid arthritis without rheumatoid factor; Rheumatoid arthritis, unspecified; Rheumatoid bursitis, ankle and foot; Rheumatoid bursitis, elbow; Rheumatoid bursitis, hand; Rheumatoid bursitis, hip; Rheumatoid bursitis, knee; Rheumatoid bursitis, multiple sites; Rheumatoid bursitis, shoulder; Rheumatoid bursitis, unspecified site; Rheumatoid bursitis, wrist; Rheumatoid heart disease with rheumatoid arthritis of ankle and foot; Rheumatoid heart disease with rheumatoid arthritis of elbow; Rheumatoid heart disease with rheumatoid arthritis of hand; Rheumatoid heart disease with rheumatoid arthritis of knee; Rheumatoid heart disease with rheumatoid arthritis of multiple sites; Rheumatoid heart disease with rheumatoid arthritis of shoulder; Rheumatoid heart disease with rheumatoid arthritis of unspecified site; Rheumatoid heart disease with rheumatoid arthritis of wrist; Rheumatoid lung disease with rheumatoid arthritis; Rheumatoid myopathy with rheumatoid arthritis of ankle and foot; Rheumatoid myopathy with rheumatoid arthritis of hand; Rheumatoid myopathy with rheumatoid arthritis of multiple sites; Rheumatoid myopathy with rheumatoid arthritis of unspecified site; Rheumatoid nodule; Rheumatoid polyneuropathy with rheumatoid arthritis of hand; Rheumatoid polyneuropathy with rheumatoid arthritis of hip; Rheumatoid polyneuropathy with rheumatoid arthritis of knee; Rheumatoid polyneuropathy with rheumatoid arthritis of multiple sites; Rheumatoid polyneuropathy with rheumatoid arthritis of shoulder; Rheumatoid polyneuropathy with rheumatoid arthritis of unspecified site; Rheumatoid polyneuropathy with rheumatoid arthritis of wrist; Rheumatoid vasculitis with rheumatoid arthritis; Rosacea; Sarcoidosis; Sarcoidosis; Seborrheic dermatitis; Seborrheic keratosis; Staphylococcal scalded skin syndrome; Subcorneal pustular dermatitis; Systemic lupus erythematosus; Systemic lupus erythematosus (SLE); Toxic erythema; Vascular disorders of skin; Vasculitis limited to the skin, unspecified; Vitiligo; Xeroderma pigmentosum

hyperlipidemia Standard Concept: Hypercholesterolemia

Source Concepts: Hyperlipidemia, unspecified; Mixed hyperlipidemia; Mixed hyperlipidemia; Other and unspecified hyperlipidemia; Other hyperlipidemia; Other hyperlipidemia

type 1 diabetes Source Concepts: Pre-existing type 1 diabetes mellitus, in childbirth; Pre-existing type 1 diabetes mellitus, in pregnancy, first trimester; Pre-existing type 1 diabetes mellitus, in pregnancy, second trimester; Pre-existing type 1 diabetes mellitus, in pregnancy, third trimester; Pre-existing type 1 diabetes mellitus, in pregnancy, unspecified trimester; Pre-existing type 1 diabetes mellitus, in the puerperium; Type 1 diabetes mellitus; Type 1 diabetes mellitus with circulatory complications; Type 1 diabetes mellitus with diabetic amyotrophy; Type 1 diabetes mellitus with diabetic arthropathy; Type 1 diabetes mellitus with diabetic autonomic (poly)neuropathy; Type 1 diabetes mellitus with diabetic cataract; Type 1 diabetes mellitus with diabetic chronic kidney disease; Type 1 diabetes mellitus with diabetic dermatitis; Type 1 diabetes mellitus with diabetic macular edema, resolved following treatment; Type 1 diabetes mellitus with diabetic macular edema, resolved following treatment, bilateral; Type 1 diabetes mellitus with diabetic macular edema, resolved following treatment, right eye; Type 1 diabetes mellitus with diabetic mononeuropathy; Type 1 diabetes mellitus with diabetic nephropathy; Type 1 diabetes mellitus with diabetic neuropathic arthropathy; Type 1 diabetes mellitus with diabetic neuropathy, unspecified; Type 1 diabetes mellitus with diabetic peripheral angiopathy with gangrene; Type 1 diabetes mellitus with diabetic peripheral angiopathy without gangrene; Type 1 diabetes mellitus with diabetic polyneuropathy; Type 1 diabetes mellitus with foot ulcer; Type 1 diabetes mellitus with hyperglycemia; Type 1 diabetes mellitus with hypoglycemia; Type 1 diabetes mellitus with hypoglycemia with coma; Type

1 diabetes mellitus with hypoglycemia without coma; Type 1 diabetes mellitus with ketoacidosis; Type 1 diabetes mellitus with ketoacidosis with coma; Type 1 diabetes mellitus with ketoacidosis without coma; Type 1 diabetes mellitus with kidney complications; Type 1 diabetes mellitus with mild nonproliferative diabetic retinopathy; Type 1 diabetes mellitus with mild nonproliferative diabetic retinopathy with macular edema; Type 1 diabetes mellitus with mild nonproliferative diabetic retinopathy with macular edema, bilateral; Type 1 diabetes mellitus with mild nonproliferative diabetic retinopathy with macular edema, left eye; Type 1 diabetes mellitus with mild nonproliferative diabetic retinopathy with macular edema, right eye; Type 1 diabetes mellitus with mild nonproliferative diabetic retinopathy with macular edema, unspecified eye; Type 1 diabetes mellitus with mild nonproliferative diabetic retinopathy without macular edema; Type 1 diabetes mellitus with mild nonproliferative diabetic retinopathy without macular edema, bilateral; Type 1 diabetes mellitus with mild nonproliferative diabetic retinopathy without macular edema, left eye; Type 1 diabetes mellitus with mild nonproliferative diabetic retinopathy without macular edema, right eye; Type 1 diabetes mellitus with mild nonproliferative diabetic retinopathy without macular edema, unspecified eye; Type 1 diabetes mellitus with moderate nonproliferative diabetic retinopathy; Type 1 diabetes mellitus with moderate nonproliferative diabetic retinopathy with macular edema; Type 1 diabetes mellitus with moderate nonproliferative diabetic retinopathy with macular edema, bilateral; Type 1 diabetes mellitus with moderate nonproliferative diabetic retinopathy with macular edema, left eye; Type 1 diabetes mellitus with moderate nonproliferative diabetic retinopathy with macular edema, right eye; Type 1 diabetes mellitus with moderate nonproliferative diabetic retinopathy with macular edema, unspecified eye; Type 1 diabetes mellitus with moderate nonproliferative diabetic retinopathy without macular edema; Type 1 diabetes mellitus with moderate nonproliferative diabetic retinopathy without macular edema, bilateral; Type 1 diabetes mellitus with moderate nonproliferative diabetic retinopathy without macular edema, left eye; Type 1 diabetes mellitus with moderate nonproliferative diabetic retinopathy without macular edema, right eye; Type 1 diabetes mellitus with moderate nonproliferative diabetic retinopathy without macular edema, unspecified eye; Type 1 diabetes mellitus with neurological complications; Type 1 diabetes mellitus with ophthalmic complications; Type 1 diabetes mellitus with oral complications; Type 1 diabetes mellitus with other circulatory complications; Type 1 diabetes mellitus with other diabetic arthropathy; Type 1 diabetes mellitus with other diabetic kidney complication; Type 1 diabetes mellitus with other diabetic neurological complication; Type 1 diabetes mellitus with other diabetic ophthalmic complication; Type 1 diabetes mellitus with other oral complications; Type 1 diabetes mellitus with other skin complications; Type 1 diabetes mellitus with other skin ulcer; Type 1 diabetes mellitus with other specified complication; Type 1 diabetes mellitus with other specified complications; Type 1 diabetes mellitus with proliferative diabetic retinopathy; Type 1 diabetes mellitus with proliferative diabetic retinopathy with combined traction retinal detachment and rhegmatogenous retinal detachment; Type 1 diabetes mellitus with proliferative diabetic retinopathy with combined traction retinal detachment and rhegmatogenous retinal detachment, bilateral; Type 1 diabetes mellitus with proliferative diabetic retinopathy with combined traction retinal detachment and rhegmatogenous retinal detachment, left eye; Type 1 diabetes mellitus with proliferative diabetic retinopathy with combined traction retinal detachment and rhegmatogenous retinal detachment, right eye; Type 1 diabetes mellitus with proliferative diabetic retinopathy with macular edema; Type 1 diabetes mellitus with proliferative diabetic retinopathy with macular edema, bilateral; Type 1 diabetes mellitus with proliferative diabetic retinopathy with macular edema, left eye; Type 1 diabetes mellitus with proliferative diabetic retinopathy with macular edema, right eye; Type 1 diabetes mellitus with proliferative diabetic retinopathy with macular edema, unspecified eye; Type 1 diabetes mellitus with proliferative diabetic retinopathy with traction retinal detachment involving the macula; Type 1 diabetes mellitus with proliferative diabetic retinopathy with traction retinal detachment involving the macula, bilateral; Type 1 diabetes mellitus with proliferative diabetic retinopathy with traction retinal detachment involving the macula, left eye; Type 1 diabetes mellitus with proliferative diabetic retinopathy with traction retinal detachment involving the macula, right eye; Type 1 diabetes mellitus with proliferative diabetic retinopathy with traction retinal detachment not involving the macula; Type 1 diabetes mellitus with proliferative diabetic retinopathy with traction retinal de-

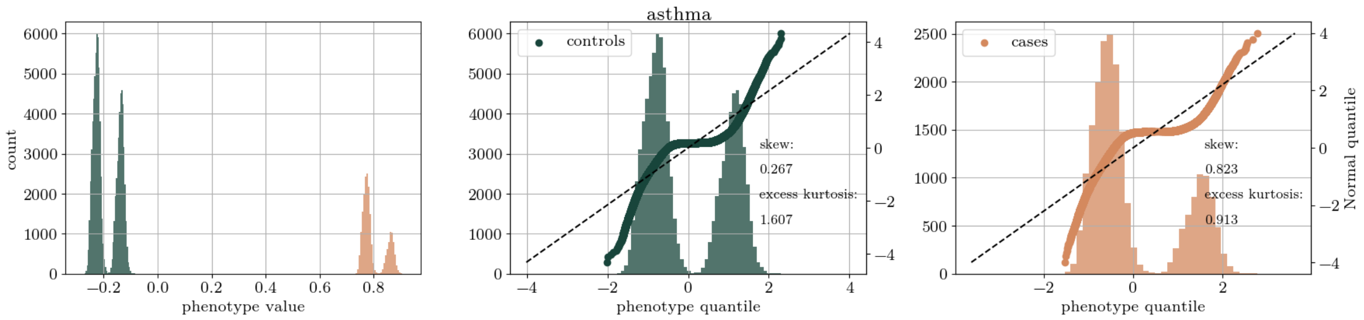

Supplemental Figure 13: Residual distribution of the AoU asthma phenotype.

tachment not involving the macula, bilateral; Type 1 diabetes mellitus with proliferative diabetic retinopathy with traction retinal detachment not involving the macula, left eye; Type 1 diabetes mellitus with proliferative diabetic retinopathy with traction retinal detachment not involving the macula, right eye; Type 1 diabetes mellitus with proliferative diabetic retinopathy without macular edema; Type 1 diabetes mellitus with proliferative diabetic retinopathy without macular edema, bilateral; Type 1 diabetes mellitus with proliferative diabetic retinopathy without macular edema, left eye; Type 1 diabetes mellitus with proliferative diabetic retinopathy without macular edema, right eye; Type 1 diabetes mellitus with proliferative diabetic retinopathy without macular edema, unspecified eye; Type 1 diabetes mellitus with severe nonproliferative diabetic retinopathy; Type 1 diabetes mellitus with severe nonproliferative diabetic retinopathy with macular edema; Type 1 diabetes mellitus with severe nonproliferative diabetic retinopathy with macular edema, bilateral; Type 1 diabetes mellitus with severe nonproliferative diabetic retinopathy with macular edema, left eye; Type 1 diabetes mellitus with severe nonproliferative diabetic retinopathy with macular edema, right eye; Type 1 diabetes mellitus with severe nonproliferative diabetic retinopathy with macular edema, unspecified eye; Type 1 diabetes mellitus with severe nonproliferative diabetic retinopathy without macular edema; Type 1 diabetes mellitus with severe nonproliferative diabetic retinopathy without macular edema, bilateral; Type 1 diabetes mellitus with severe nonproliferative diabetic retinopathy without macular edema, left eye; Type 1 diabetes mellitus with severe nonproliferative diabetic retinopathy without macular edema, right eye; Type 1 diabetes mellitus with severe nonproliferative diabetic retinopathy without macular edema, unspecified eye; Type 1 diabetes mellitus with skin complications; Type 1 diabetes mellitus with stable proliferative diabetic retinopathy; Type 1 diabetes mellitus with stable proliferative diabetic retinopathy, bilateral; Type 1 diabetes mellitus with stable proliferative diabetic retinopathy, left eye; Type 1 diabetes mellitus with stable proliferative diabetic retinopathy, right eye; Type 1 diabetes mellitus with stable proliferative diabetic retinopathy, unspecified eye; Type 1 diabetes mellitus with unspecified complications; Type 1 diabetes mellitus with unspecified diabetic retinopathy; Type 1 diabetes mellitus with unspecified diabetic retinopathy with macular edema; Type 1 diabetes mellitus with unspecified diabetic retinopathy without macular edema; Type 1 diabetes mellitus without complications

hypertension Source Concepts: Benign essential hypertension; Essential (primary) hypertension; Essential hypertension; Unspecified essential hypertension

height Body height

bmi field ID: Body mass index; Body mass index (BMI) [Ratio]

total bilirubin Bilirubin.total [Mass/volume] in Serum or Plasma

hdl Cholesterol in HDL [Mass/volume] in Serum or Plasma

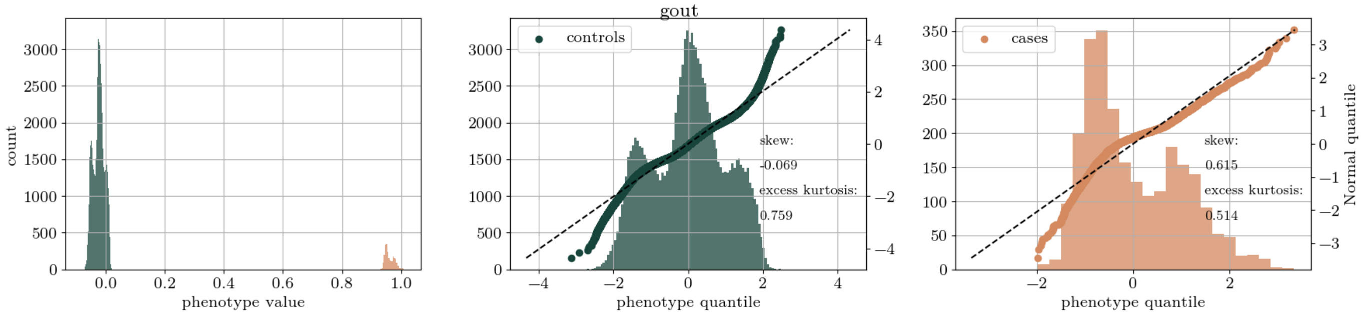

Supplemental Figure 14: Residual distribution of the AoU gout phenotype.

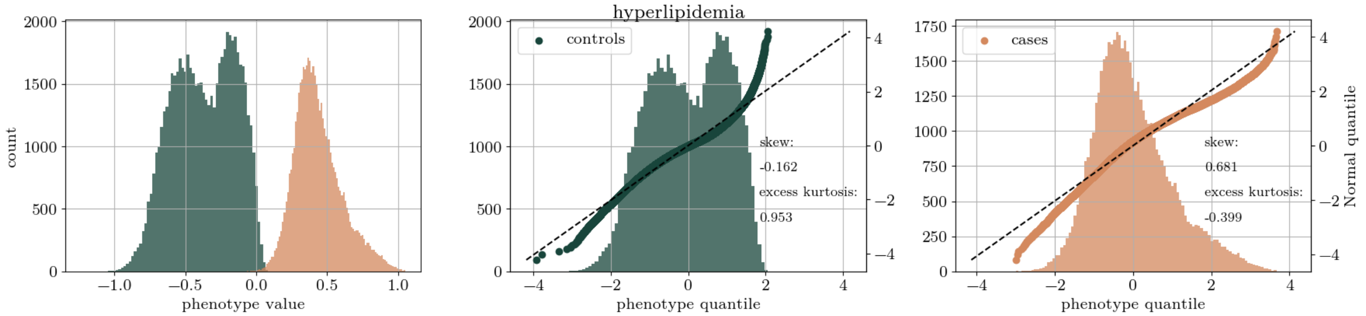

Supplemental Figure 15: Residual distribution of the AoU hyperlipidemia phenotype.

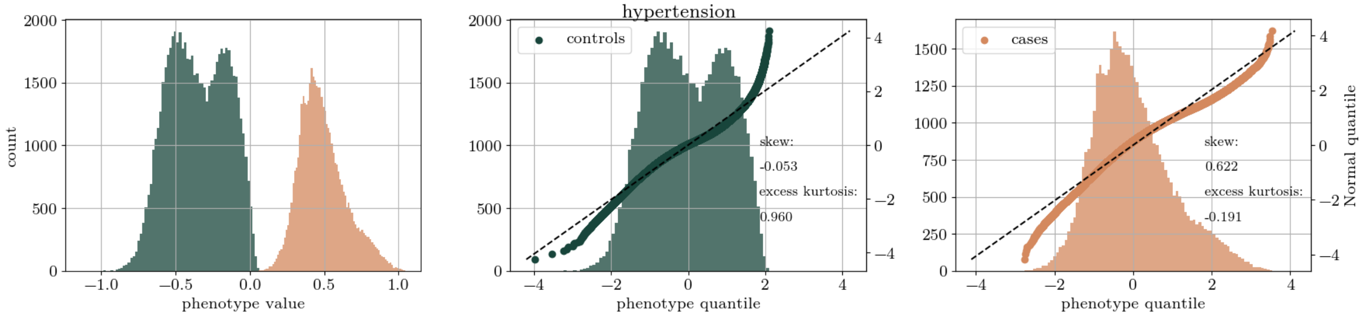

Supplemental Figure 16: Residual distribution of the AoU hypertension phenotype.

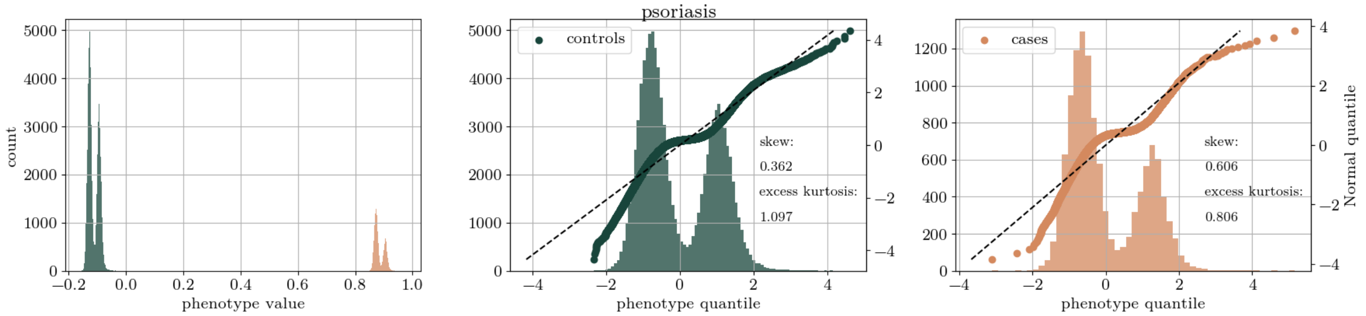

Supplemental Figure 17: Residual distribution of the AoU psoriasis phenotype.

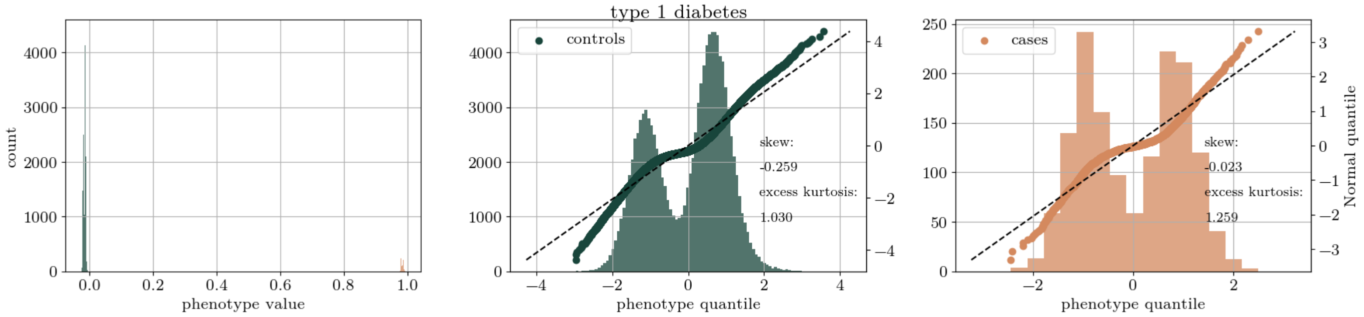

Supplemental Figure 18: Residual distribution of the AoU type 1 diabetes phenotype.

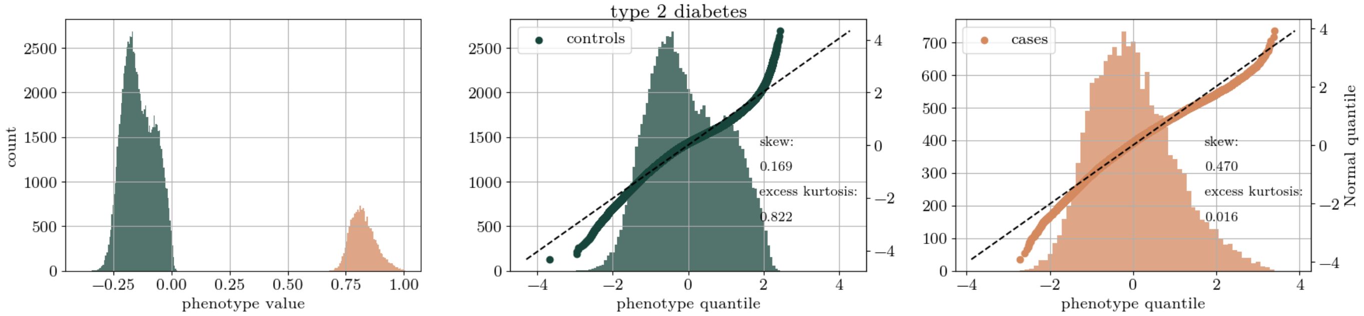

Supplemental Figure 19: Residual distribution of the AoU type 2 diabetes phenotype.

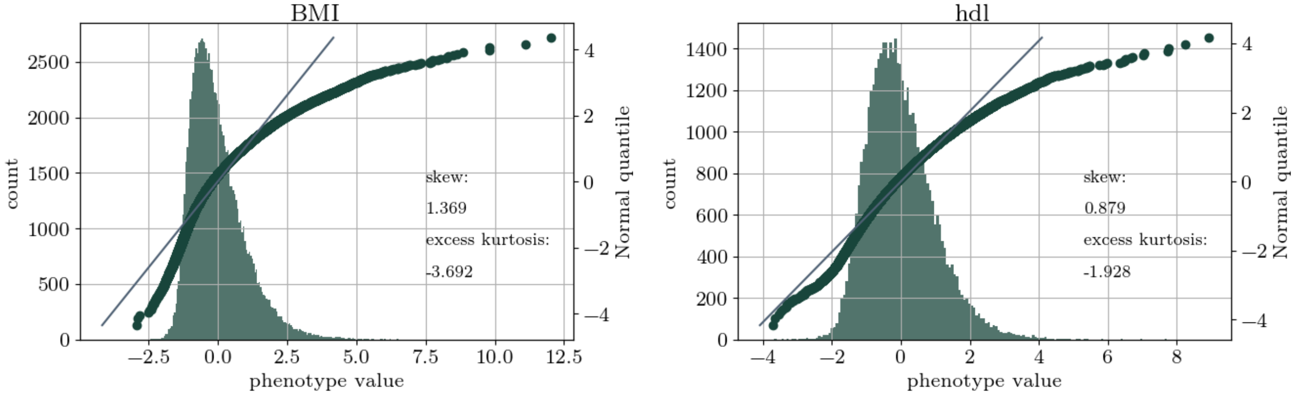

Supplemental Figure 20: Residual distribution of the AoU bmi and hdl phenotypes.

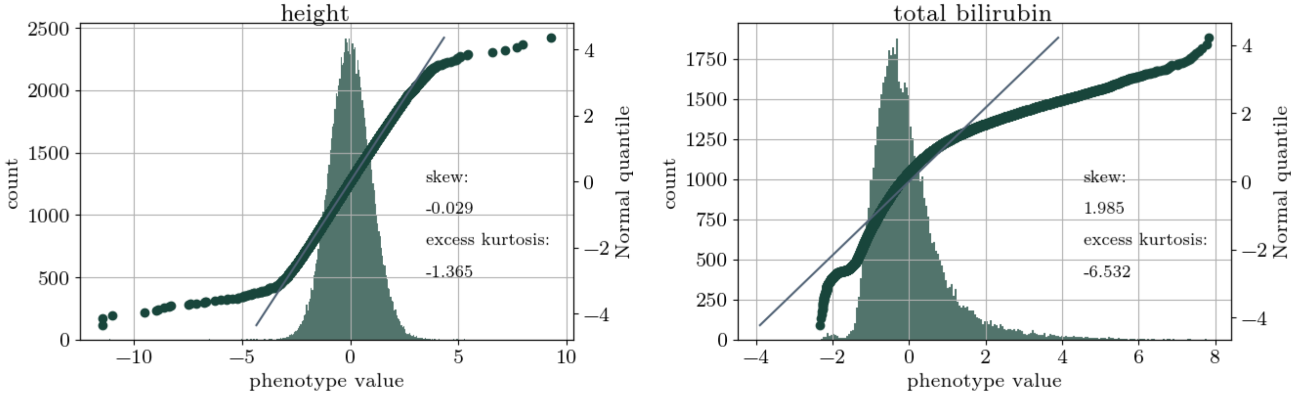

Supplemental Figure 21: Residual distribution of the AoU height and total bilirubin phenotypes.

2 Polygenic score computation

Here we provide additional details, including mathematical descriptions, of how to generate the polygenic scores described in the main text.

In step (2) we build residual phenotypes. For a raw phenotype, $\vec{y}$, We regress

$$\vec{y} \sim \vec{\theta} \cdot \bar{K} \rightarrow \vec{\theta}^{*}\,, \tag{2.1}$$

for covariate matrix $\bar{K}$ and coefficients $\vec{\theta}$. Residual phenotypes, $\vec{y}'$ are then built by subtracting off the covariate contribution from the raw (or sex specific z-scored) phenotype

$$\vec{y}' = \vec{y} - \vec{y}^{*} \qquad \text{where} \qquad \vec{y}^{*} = \vec{\theta}^{*} \cdot \bar{K}\,. \tag{2.2}$$

In step (4) we use Scikit-Learn to train predictors. This is a straightforward application of using coordinate descent to minimize the LASSO objective function,

$$\mathcal{O}(\lambda) = \frac{1}{2N}||\vec{y}' - \bar{X} \cdot \vec{\beta}||^2_{L_2} + \lambda|\vec{\beta}|_{L_1}\,, \tag{2.3}$$

for features across the entire autosome or on the blocks (chromosome). Here $\bar{X}$ is the encoded genotype matrix, N is the number of samples (i.e., the length of $\vec{y}$), and $\vec{\beta}$ are the SNV weights. As detailed in the associated code examples, we used the Scikit-Learn function `linear_model.lasso_path` with 100-200 steps, $0.0005 < \lambda_{min}/\lambda_{max} < 0.001$, and let it run for a maximum of 1500 iterations. Examples of the validation paths for block vs global LASSO in the UKB can be found in the section 7. Model selection, $\lambda \rightarrow \lambda^{*}$, is done by selecting the maximal performance in the validation/model-selection set.

In step (5) we perform the block regression. We index each block by the label b. For each block we have a genotype matrix, X^b_{ij}, where the i indexes the samples and j the features. Unweighted scores for each block can be written as: $\sum_j \bar{X}^b_{ij} \cdot \beta^b_j$. For a phenotype, y_i, we determine the individual block weights, α_b, via linear regression

$$y_j \sim \sum_b \alpha_b \times \sum_i \bar{X}^b_{ij} \cdot \beta^b_j \rightarrow \alpha^{*}\,. \tag{2.4}$$

The final step (6) involves constructing the full model by applying

$$\mathcal{B}^b_j = \alpha_b \cdot \beta^b_j \tag{2.5}$$

to genotypes.

Case control conditions are evaluated using area under the receiver operator curve (AUC) using the PGS and case-control status. Continuous phenotypes are evaluated using the correlation between the PGS and the adjusted phenotype.

Covariate adjustment: to keep the pipeline as simple and uniform as possible we chose use linear regression for all covariate contributions. It is common for dichotomous phenotypes, e.g., case control conditions, to use un-penalized logistic regression for the covariates instead. In **Supplemental Table 3** we show that for the approach described in this work it leads to small differences in the evaluation metrics of the final predictors. We also show the difference in effect distributions between covariate linear and logistic regression in **Supplemental Figure 22-Supplemental Figure 25**.

trait	linear regression	logistic regression
asthma	$0.623_{0.005}$	$0.629_{0.005}$
gout	$0.65_{0.01}$	$0.66_{0.01}$
hyperlipidemia	$0.660_{0.003}$	$0.659_{0.003}$
hypertension	$0.633_{0.003}$	$0.628_{0.003}$
psoriasis	$0.68_{0.01}$	$0.68_{0.01}$
type 1 diabetes	$0.67_{0.02}$	$0.67_{0.02}$
type 2 diabetes	$0.635_{0.007}$	$0.654_{0.006}$

Supplemental Table 3: Comparison of genetics only AUCs using the global LASSO for predictors trained on a residual phenotype from linearly regressed covariates (center column) vs logistically regressed covariates (right column). Color coding indicates the same full agreement and partial agreement from the main text.

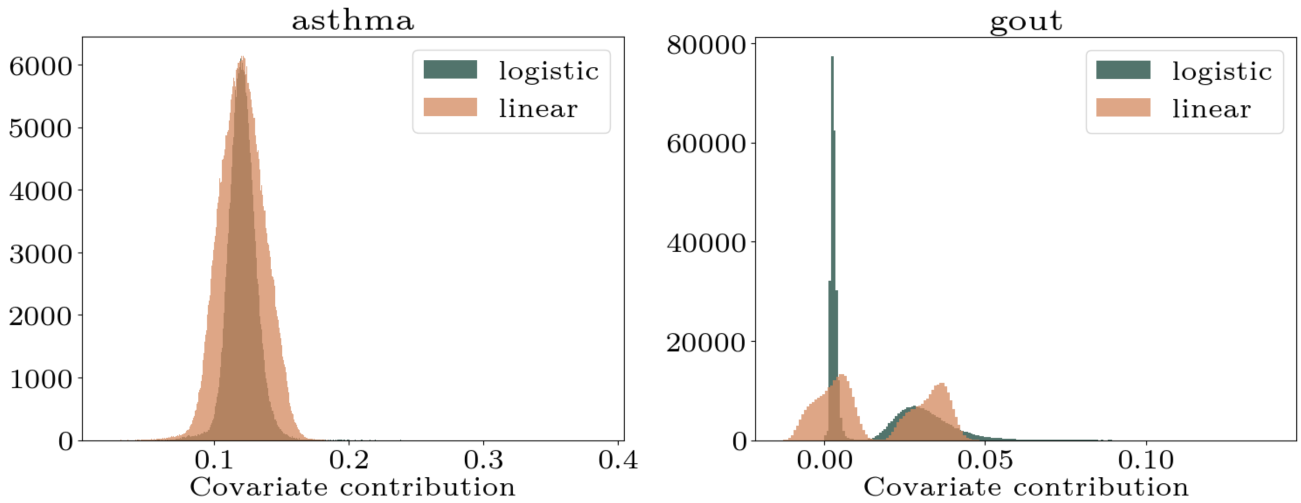

Supplemental Figure 22: Comparison distributions of covariate effects from linear vs logistic regression as applied to a training set for asthma and gout.

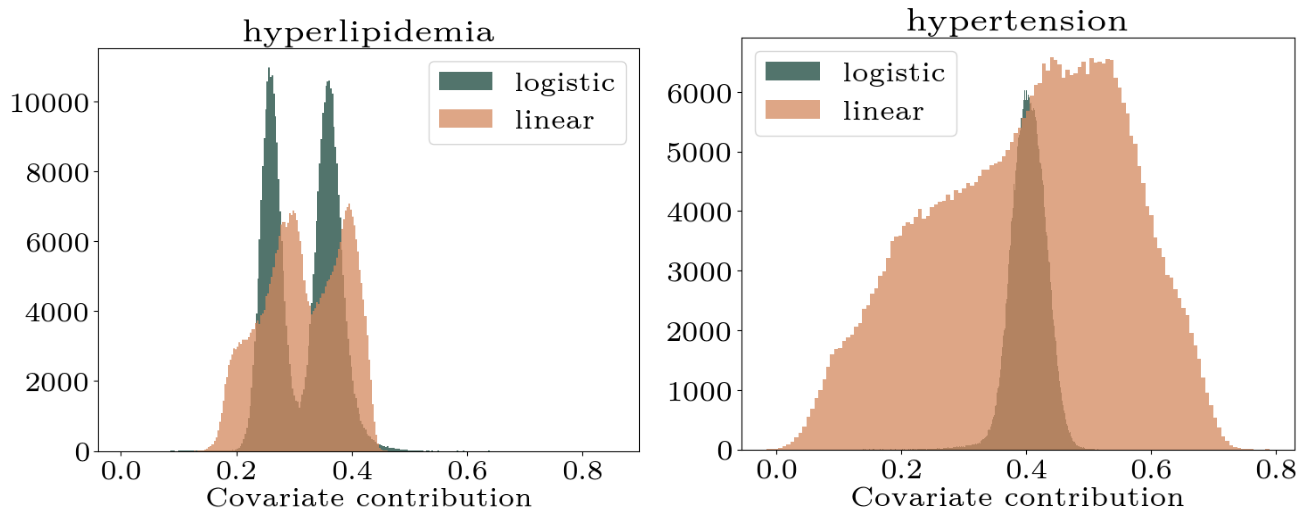

Supplemental Figure 23: Comparison distributions of covariate effects from linear vs logistic regression as applied to a training set for hyperlipidemia and hypertension.

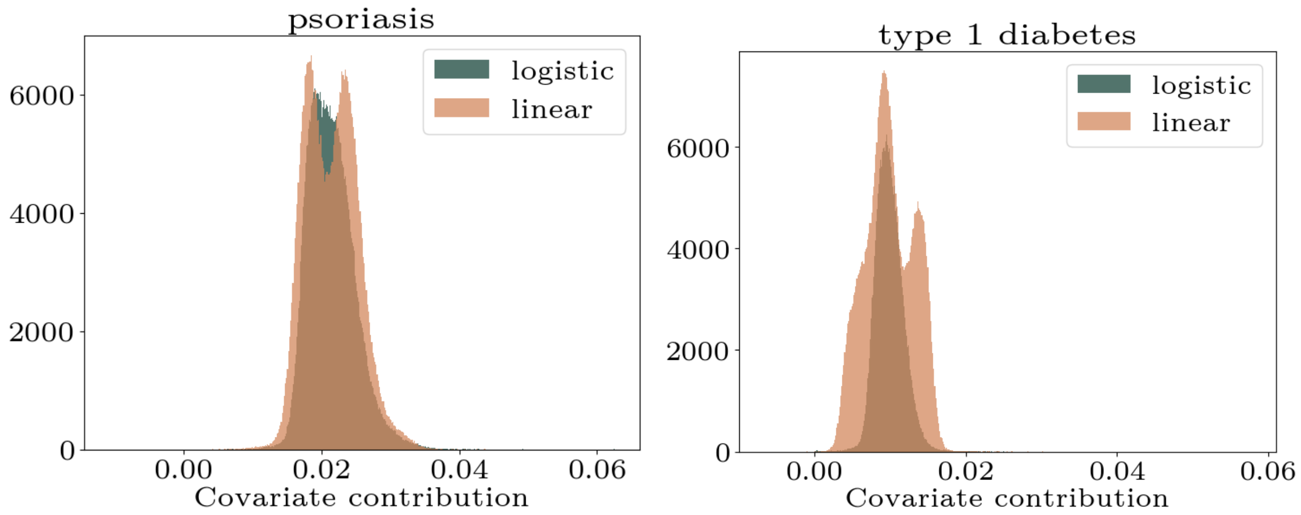

Supplemental Figure 24: Comparison distributions of covariate effects from linear vs logistic regression as applied to a training set for psoriasis and type 1 diabetes.

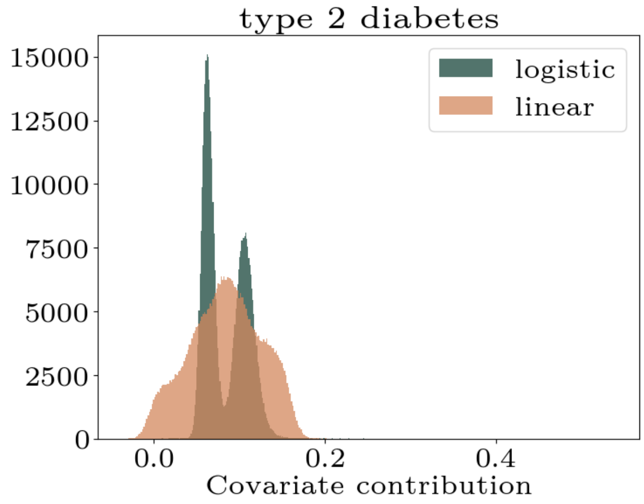

Supplemental Figure 25: Comparison distributions of covariate effects from linear vs logistic regression as applied to a training set for type 2 diabetes.

3 Simulations and Benchmarking

As described in the main text, simulation data was generated with HaploDynamics[1]. Code used to analyze the simulated data can be found in the Hsu group GitHub: `https://github.com/MSU-Hsu-Lab/blockLASSO`. Because the files are large (∼350 MB), the simulated genetic data is stored on the MSU OneDrive in the "Hsu Lab" space and can be shared via email request.

A comparison between our Python based methods and the state-of-the-art R based methods in the packages bigstatsr/bigsnpr[2, 3] were done for LASSO calculations for standing height. The R analysis primarily uses the bigstatsr PLR function `big_spLinReg()`. We generated new training and testing sets for this comparison hence the slight difference between what is here and what is presented in Table 1. We ran both R and Python pipelines on the exact same training and testing sets. We were able to use the bigstatsr package to execute a LASSO optimization on large set of all called 645k UKB variants which which passed basic quality controls: missing genotype rate cut-off 3%, minor allele frequency cut-off 0.1%, and missing individual rate cut-off 3%.

The main benefit of the R packages over the Python libraries is that the R calculations work on file-backed matrices where the data is cached on disk as opposed to loaded into RAM. This introduces more disk space requirements but drastically reduces the RAM requirements. For example, on the LASSO example for height, we were able to use 645,568 variants (those passing our basic quality control and filtering) as covariates - whereas the top ranked GWAS method limits the number of variants to what can be loaded into memory. For the bigstatsr run, we used 2 cross validation folds with multithreading up to 19 cores and this calculation required only 4.14 GB of RAM. We did not set `dfmax` nor `n.abort` in order to run bigstatsr as similar to our algorithm as possible, i.e. without selecting a maximum number of non-zero variables and without a strict stopping criterion based on the worsening of the validation set. As described in supplemental section 7, there are instances, e.g. when training on admixed populations or transporting PGS from one ancestry to another, where researchers could be interested in large training paths. This method took approximately 36 hours to run where at least 20 hours were spent generating the file-backed matrix required to pass into the LASSO subroutine - ie, subsetted to the desired training samples and replacing missing values. This replacement of missing values via imputation can require re-running when other training sets and features are needed. Ultimately this file-backed matrix required 250GB of disk space and a comparable amount of additional scratch space.

The predictor generated from this process had 87,737 non zero weights - about ∼13.6% of the total possible variants as input. This fraction of nonzero input covariates is comparable to the methods which use the top GWAS values filters. The PGS generated by the 645k bigsnpr predictor correlates with height at 0.645 whereas predictors built using the top 50k GWAS filter correlates with height at 0.619. From this, it suggests that using all variants compared to the top 50k GWAS leads to a roughly 5% performance gain - which is nontrivial when trying to optimize for the best possible prediction.

Additionally, for a direct comparison of the two pipelines we first did naive gwas p-value filtering to pre-select features. With top 10k SNPs by gwas rank we found corr = $0.536_{0.004}$ which ran in 1:48:52 (hrs:min:secs) and used ∼49GB of RAM. With the top 50k SNPs by gwas rank we found corr = $0.619_{0.004}$ which ran in 8:39:21 (hrs:min:secs) and used ∼237GB of RAM. In comparison, our global LASSO with the top 50k snps by GWAS rank finds corr = $0.620_{0.004}$ in ∼8 hours and requires ∼500 GB of RAM. Our blockLASSO with the equivalent of the top 50K SNPs ranked by gwas found corr = $0.595_{0.004}$ in 0:01:27 and required ∼23 GB of RAM.

Exact solution of the LASSO problem rely on some combination of hard disk space, RAM, and computing power and time. Naive approaches like the scikit-learn Python implementation have a large RAM requirement, but only require a single version of the raw genotypes. The R bigstatsr PLR approach is the most RAM efficient full solution, but requires large amounts of disk space to run for different trainings. The block approach advocated here still runs with meaningfully less memory and in meaningfully less time. We find these results indicate that the block approach can be useful for exploratory methods work.

4 PGS metrics plots

More comparisons of block vs global LASSO in AoU and UKB* (UKB reduced to training/testing sets comparable to AoU) can be seen in **Supplemental Figure 26 - Supplemental Figure 28**. Uncertainties reflect one standard deviation computed from 5-fold cross-validation and computing AUC/correlation with finite sample sizes (effects are added in quadrature). For the "global AoU" measurement only one training fold was run so the uncertainty is the larger of the finite size effect, or the corresponding uncertainty found in the UKB.

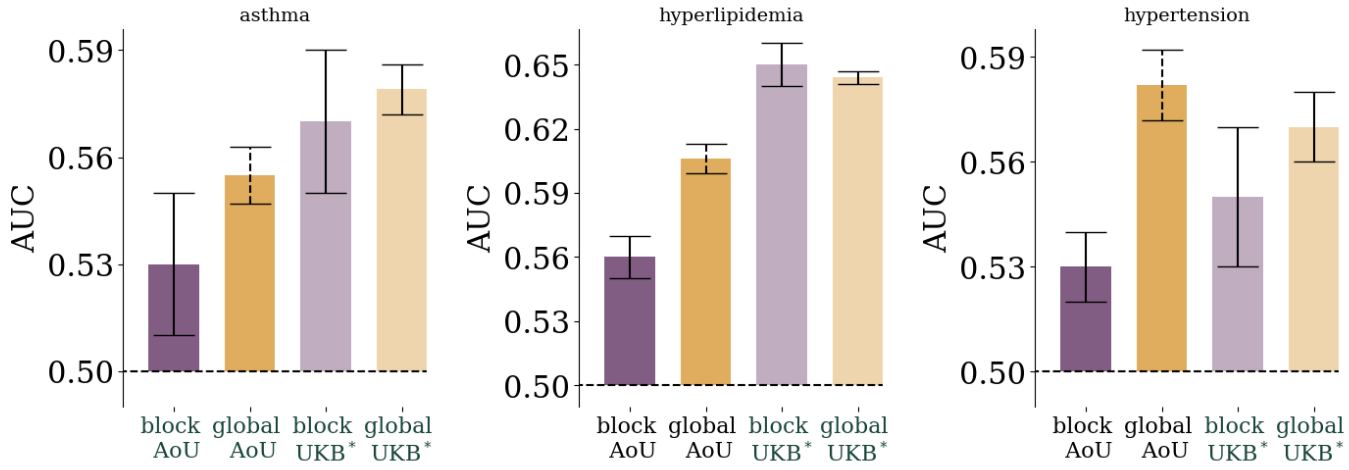

Supplemental Figure 26: Block vs global lasso performance in AoU and UKB* for asthma, hyperlipidemia, and hypertension.

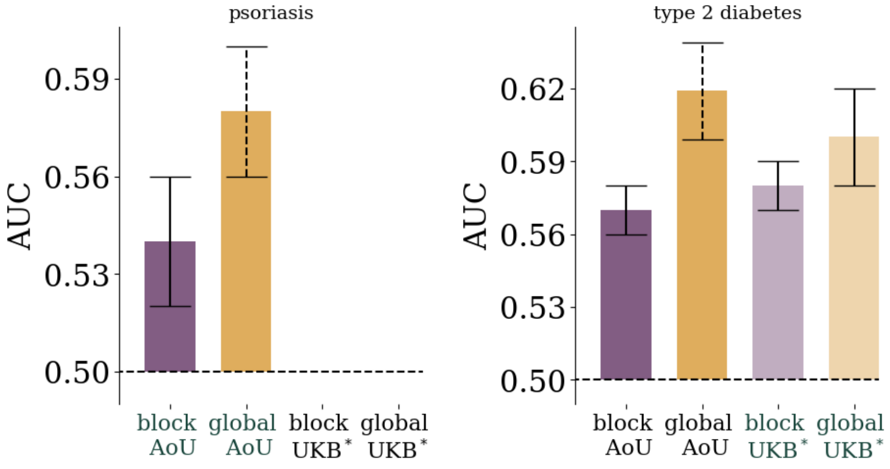

Supplemental Figure 27: Block vs global lasso performance in AoU and UKB* for psoriasis and type 2 diabetes. Because there are more cases in AoU than the UKB, a UKB* set was not trained for psoriasis.

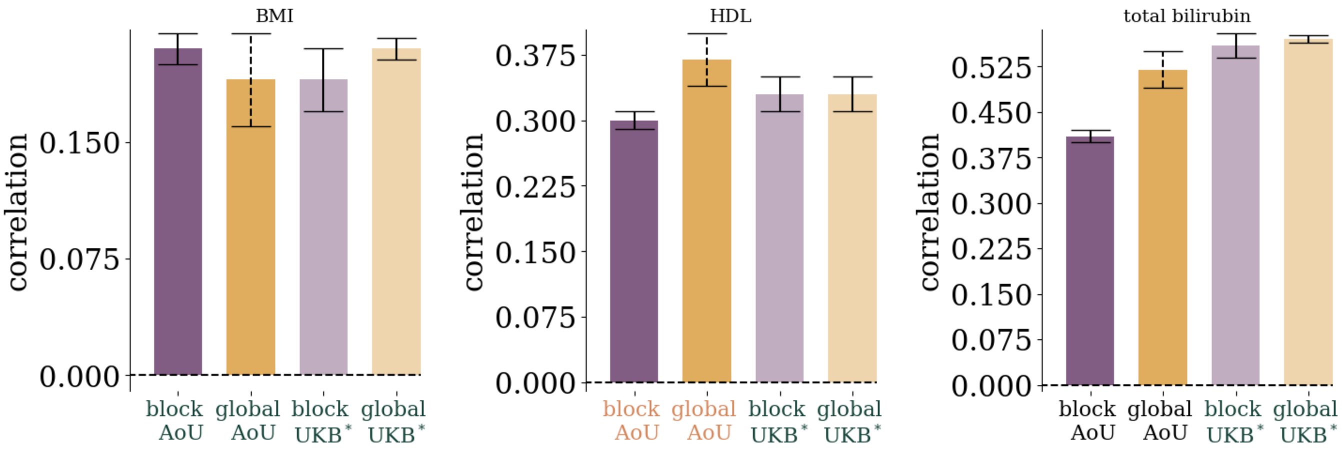

Supplemental Figure 28: Block vs global lasso performance in AoU and UKB* for bmi, hdl, and total bilirubin.

5 Variance plots

Here we report the additional variance explained per approximate base position and per chromosome, **Supplemental Figure 29 - Supplemental Figure 38**, for the phenotypes not displayed in the main text. The variance per location is normalized by the total variance explained by the PGS. The variance per location is filtered to only include contributions of at least 0.01% of the total PGS varaince. To make the variance per base pair position human readable we use 1 megabase pair sized bins.

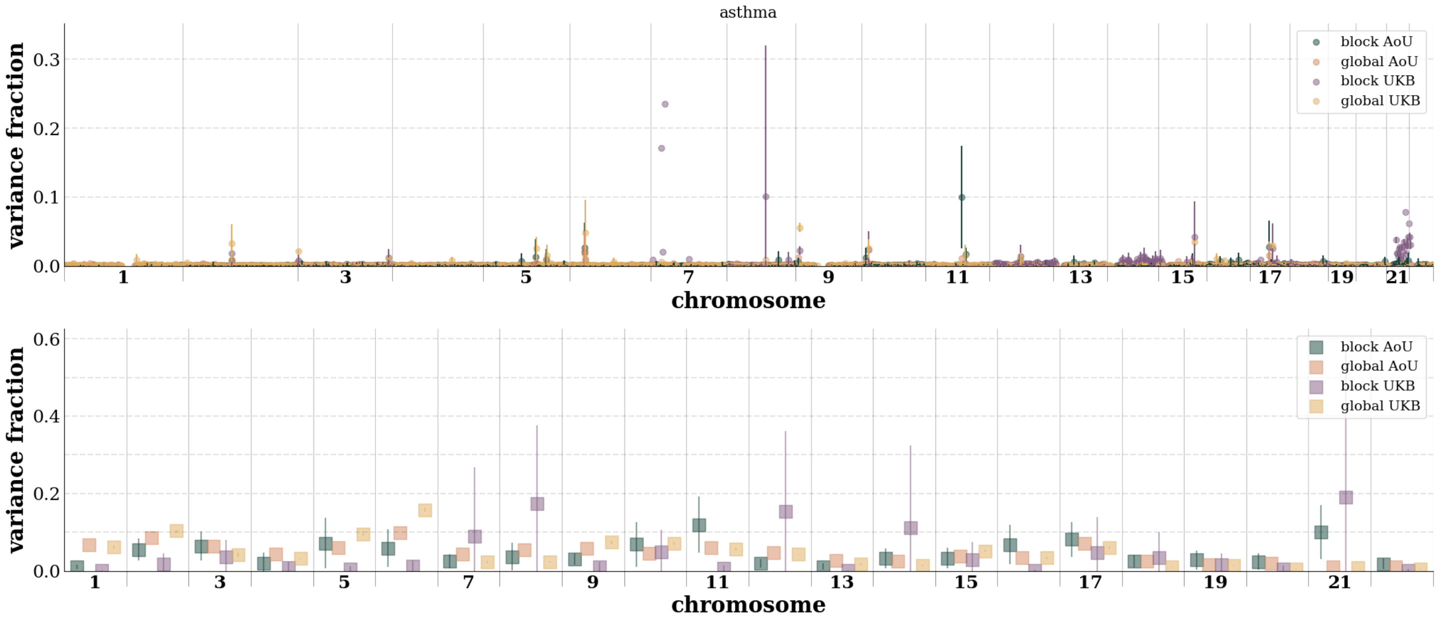

Supplemental Figure 29: Fraction of variance explained per binned base pair position (top) and per chromosome (bottom) in AoU and the UKB for asthma.

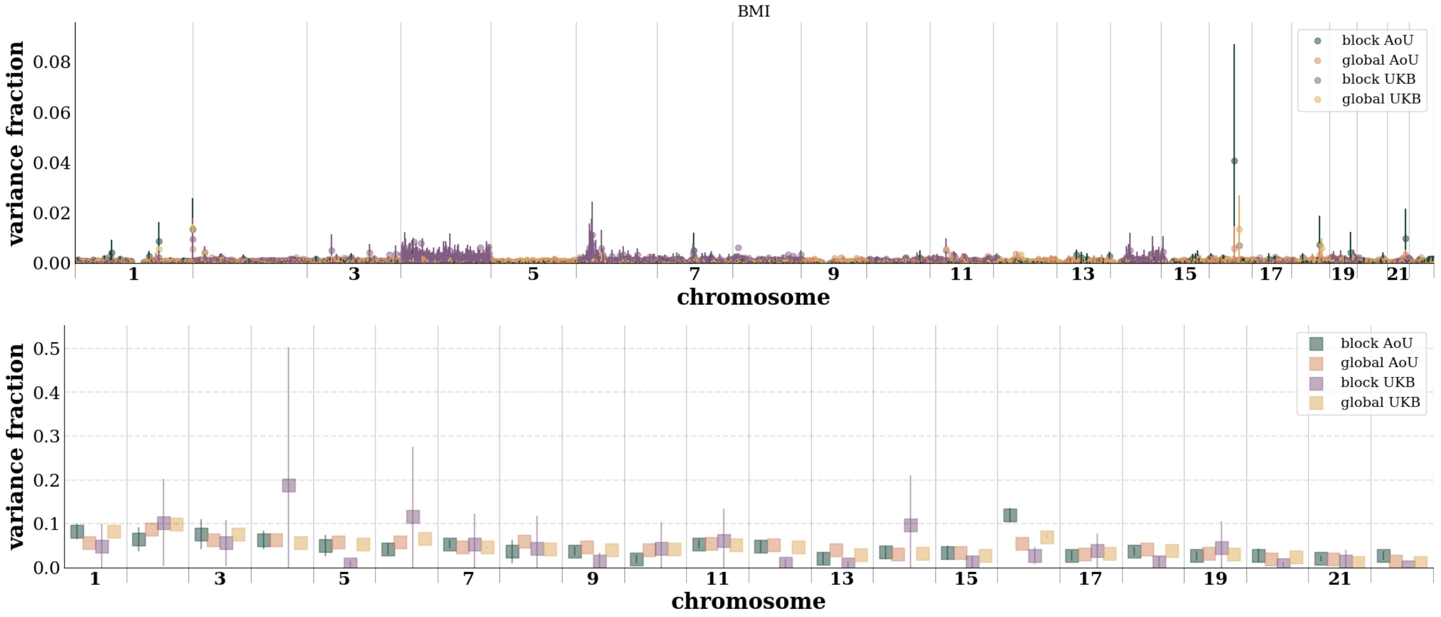

Supplemental Figure 30: Fraction of variance explained per binned base pair position (top) and per chromosome (bottom) in AoU and the UKB for bmi.

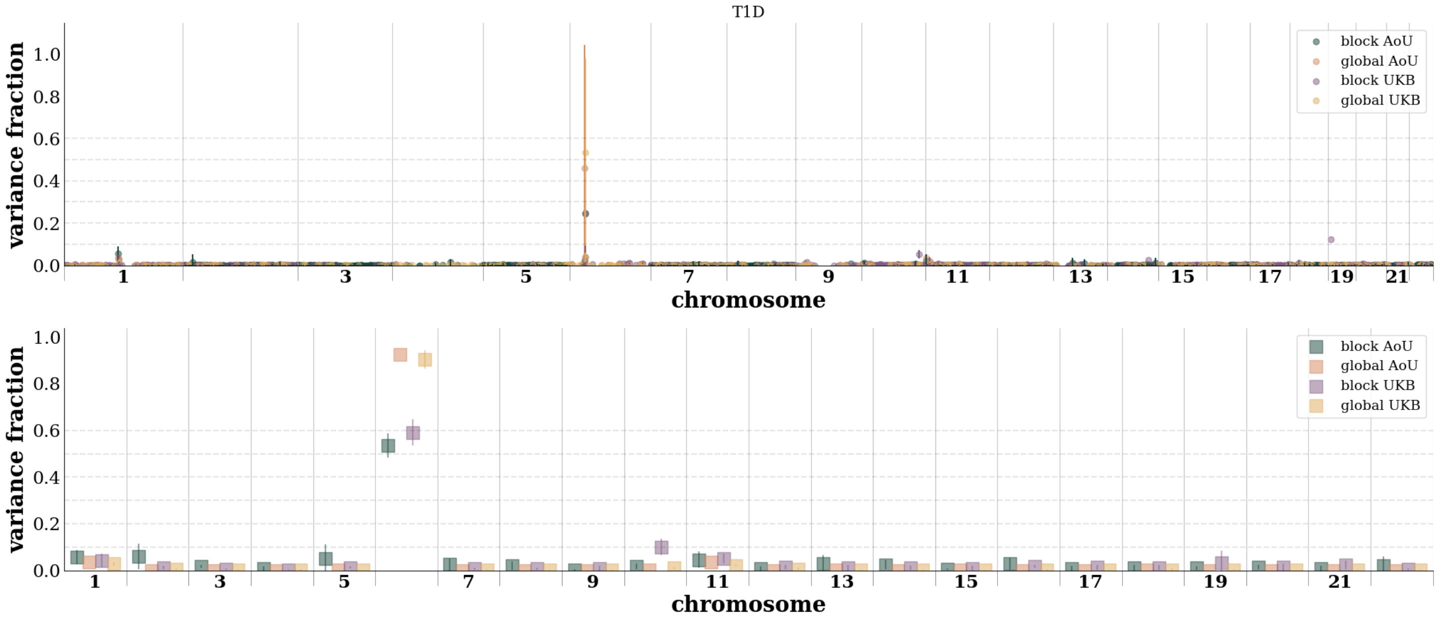

Supplemental Figure 31: Fraction of variance explained per binned base pair position (top) and per chromosome (bottom) in AoU and the UKB for type 1 diabetes.

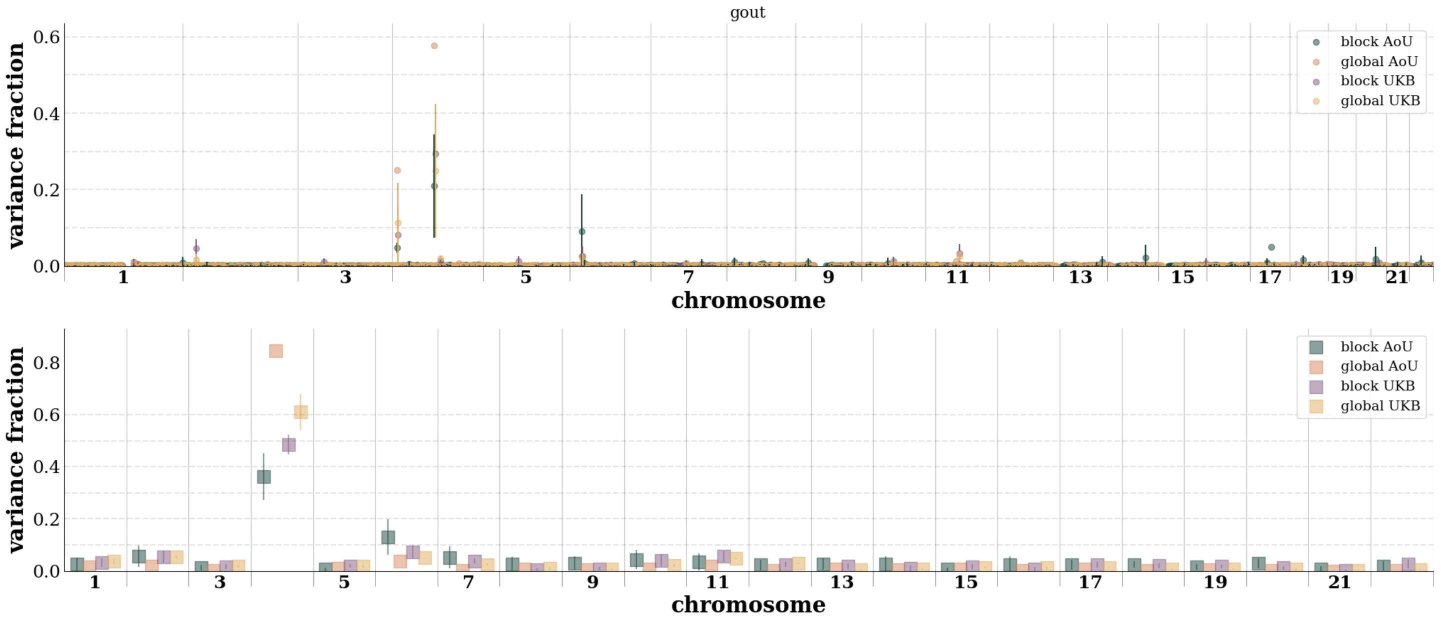

Supplemental Figure 32: Fraction of variance explained per binned base pair position (top) and per chromosome (bottom) in AoU and the UKB for gout.

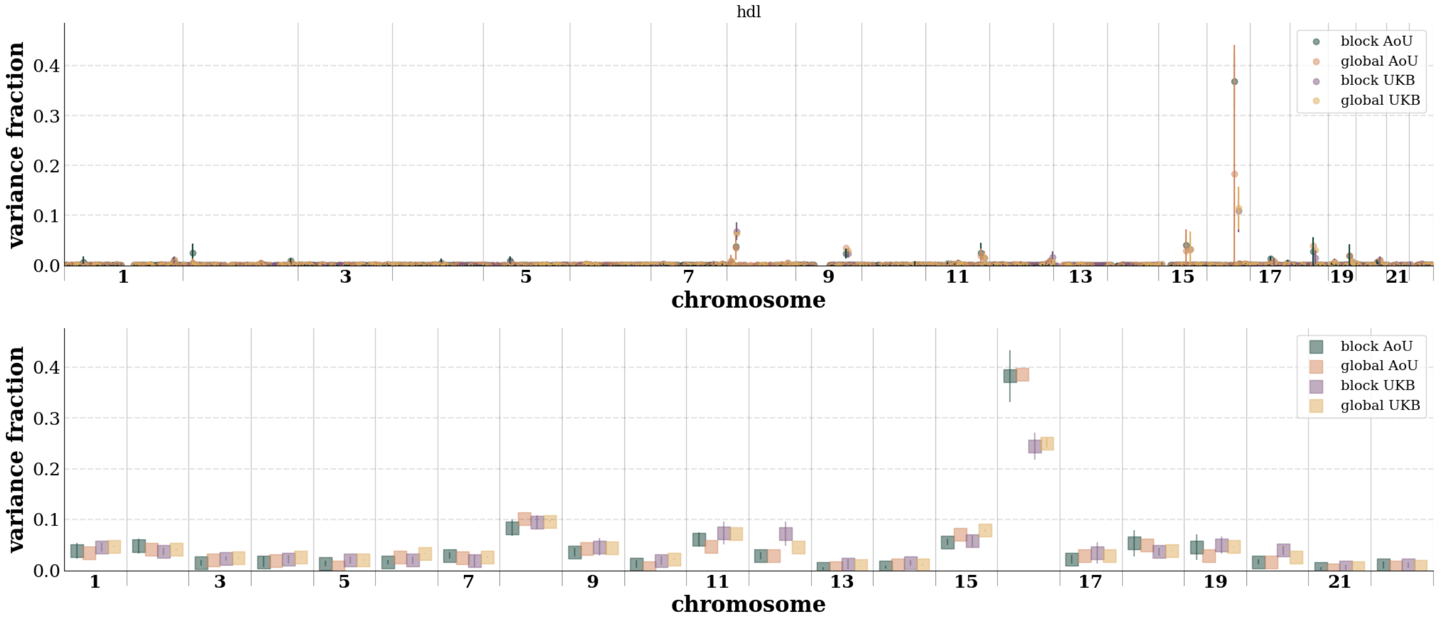

Supplemental Figure 33: Fraction of variance explained per binned base pair position (top) and per chromosome (bottom) in AoU and the UKB for hdl.

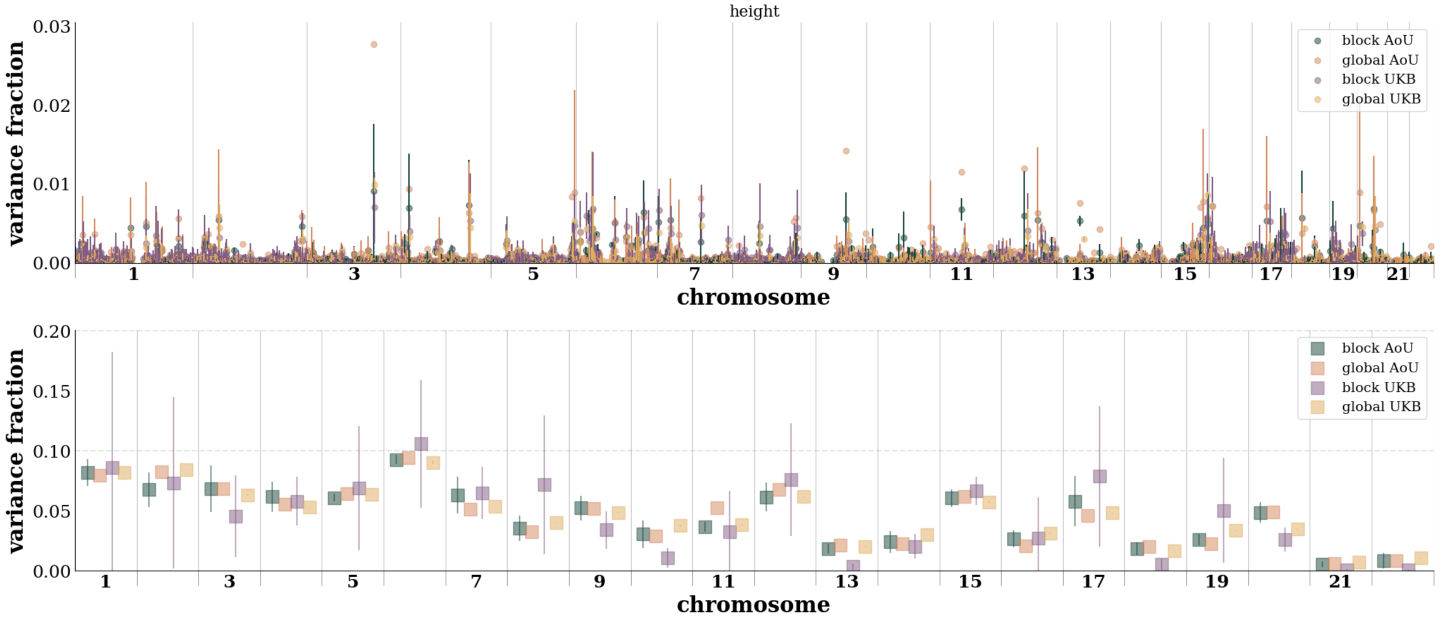

Supplemental Figure 34: Fraction of variance explained per binned base pair position (top) and per chromosome (bottom) in AoU and the UKB for height.

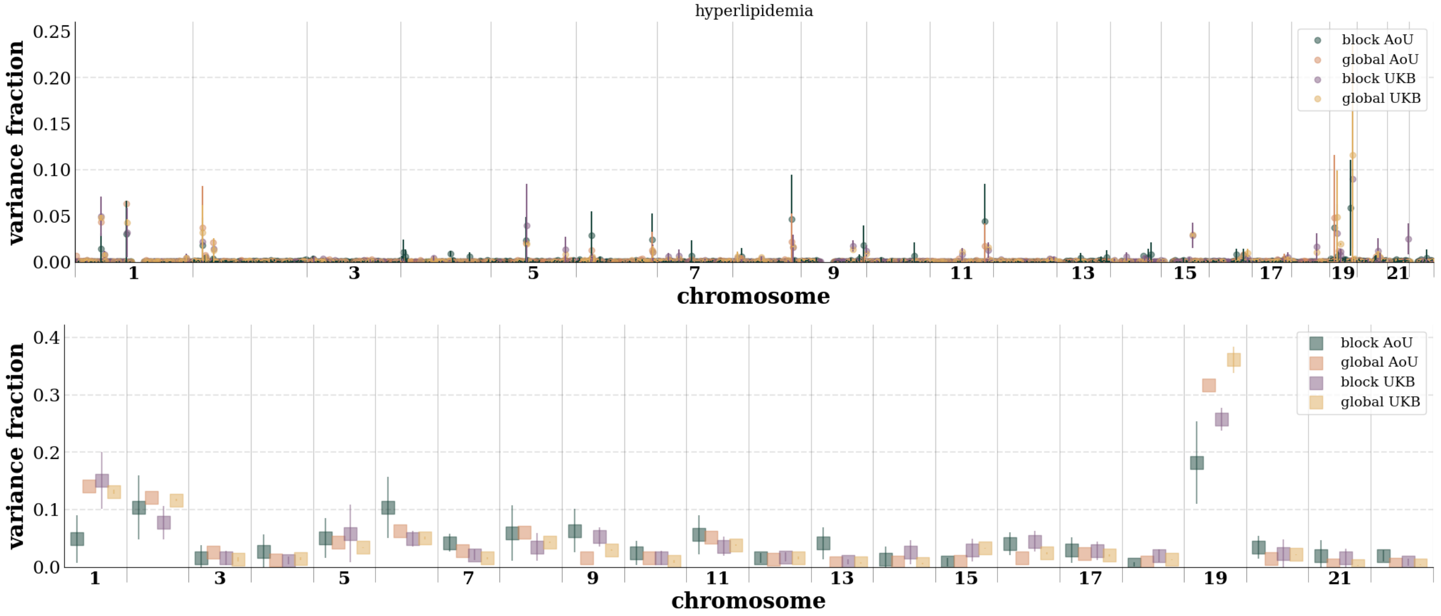

Supplemental Figure 35: Fraction of variance explained per binned base pair position (top) and per chromosome (bottom) in AoU and the UKB for hyperlipidemia.

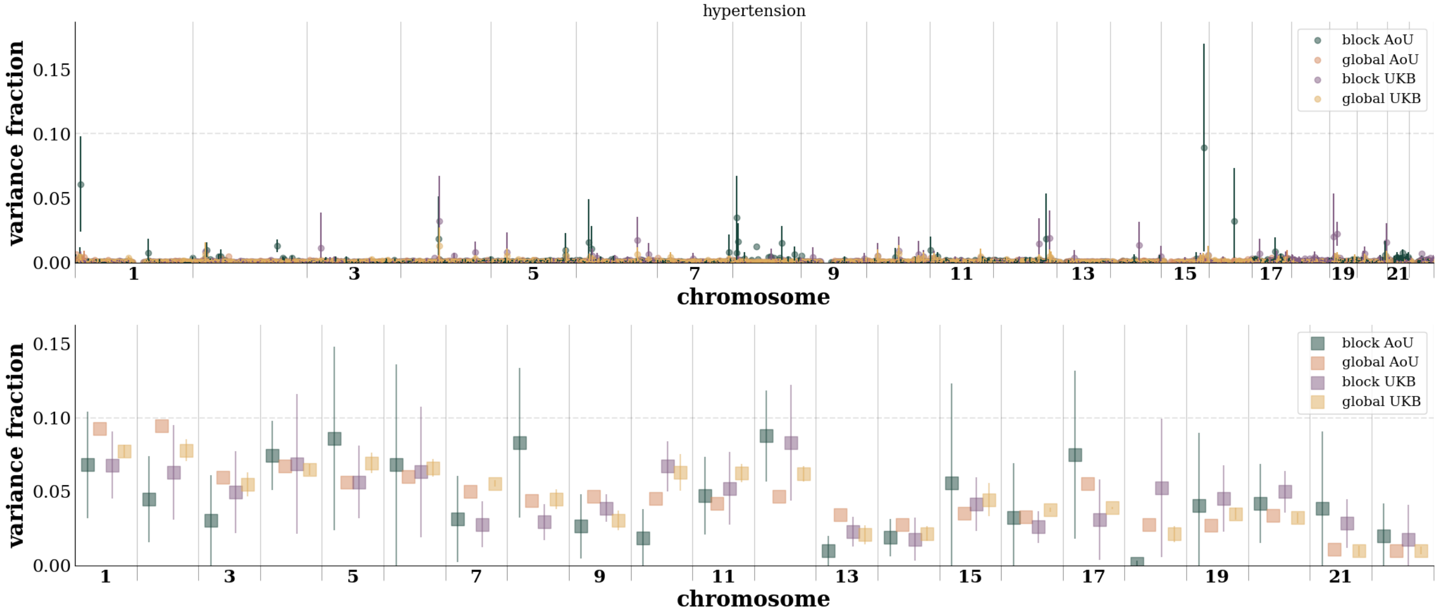

Supplemental Figure 36: Fraction of variance explained per binned base pair position (top) and per chromosome (bottom) in AoU and the UKB for hypertension.

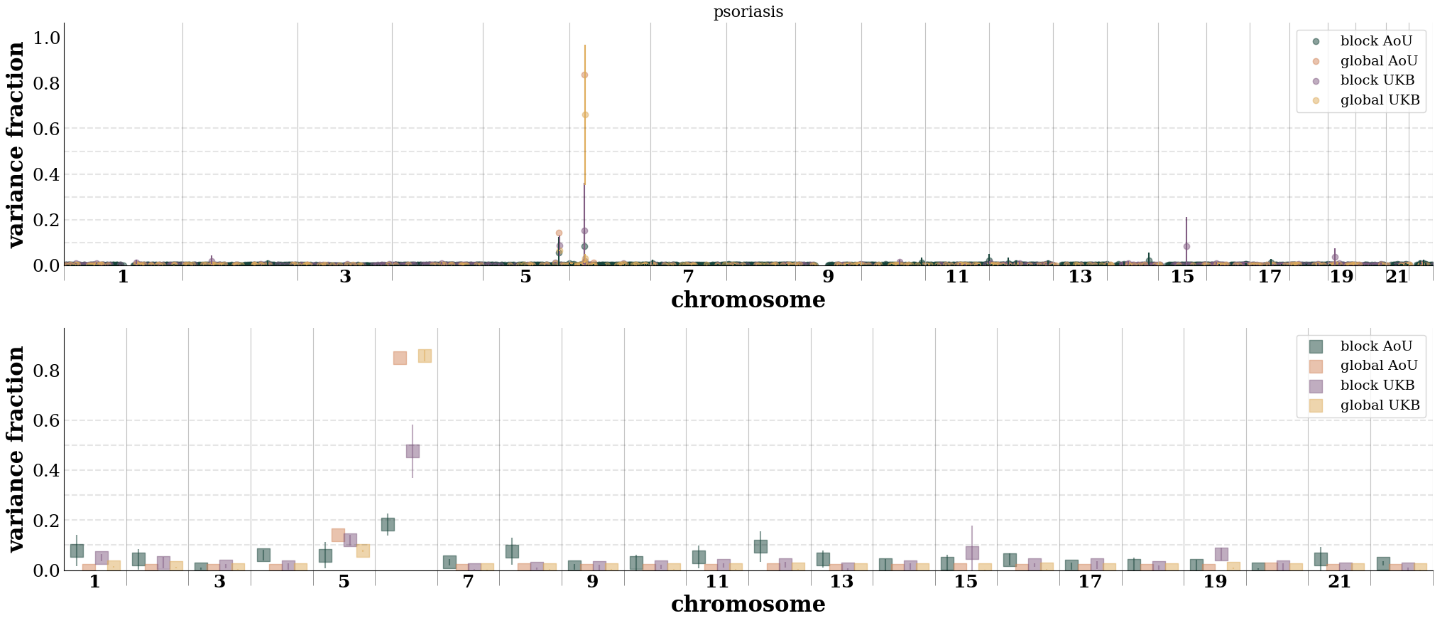

Supplemental Figure 37: Fraction of variance explained per binned base pair position (top) and per chromosome (bottom) in AoU and the UKB for psoriasis.

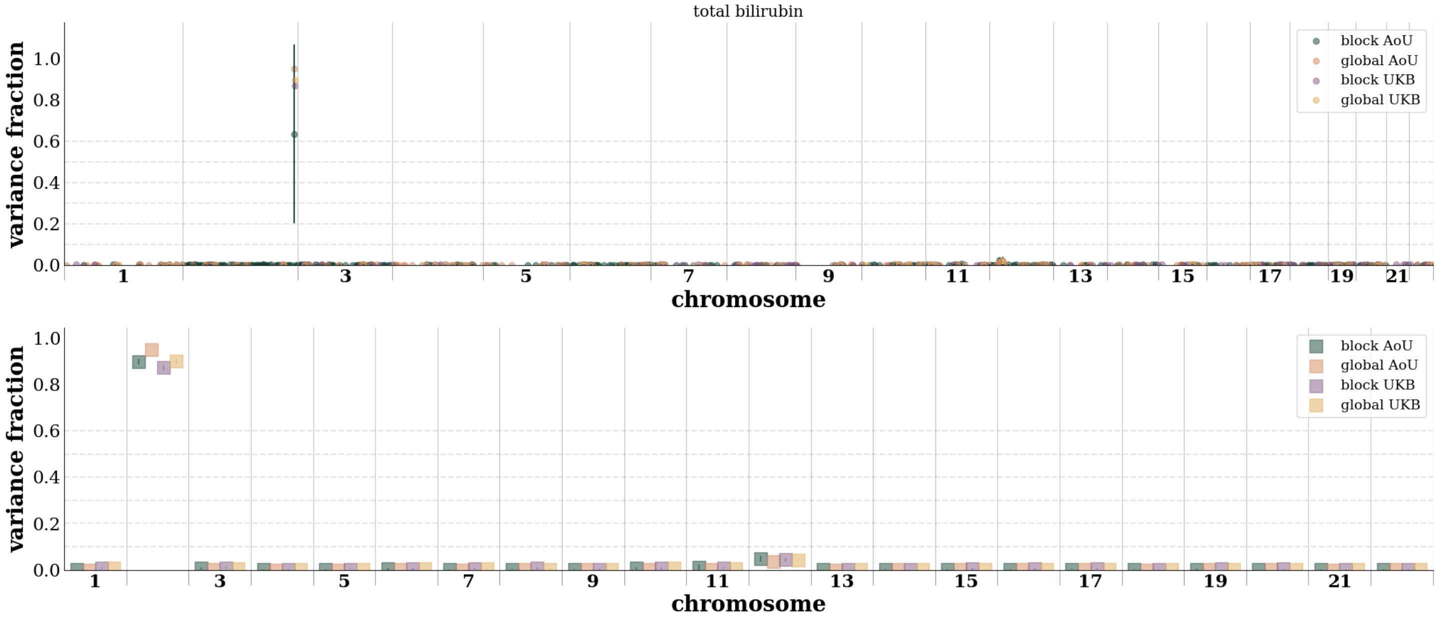

Supplemental Figure 38: Fraction of variance explained per binned base pair position (top) and per chromosome (bottom) in AoU and the UKB for total bilirubin.

6 Training and re-weighting

Here we show the additional plots which demonstrate performance as a function of training block sizes and the effect of the re-weighting the blocks: **Supplemental Figure 39 - Supplemental Figure 48**. Different training sizes – {10; 23; 50; 100; 227; 500; 1,000; 2,273; 5,000; 10,000; 22,727} SNVs – were tested in the UKB. The results for the different block sizes included the block re-weighting. All training and re-weighting was done with the EUR populations. Examples of the effects of re-weighting on all populations used from AoU can be seen in this section.

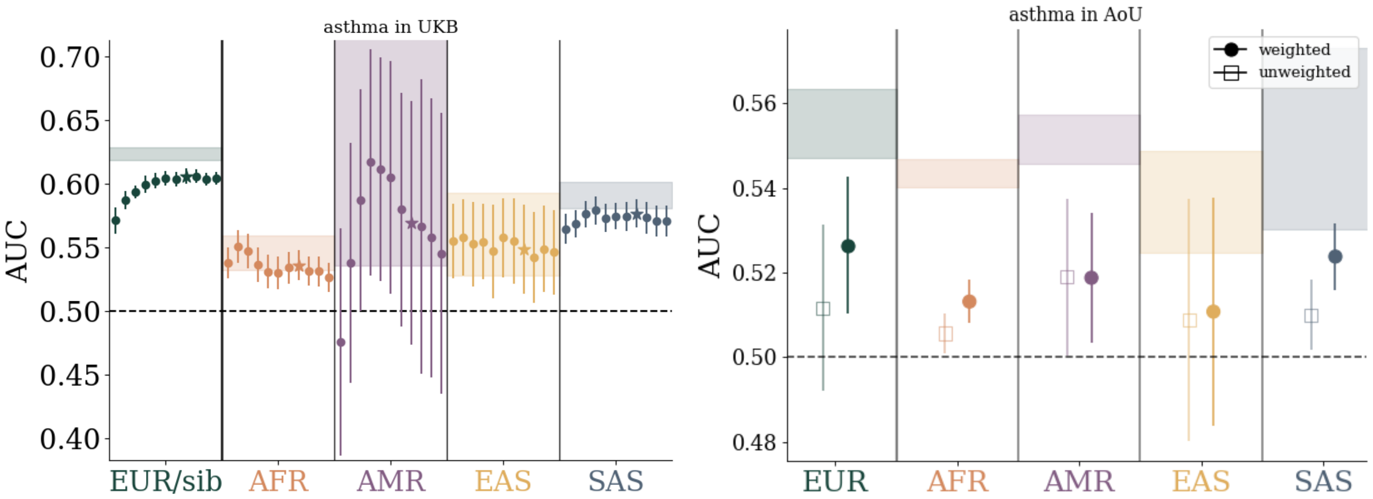

Supplemental Figure 39: Left: performance as a function of training SNV size in UKB and applied to different ancestry groups. Within each ancestry group, dots correspond to training with $\{10, 23, 50, 100, 227, 500, 1000, 2273, 5000, 10000, 22727\}$ SNVs per chromosome from left to right respectively. The starred data points correspond to 2273 SNVs per chromosome wich is roughly equivalent to 50k SNVs across the autosome. Right: performance before and after the re-weighting step of the blockLASSO. While re-weighting is trained within the EUR group, the effect of re-weighting improves prediction accuracy across all tested ancestry groupings. Colored bands indicate one standard deviation bounds for the global result. For the UKB this includes a contribution from 5 CV folds and finite sample sizes, but for AoU it only includes the finite sample contribution.

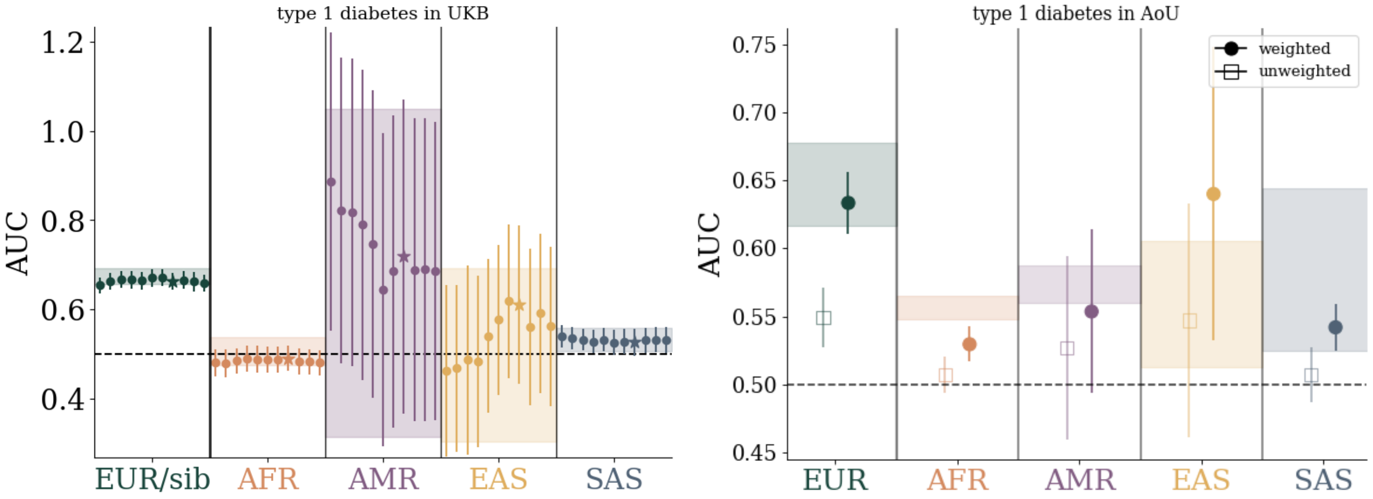

Supplemental Figure 40: Left: performance as a function of training SNV size in UKB and applied to different ancestry groups. Within each ancestry group, dots correspond to training with $\{10, 23, 50, 100, 227, 500, 1000, 2273, 5000, 10000, 22727\}$ SNVs per chromosome from left to right respectively. The starred data points correspond to 2273 SNVs per chromosome wich is roughly equivalent to 50k SNVs across the autosome. Right: performance before and after the re-weighting step of the blockLASSO. While re-weighting is trained within the EUR group, the effect of re-weighting improves prediction accuracy across all tested ancestry groupings. Colored bands indicate one standard deviation bounds for the global result. For the UKB this includes a contribution from 5 CV folds and finite sample sizes, but for AoU it only includes the finite sample contribution.

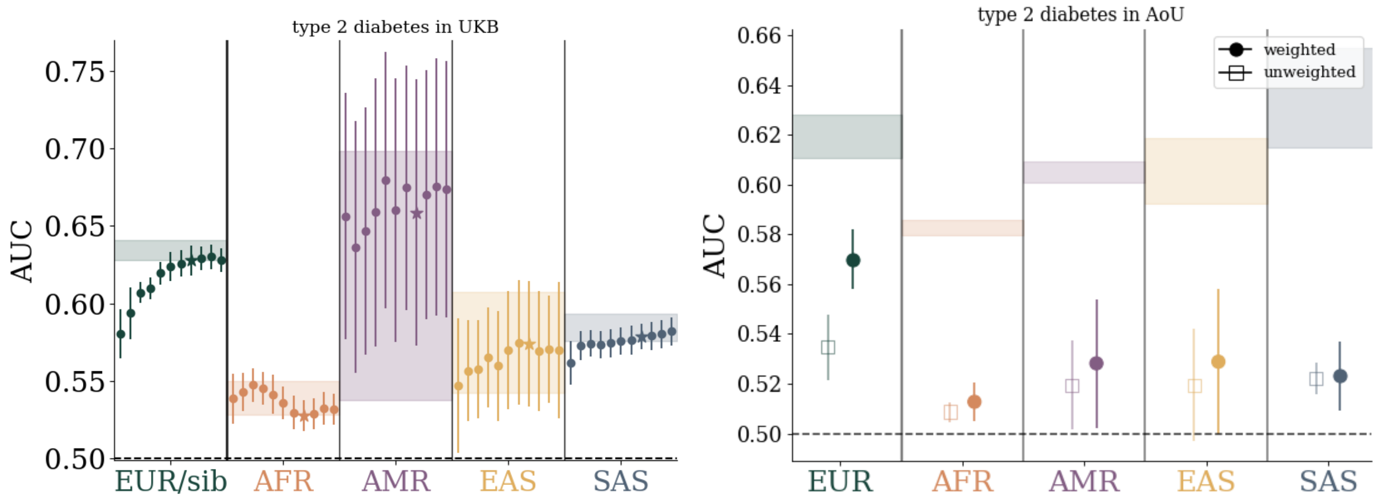

Supplemental Figure 41: Left: performance as a function of training SNV size in UKB and applied to different ancestry groups. Within each ancestry group, dots correspond to training with $\{10, 23, 50, 100, 227, 500, 1000, 2273, 5000, 10000, 22727\}$ SNVs per chromosome from left to right respectively. The starred data points correspond to 2273 SNVs per chromosome wich is roughly equivalent to 50k SNVs across the autosome. Right: performance before and after the re-weighting step of the blockLASSO. While re-weighting is trained within the EUR group, the effect of re-weighting improves prediction accuracy across all tested ancestry groupings. Colored bands indicate one standard deviation bounds for the global result. For the UKB this includes a contribution from 5 CV folds and finite sample sizes, but for AoU it only includes the finite sample contribution.

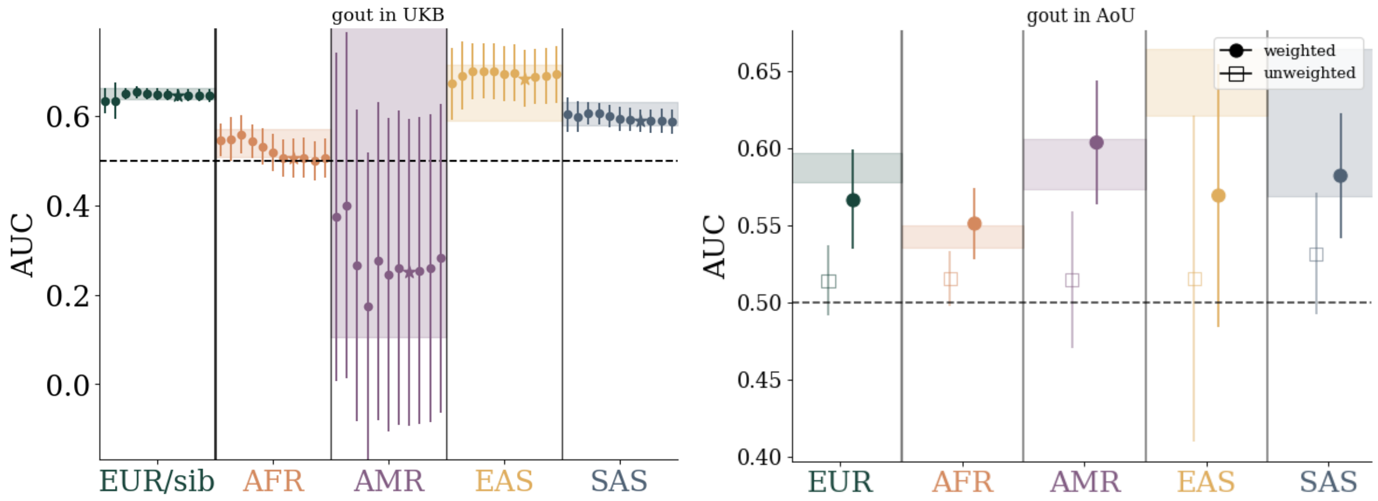

Supplemental Figure 42: Left: performance as a function of training SNV size in UKB and applied to different ancestry groups. Within each ancestry group, dots correspond to training with $\{10, 23, 50, 100, 227, 500, 1000, 2273, 5000, 10000, 22727\}$ SNVs per chromosome from left to right respectively. The starred data points correspond to 2273 SNVs per chromosome wich is roughly equivalent to 50k SNVs across the autosome. Right: performance before and after the re-weighting step of the blockLASSO. While re-weighting is trained within the EUR group, the effect of re-weighting improves prediction accuracy across all tested ancestry groupings. Colored bands indicate one standard deviation bounds for the global result. For the UKB this includes a contribution from 5 CV folds and finite sample sizes, but for AoU it only includes the finite sample contribution.

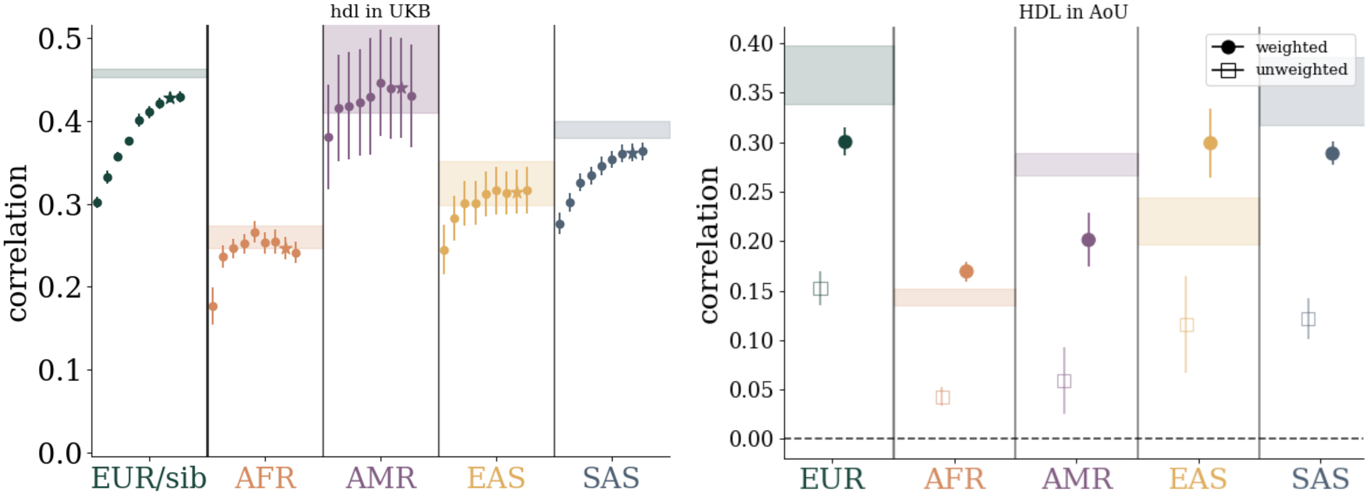

Supplemental Figure 43: Left: performance as a function of training SNV size in UKB and applied to different ancestry groups. Within each ancestry group, dots correspond to training with $\{10, 23, 50, 100, 227, 500, 1000, 2273, 5000, 10000, 22727\}$ SNVs per chromosome from left to right respectively. The starred data points correspond to 2273 SNVs per chromosome wich is roughly equivalent to 50k SNVs across the autosome. Right: performance before and after the re-weighting step of the blockLASSO. While re-weighting is trained within the EUR group, the effect of re-weighting improves prediction accuracy across all tested ancestry groupings. Colored bands indicate one standard deviation bounds for the global result. For the UKB this includes a contribution from 5 CV folds and finite sample sizes, but for AoU it only includes the finite sample contribution.

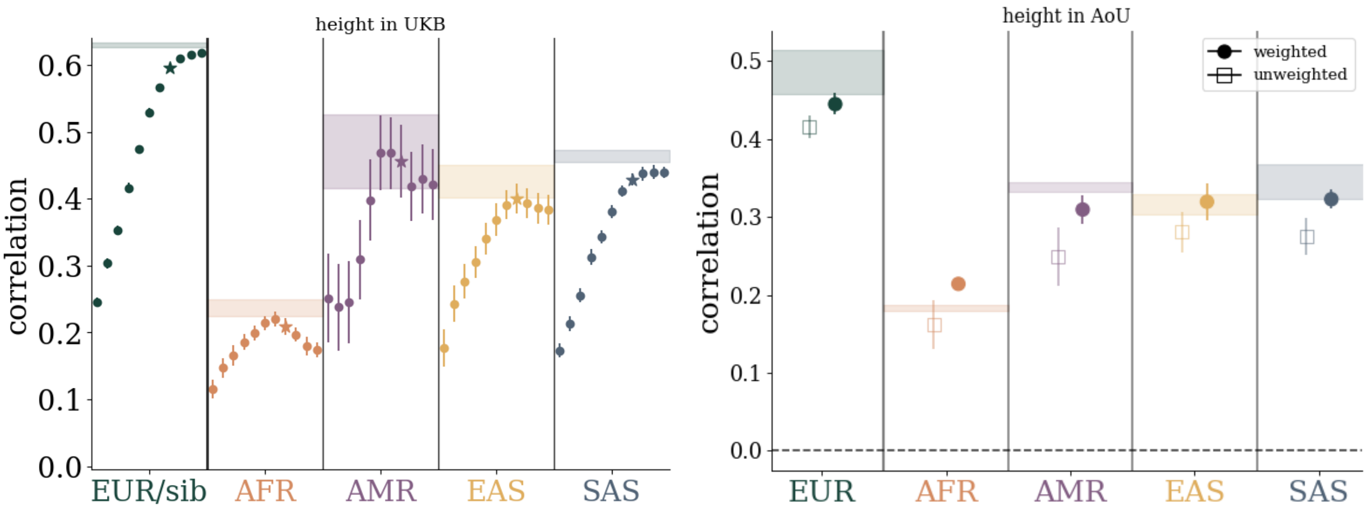

Supplemental Figure 44: Left: performance as a function of training SNV size in UKB and applied to different ancestry groups. Within each ancestry group, dots correspond to training with $\{10, 23, 50, 100, 227, 500, 1000, 2273, 5000, 10000, 22727\}$ SNVs per chromosome from left to right respectively. The starred data points correspond to 2273 SNVs per chromosome wich is roughly equivalent to 50k SNVs across the autosome. Right: performance before and after the re-weighting step of the blockLASSO. While re-weighting is trained within the EUR group, the effect of re-weighting improves prediction accuracy across all tested ancestry groupings. Colored bands indicate one standard deviation bounds for the global result. For the UKB this includes a contribution from 5 CV folds and finite sample sizes, but for AoU it only includes the finite sample contribution.

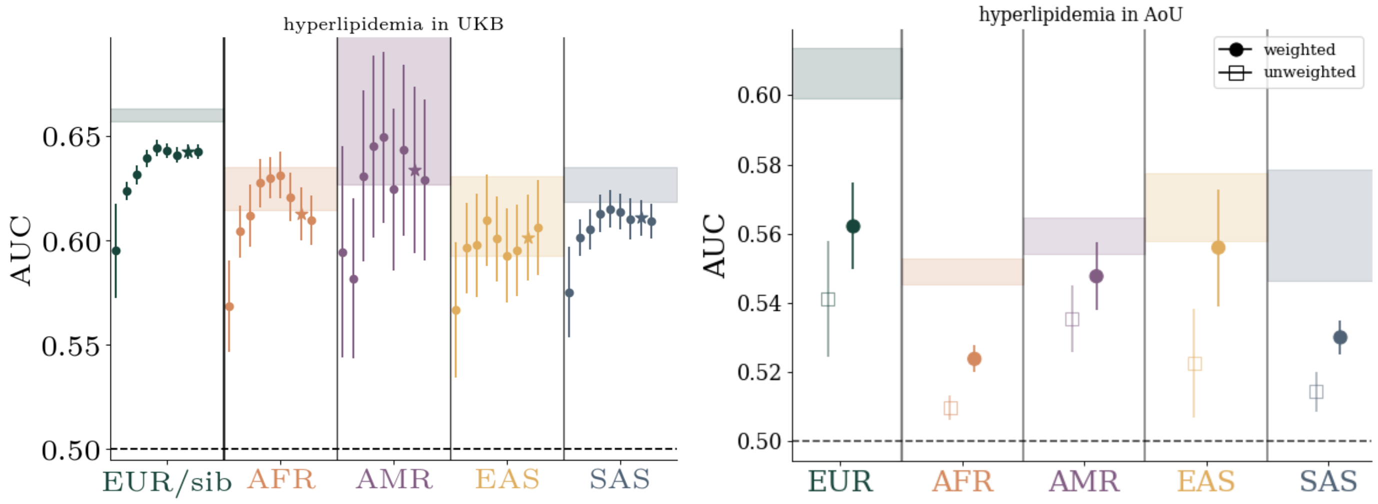

Supplemental Figure 45: Left: performance as a function of training SNV size in UKB and applied to different ancestry groups. Within each ancestry group, dots correspond to training with $\{10, 23, 50, 100, 227, 500, 1000, 2273, 5000, 10000, 22727\}$ SNVs per chromosome from left to right respectively. The starred data points correspond to 2273 SNVs per chromosome wich is roughly equivalent to 50k SNVs across the autosome. Right: performance before and after the re-weighting step of the blockLASSO. While re-weighting is trained within the EUR group, the effect of re-weighting improves prediction accuracy across all tested ancestry groupings. Colored bands indicate one standard deviation bounds for the global result. For the UKB this includes a contribution from 5 CV folds and finite sample sizes, but for AoU it only includes the finite sample contribution.

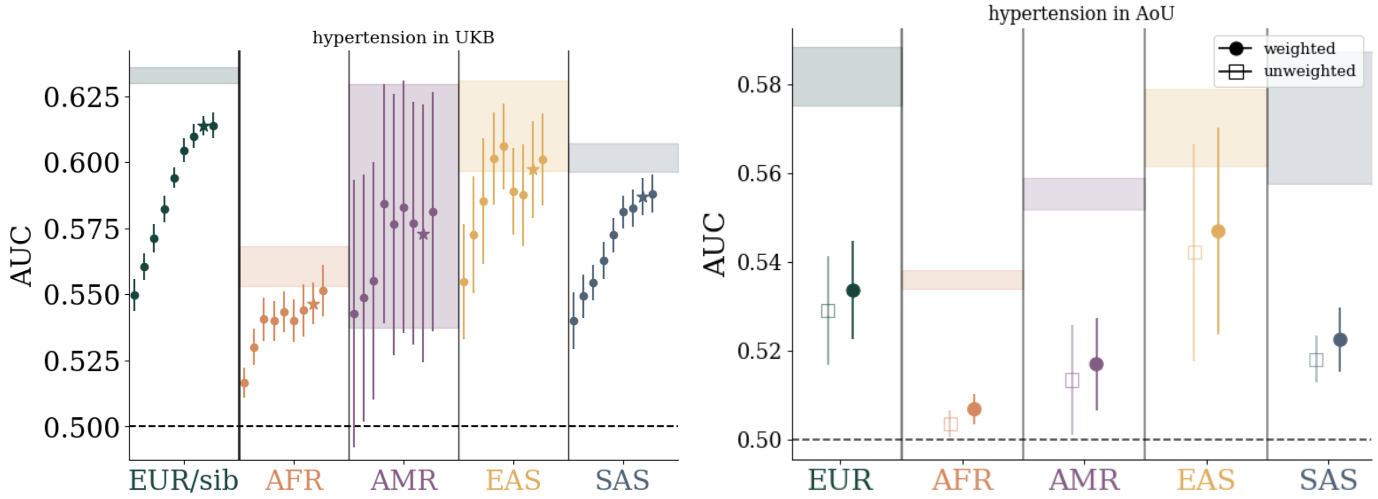

Supplemental Figure 46: Left: performance as a function of training SNV size in UKB and applied to different ancestry groups. Within each ancestry group, dots correspond to training with $\{10, 23, 50, 100, 227, 500, 1000, 2273, 5000, 10000, 22727\}$ SNVs per chromosome from left to right respectively. The starred data points correspond to 2273 SNVs per chromosome wich is roughly equivalent to 50k SNVs across the autosome. Right: performance before and after the re-weighting step of the blockLASSO. While re-weighting is trained within the EUR group, the effect of re-weighting improves prediction accuracy across all tested ancestry groupings. Colored bands indicate one standard deviation bounds for the global result. For the UKB this includes a contribution from 5 CV folds and finite sample sizes, but for AoU it only includes the finite sample contribution.

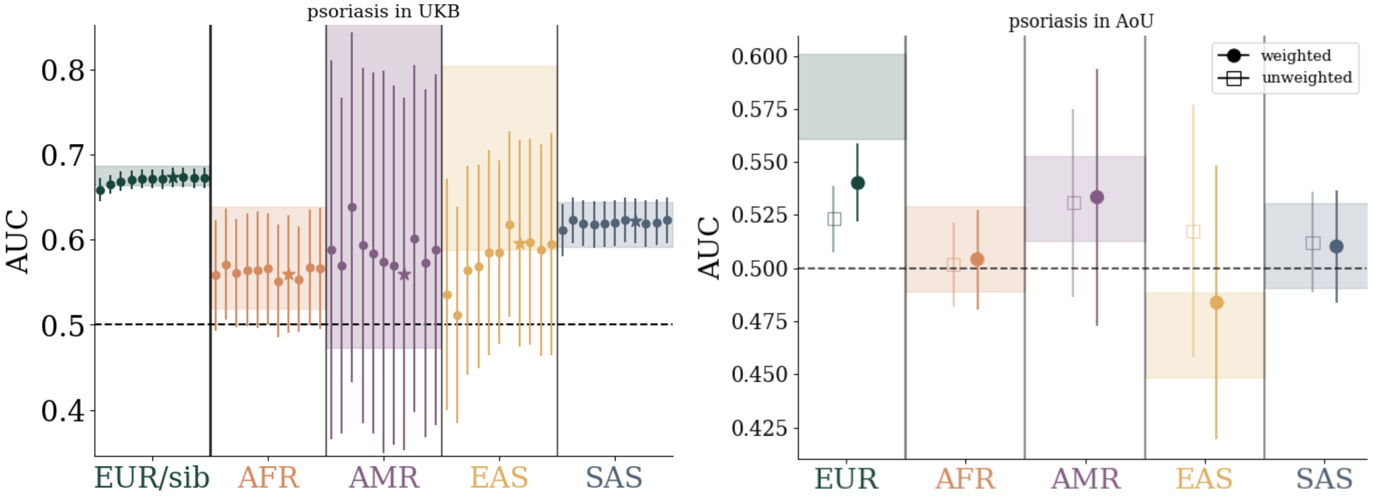

Supplemental Figure 47: Left: performance as a function of training SNV size in UKB and applied to different ancestry groups. Within each ancestry group, dots correspond to training with $\{10, 23, 50, 100, 227, 500, 1000, 2273, 5000, 10000, 22727\}$ SNVs per chromosome from left to right respectively. The starred data points correspond to 2273 SNVs per chromosome wich is roughly equivalent to 50k SNVs across the autosome. Right: performance before and after the re-weighting step of the blockLASSO. While re-weighting is trained within the EUR group, the effect of re-weighting improves prediction accuracy across all tested ancestry groupings. Colored bands indicate one standard deviation bounds for the global result. For the UKB this includes a contribution from 5 CV folds and finite sample sizes, but for AoU it only includes the finite sample contribution.

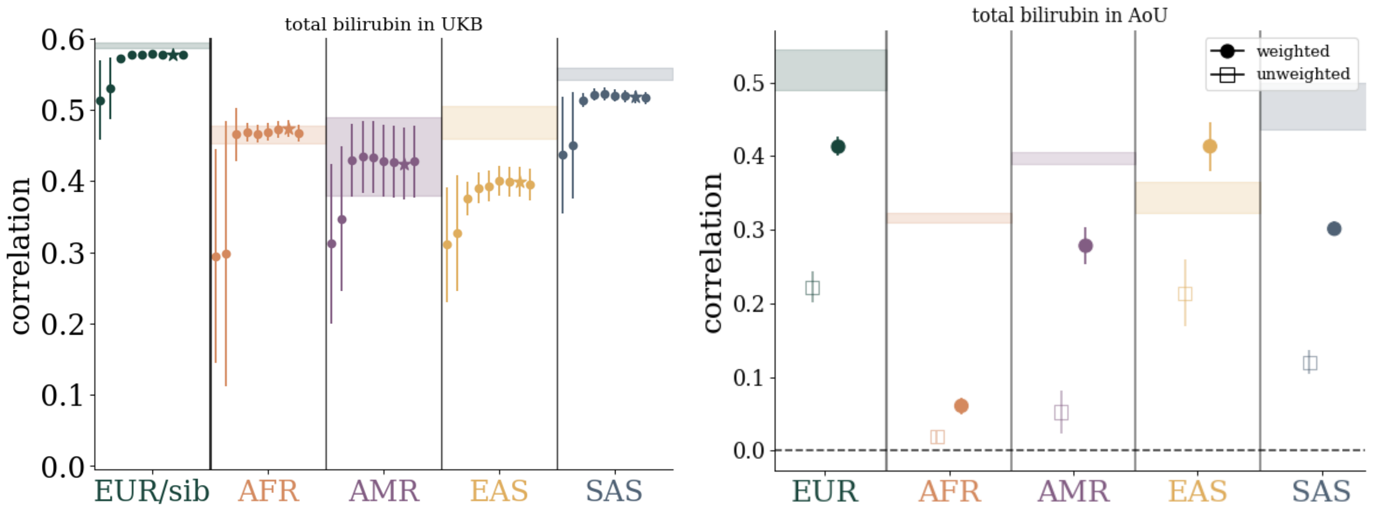

Supplemental Figure 48: Left: performance as a function of training SNV size in UKB and applied to different ancestry groups. Within each ancestry group, dots correspond to training with $\{10, 23, 50, 100, 227, 500, 1000, 2273, 5000, 10000, 22727\}$ SNVs per chromosome from left to right respectively. The starred data points correspond to 2273 SNVs per chromosome wich is roughly equivalent to 50k SNVs across the autosome. Right: performance before and after the re-weighting step of the blockLASSO. While re-weighting is trained within the EUR group, the effect of re-weighting improves prediction accuracy across all tested ancestry groupings. Colored bands indicate one standard deviation bounds for the global result. For the UKB this includes a contribution from 5 CV folds and finite sample sizes, but for AoU it only includes the finite sample contribution.

7 LASSO validation paths

Here we show, in **Supplemental Figure 49 - Supplemental Figure 59**, the LASSO training paths for the blockLASSO applied to the validation/model-selection sets. The right most column compares the combined and re-weighted blockLASSO result, shaded band, to the global LASSO path.One of the largest uses of runtime in training LASSO is in the least sparse region of the algorithm. In our plots this corresponds to the smallest region of λ or the right side of the plots. Early stopping criteria, can be used to prevent LASSO from training in this region, but as can be seen below it can be difficult to define the stopping point (i.e., when the evaluation metric peaks and starts to decrease) on blocks with a weak signal.

Additionally, we show the global LASSO training path in the UKB as applied to different ancestry groups in **Supplemental Figure 60**-**Supplemental Figure 65**. These ancestry groups are defined via self-report information. We see in these figures that the optimal hyper-parameter step can differ based on the ancestry group used and that building PGS that will work well in multiple ancestry groups can require the investigation of a large hyper-parameter space.

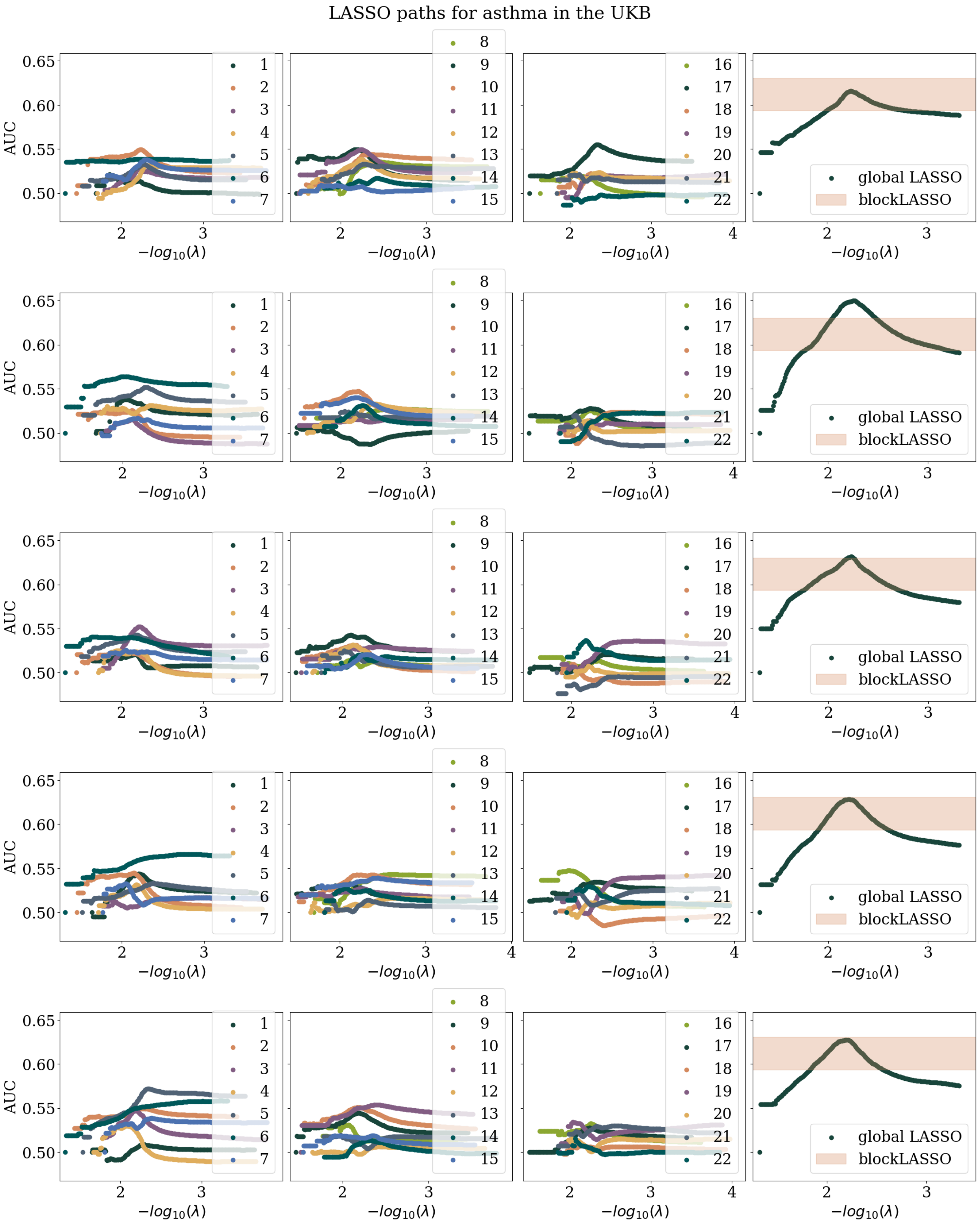

Supplemental Figure 49: Coordinate descent based LASSO paths within the validation/model-selction set within the UKB. The first three panels left to right show the paths for each chromosome for the blockLASSO construction. The fourth panel on the right shows the comparison of the global LASSO (dots) vs the complete (i.e., re-weighted) blockLASSO value in the validation set. Different rows correspond to different cross-validation folds.

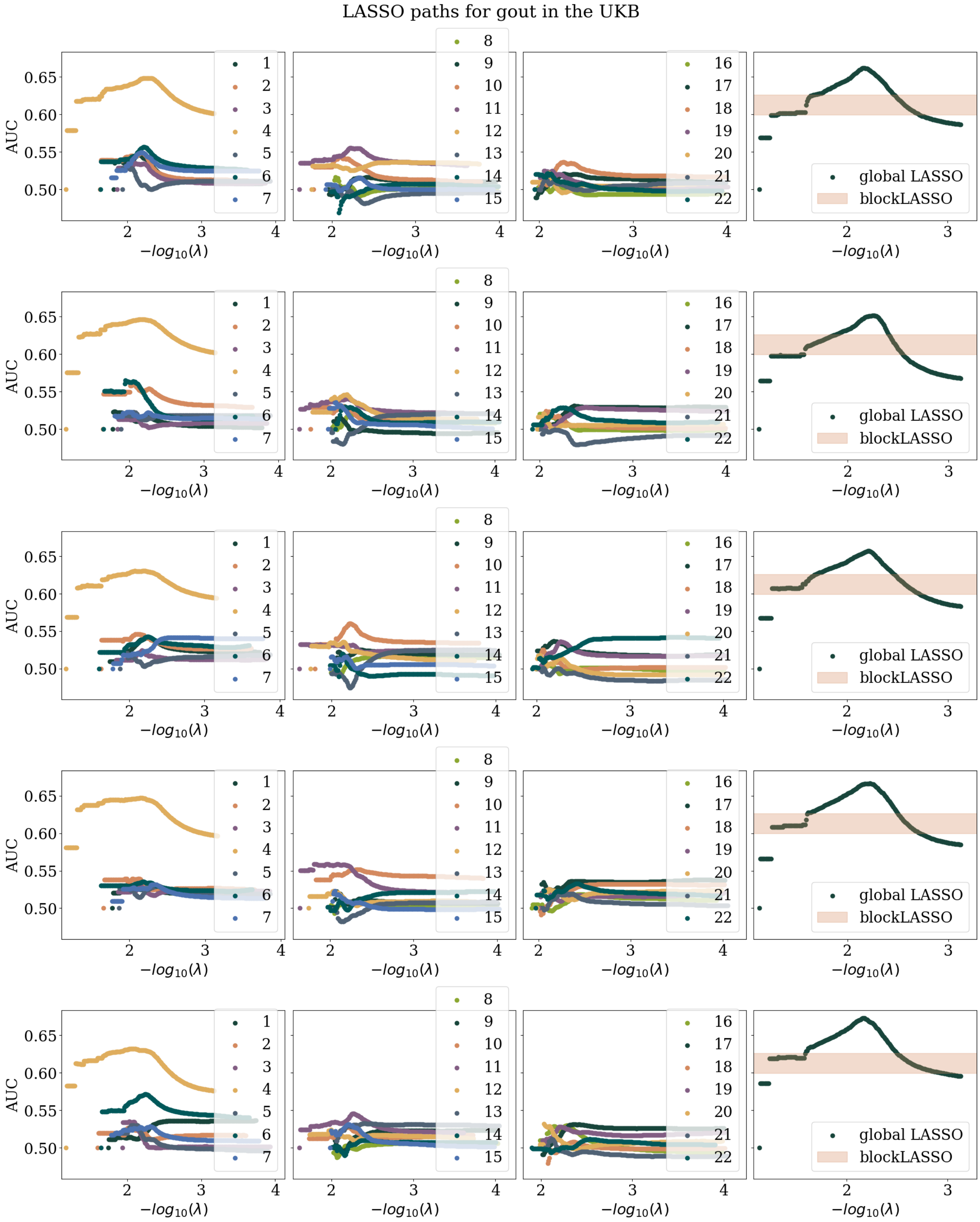

Supplemental Figure 50: Coordinate descent based LASSO paths within the validation/model-selction set within the UKB. The first three panels left to right show the paths for each chromosome for the blockLASSO construction. The fourth panel on the right shows the comparison of the global LASSO (dots) vs the complete (i.e., re-weighted) blockLASSO value in the validation set. Different rows correspond to different cross-validation folds.

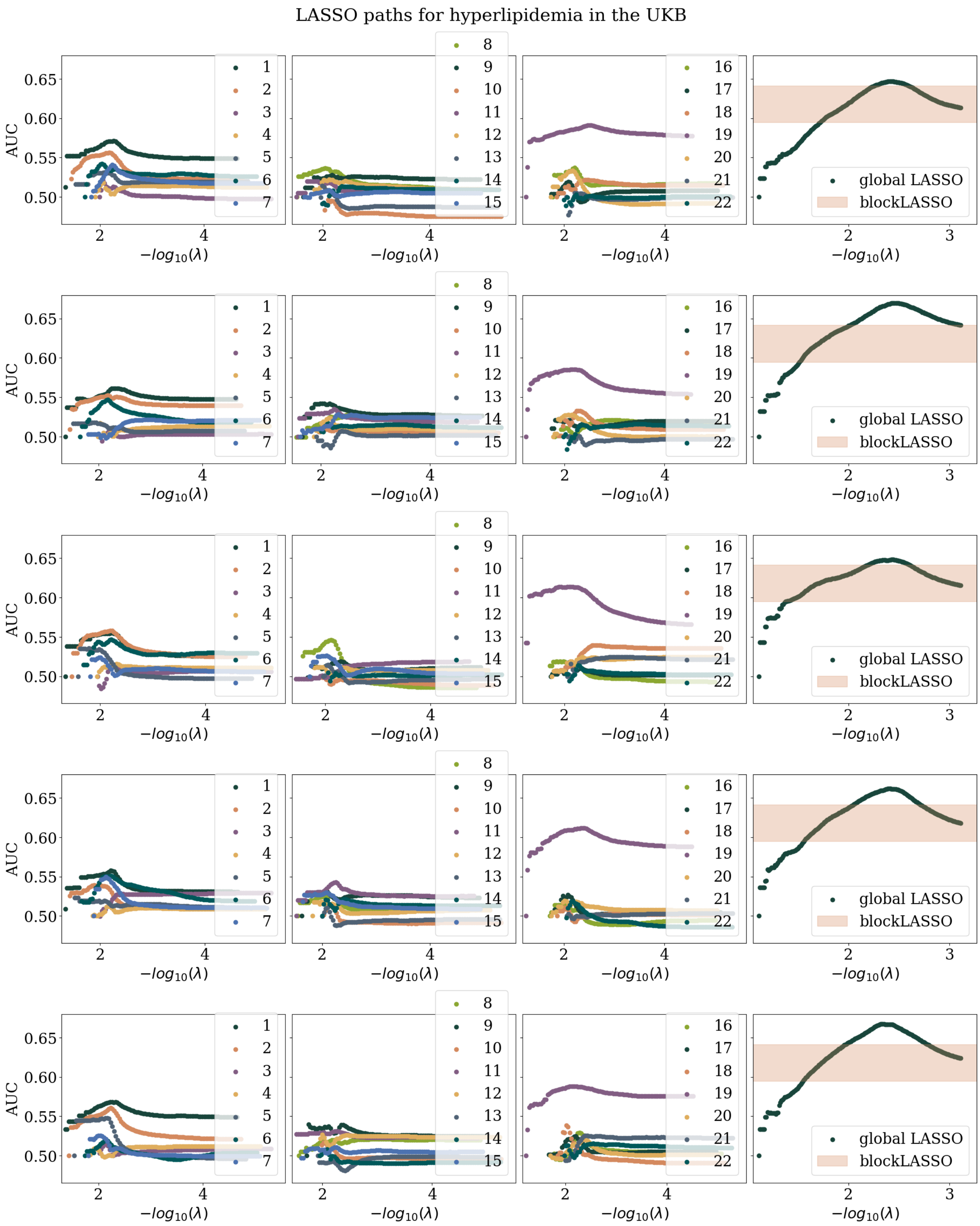

Supplemental Figure 51: Coordinate descent based LASSO paths within the validation/model-selction set within the UKB. The first three panels left to right show the paths for each chromosome for the blockLASSO construction. The fourth panel on the right shows the comparison of the global LASSO (dots) vs the complete (i.e., re-weighted) blockLASSO value in the validation set. Different rows correspond to different cross-validation folds.

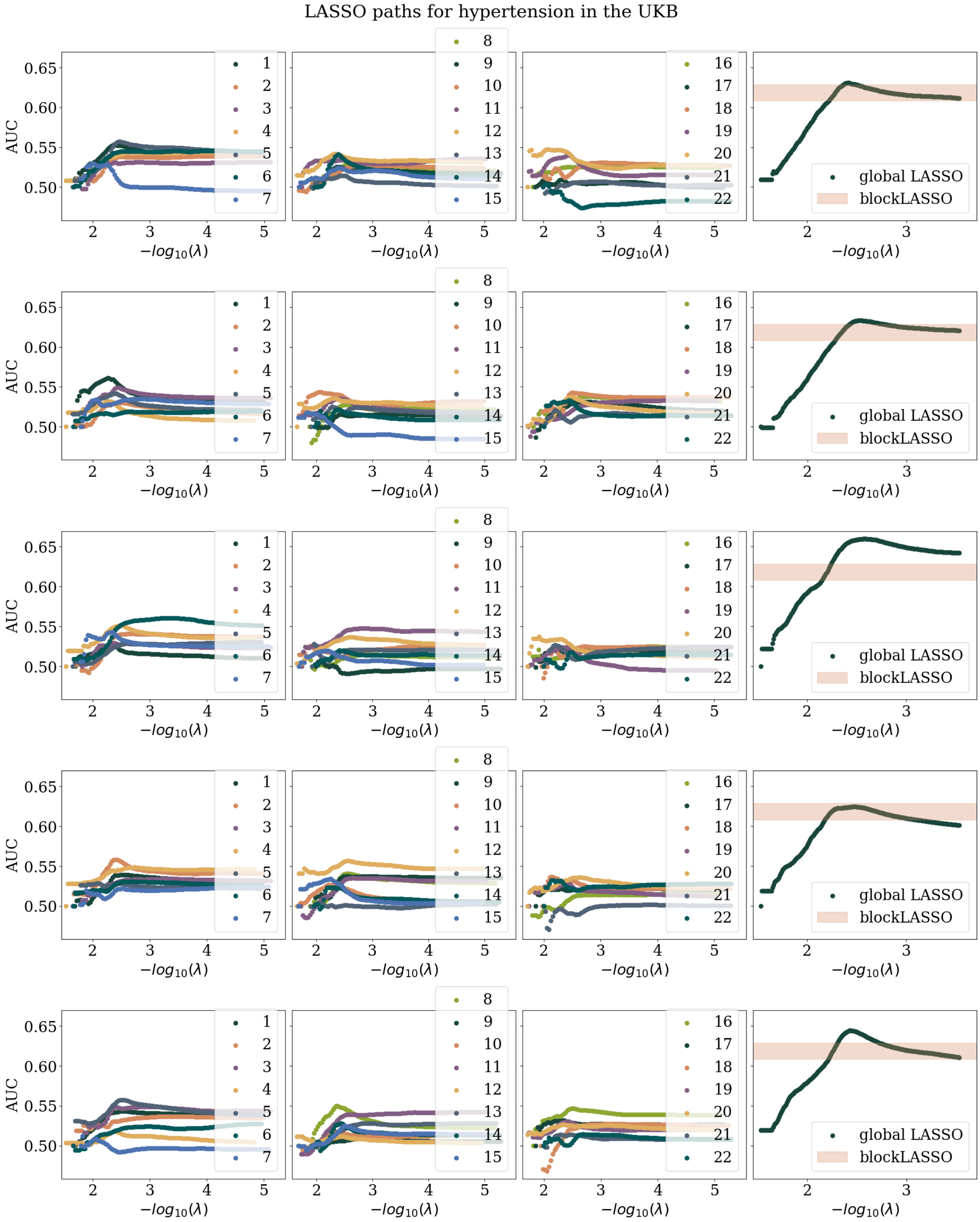

Supplemental Figure 52: Coordinate descent based LASSO paths within the validation/model-selction set within the UKB. The first three panels left to right show the paths for each chromosome for the blockLASSO construction. The fourth panel on the right shows the comparison of the global LASSO (dots) vs the complete (i.e., re-weighted) blockLASSO value in the validation set. Different rows correspond to different cross-validation folds.

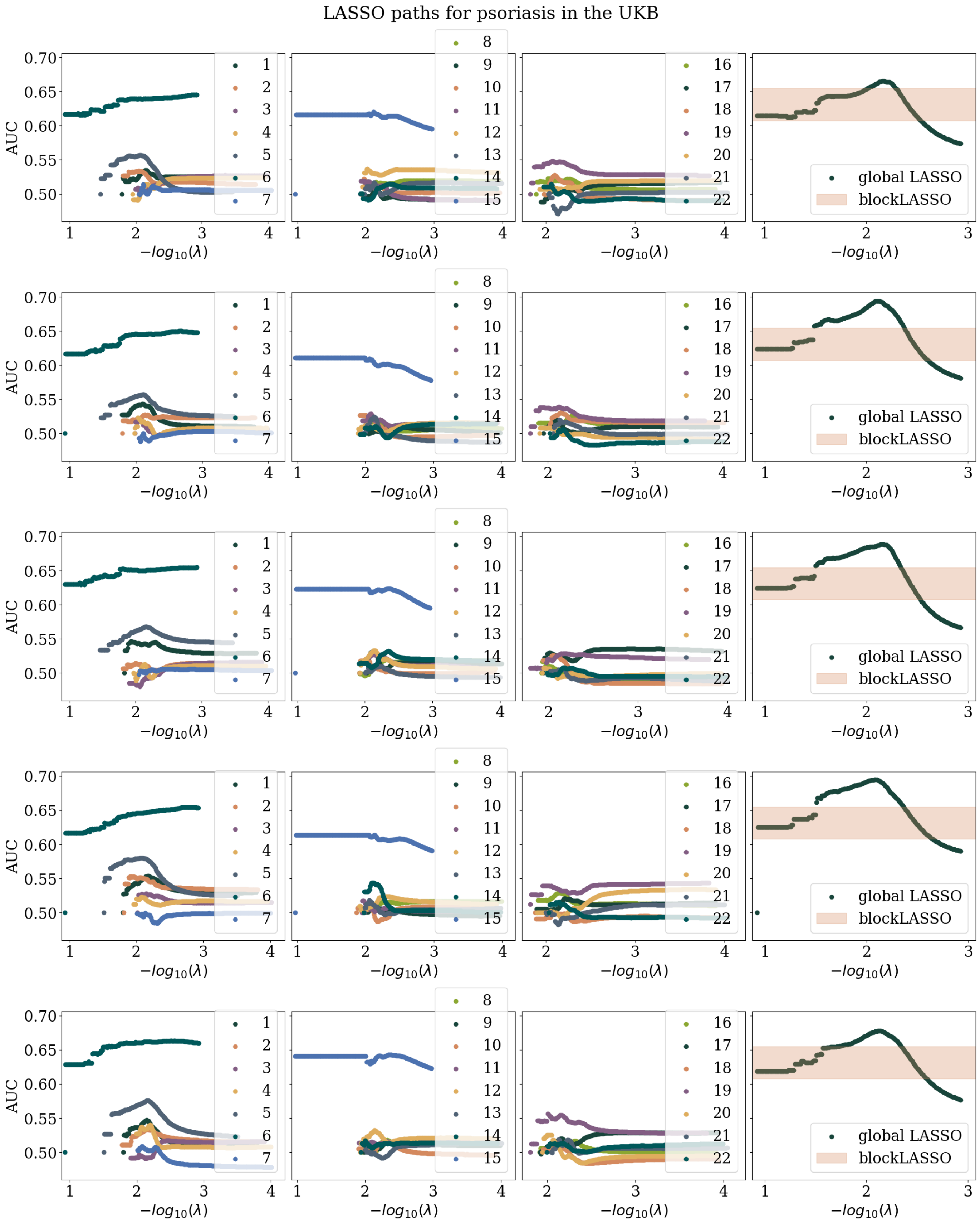

Supplemental Figure 53: Coordinate descent based LASSO paths within the validation/model-selction set within the UKB. The first three panels left to right show the paths for each chromosome for the blockLASSO construction. The fourth panel on the right shows the comparison of the global LASSO (dots) vs the complete (i.e., re-weighted) blockLASSO value in the validation set. Different rows correspond to different cross-validation folds.

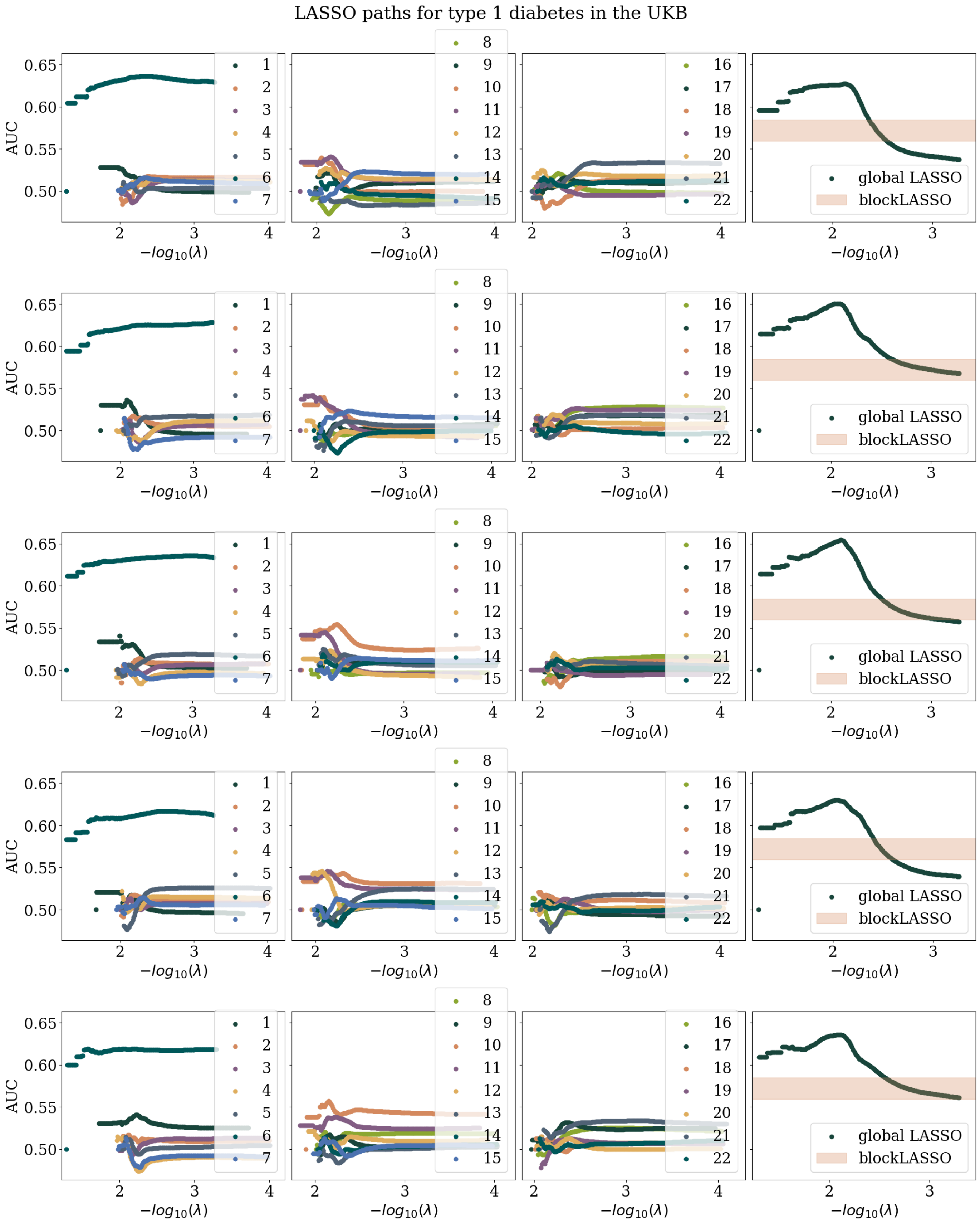

Supplemental Figure 54: Coordinate descent based LASSO paths within the validation/model-selction set within the UKB. The first three panels left to right show the paths for each chromosome for the blockLASSO construction. The fourth panel on the right shows the comparison of the global LASSO (dots) vs the complete (i.e., re-weighted) blockLASSO value in the validation set. Different rows correspond to different cross-validation folds.

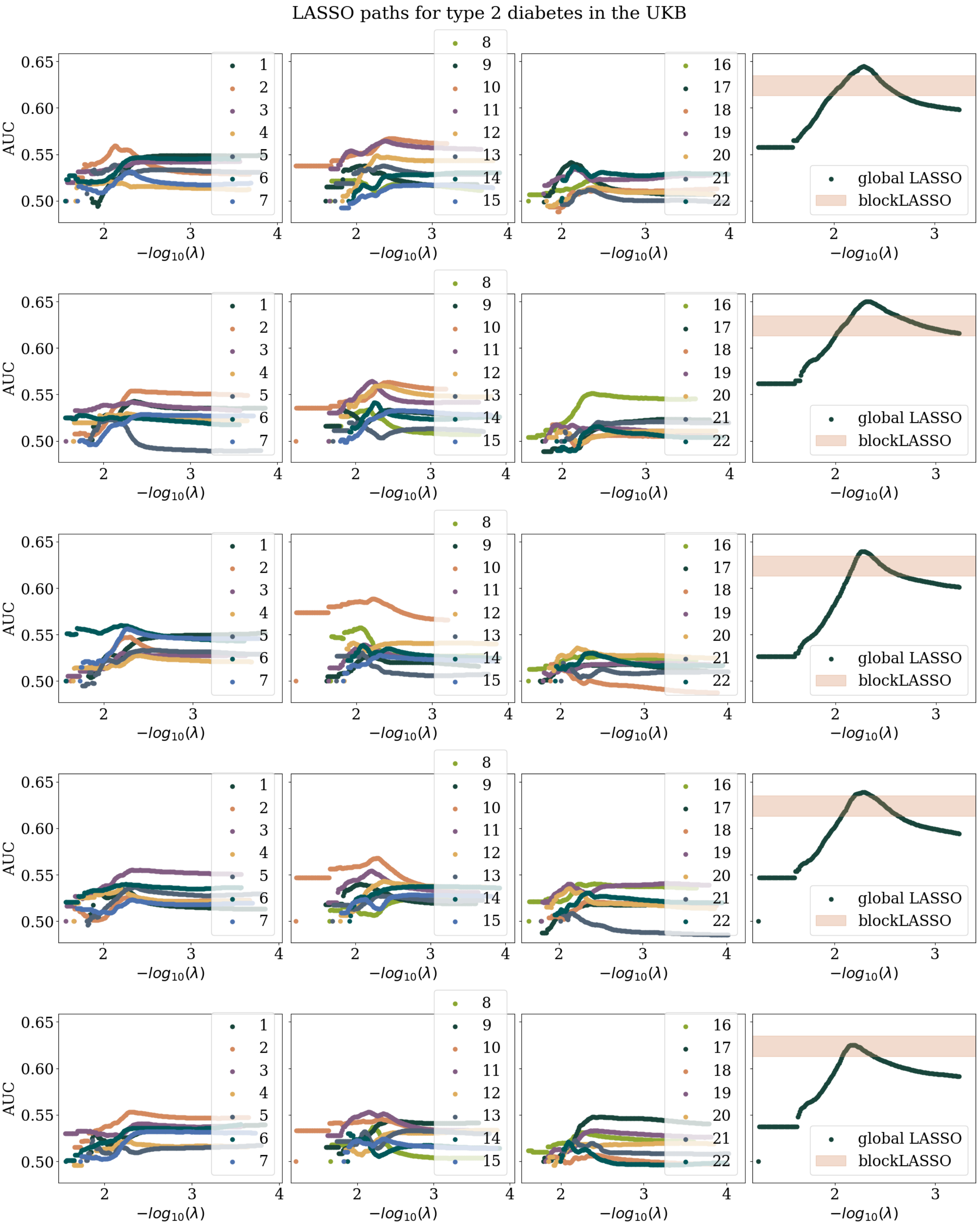

Supplemental Figure 55: Coordinate descent based LASSO paths within the validation/model-selction set within the UKB. The first three panels left to right show the paths for each chromosome for the blockLASSO construction. The fourth panel on the right shows the comparison of the global LASSO (dots) vs the complete (i.e., re-weighted) blockLASSO value in the validation set. Different rows correspond to different cross-validation folds.

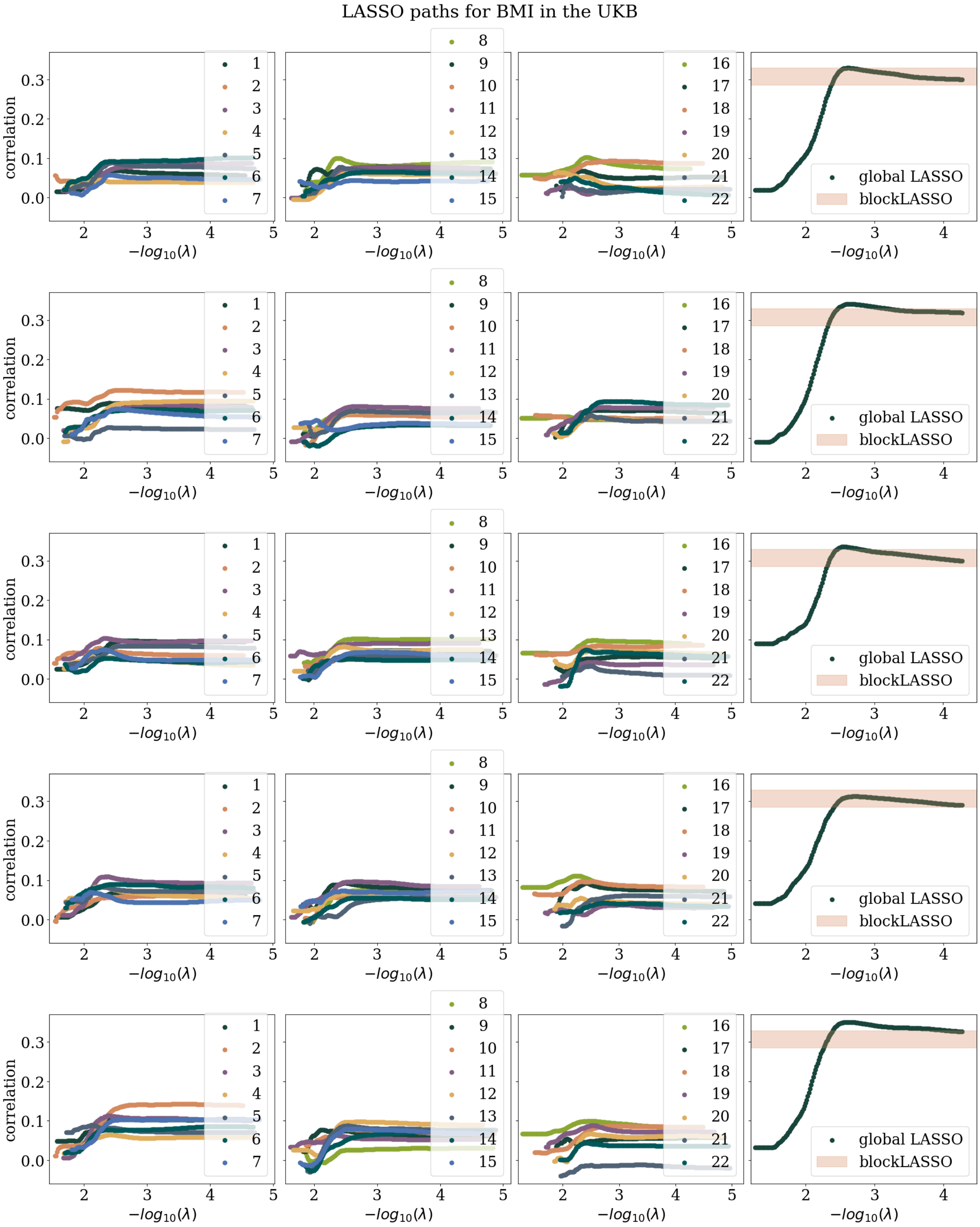

Supplemental Figure 56: Coordinate descent based LASSO paths within the validation/model-selction set within the UKB. The first three panels left to right show the paths for each chromosome for the blockLASSO construction. The fourth panel on the right shows the comparison of the global LASSO (dots) vs the complete (i.e., re-weighted) blockLASSO value in the validation set. Different rows correspond to different cross-validation folds.

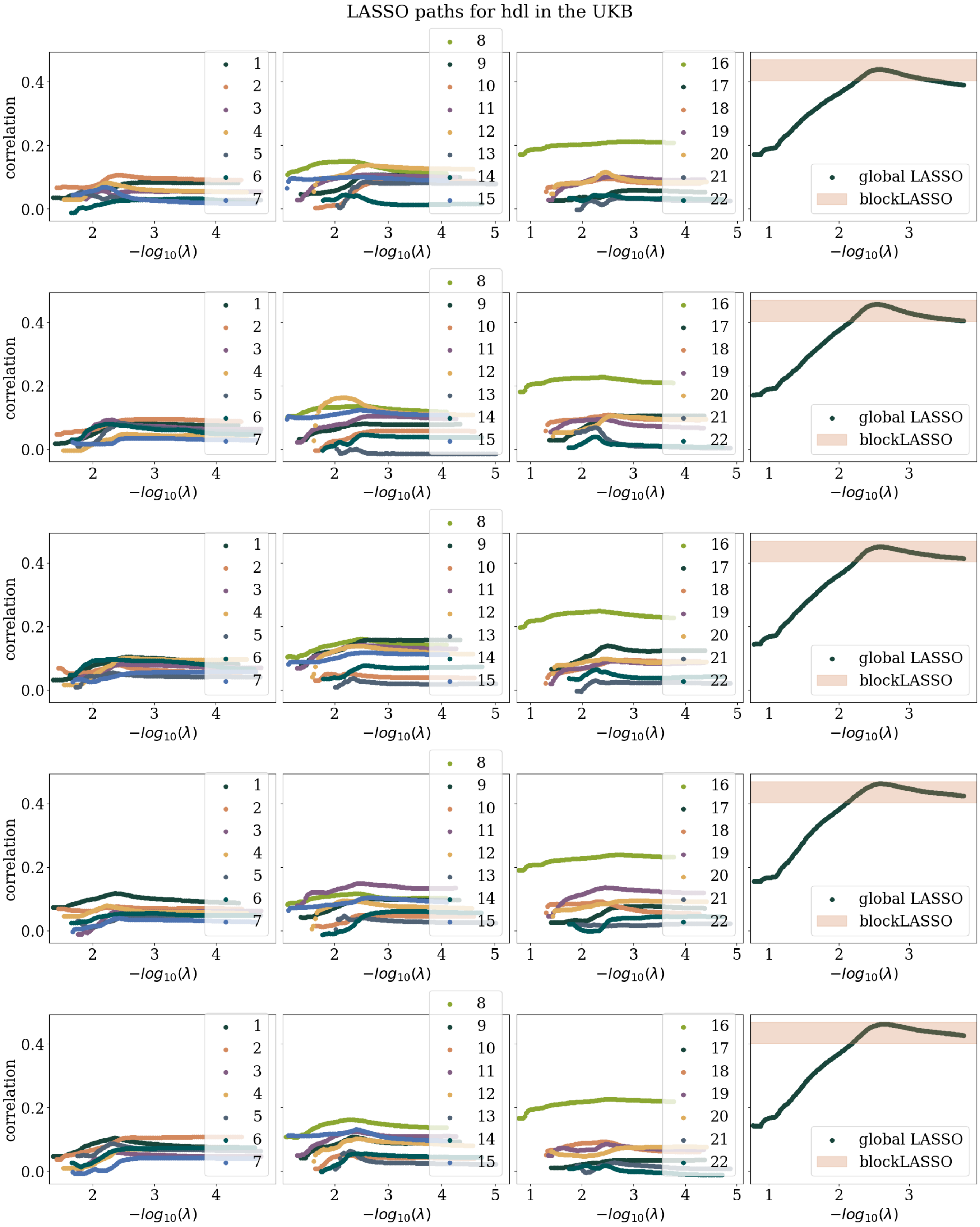

Supplemental Figure 57: Coordinate descent based LASSO paths within the validation/model-selction set within the UKB. The first three panels left to right show the paths for each chromosome for the blockLASSO construction. The fourth panel on the right shows the comparison of the global LASSO (dots) vs the complete (i.e., re-weighted) blockLASSO value in the validation set. Different rows correspond to different cross-validation folds.

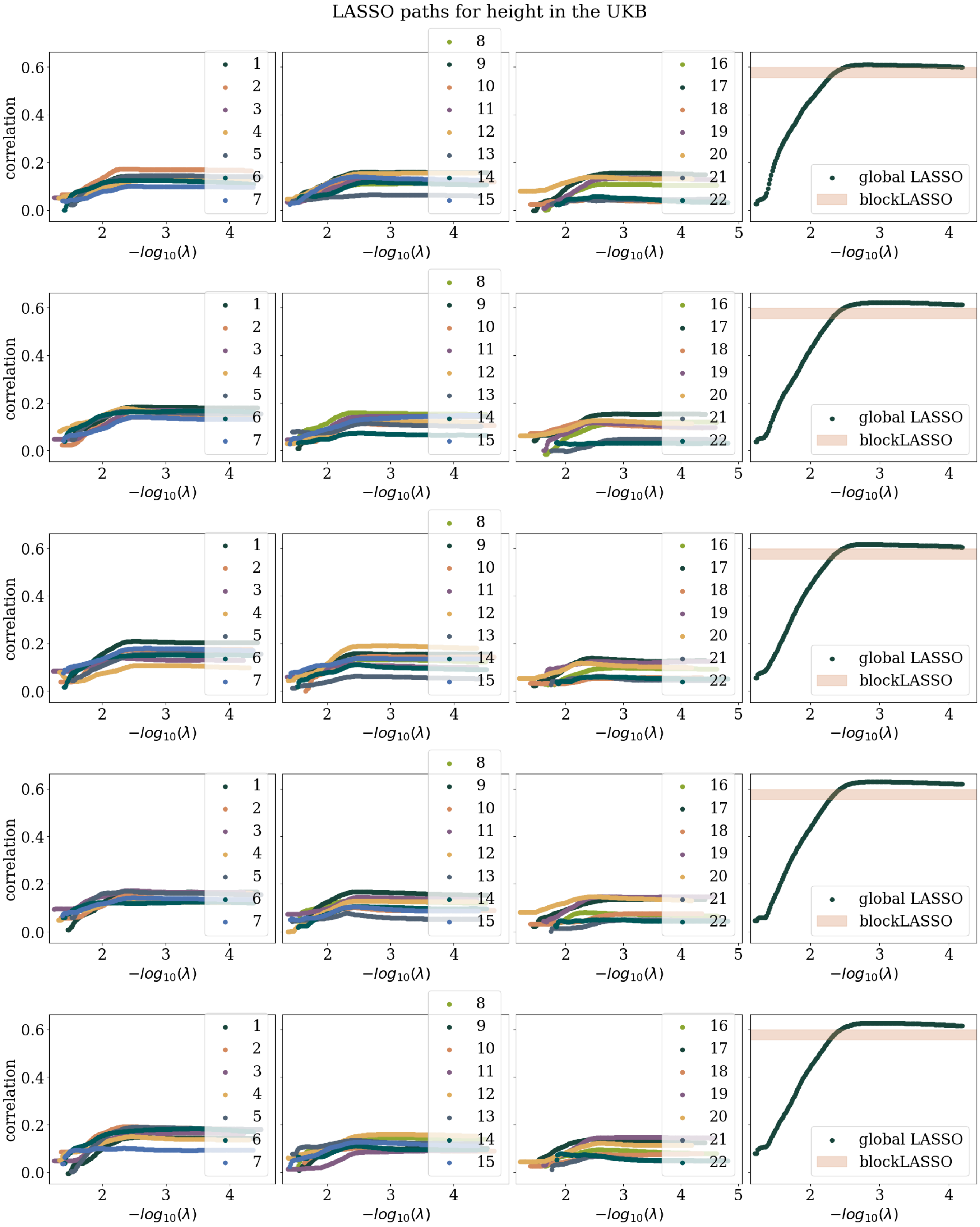

Supplemental Figure 58: Coordinate descent based LASSO paths within the validation/model-selction set within the UKB. The first three panels left to right show the paths for each chromosome for the blockLASSO construction. The fourth panel on the right shows the comparison of the global LASSO (dots) vs the complete (i.e., re-weighted) blockLASSO value in the validation set. Different rows correspond to different cross-validation folds.

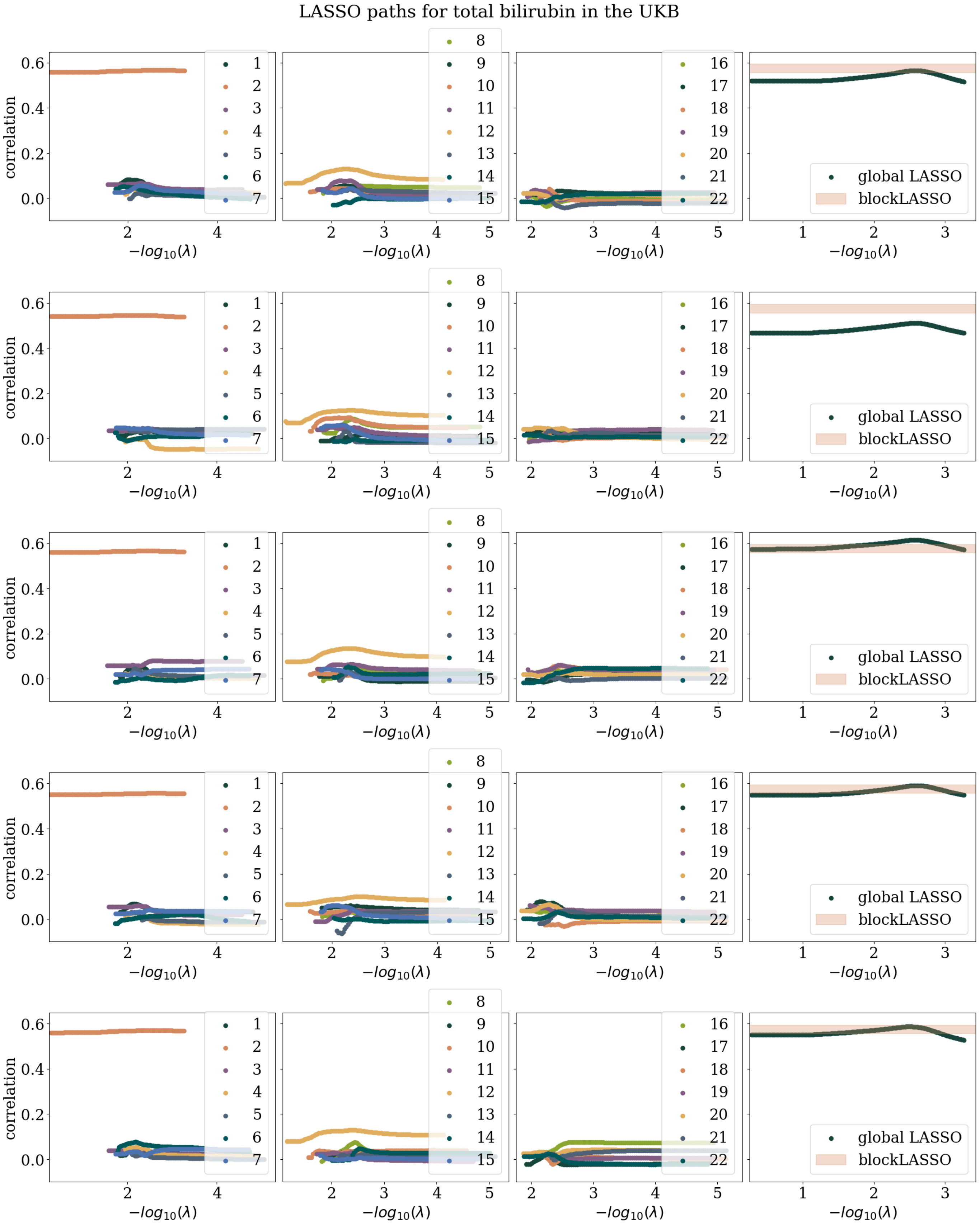

Supplemental Figure 59: Coordinate descent based LASSO paths within the validation/model-selction set within the UKB. The first three panels left to right show the paths for each chromosome for the blockLASSO construction. The fourth panel on the right shows the comparison of the global LASSO (dots) vs the complete (i.e., re-weighted) blockLASSO value in the validation set. Different rows correspond to different cross-validation folds.

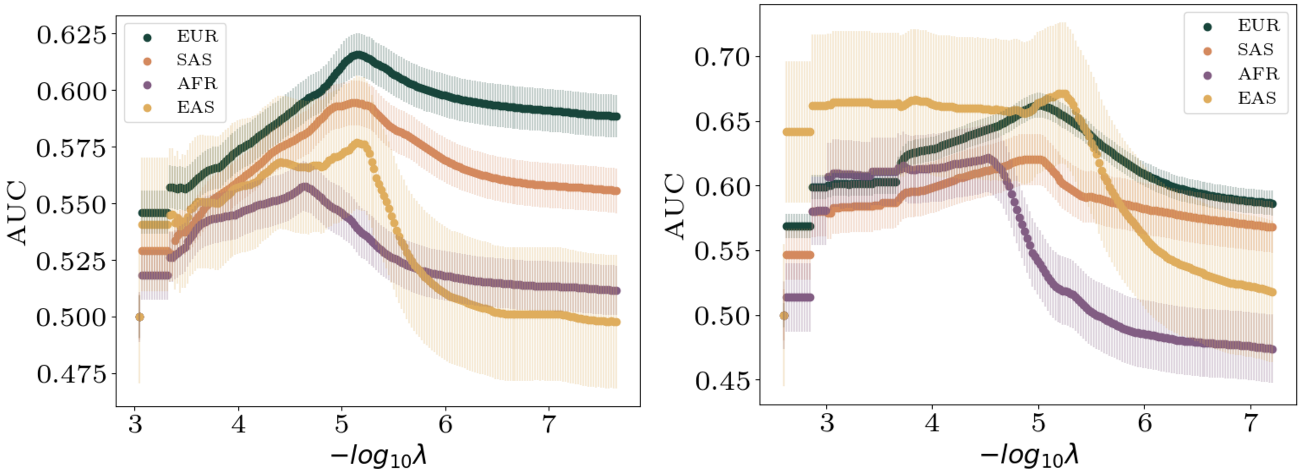

Supplemental Figure 60: Validation paths for UKB PGS trained on a European population but then applied to other ancestry groups for asthma (left) and gout (right).

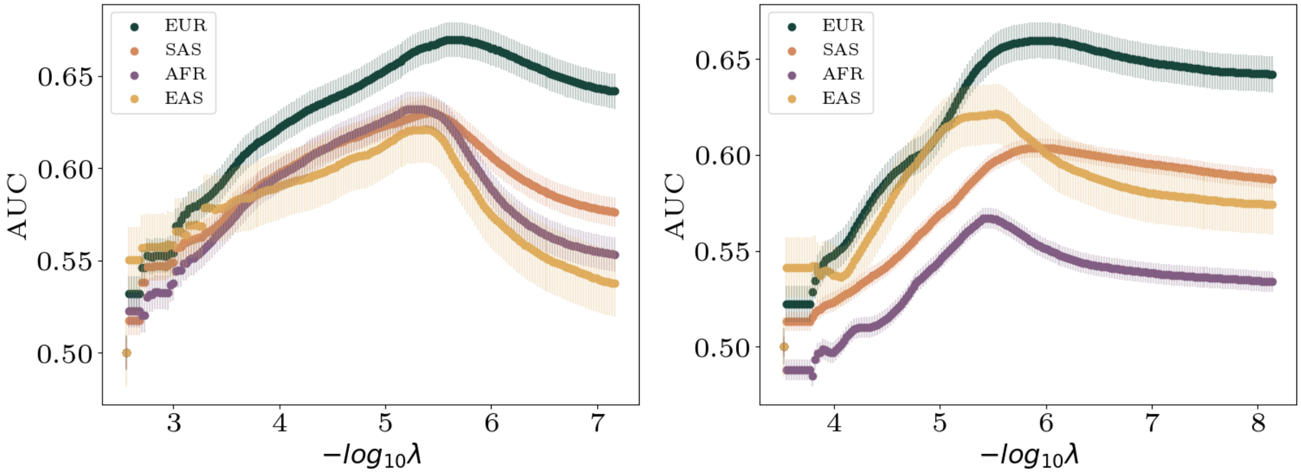

Supplemental Figure 61: Validation paths for UKB PGS trained on a European population but then applied to other ancestry groups for hyperlipidemia (left) and hypertension (right).

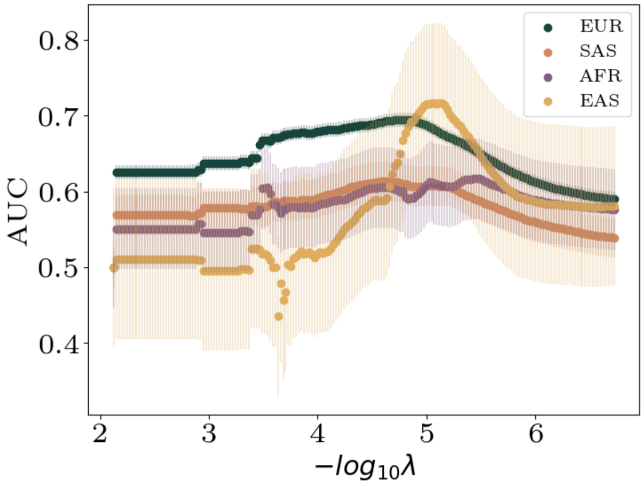

Supplemental Figure 62: Validation paths for UKB PGS trained on a European population but then applied to other ancestry groups for psoriasis.

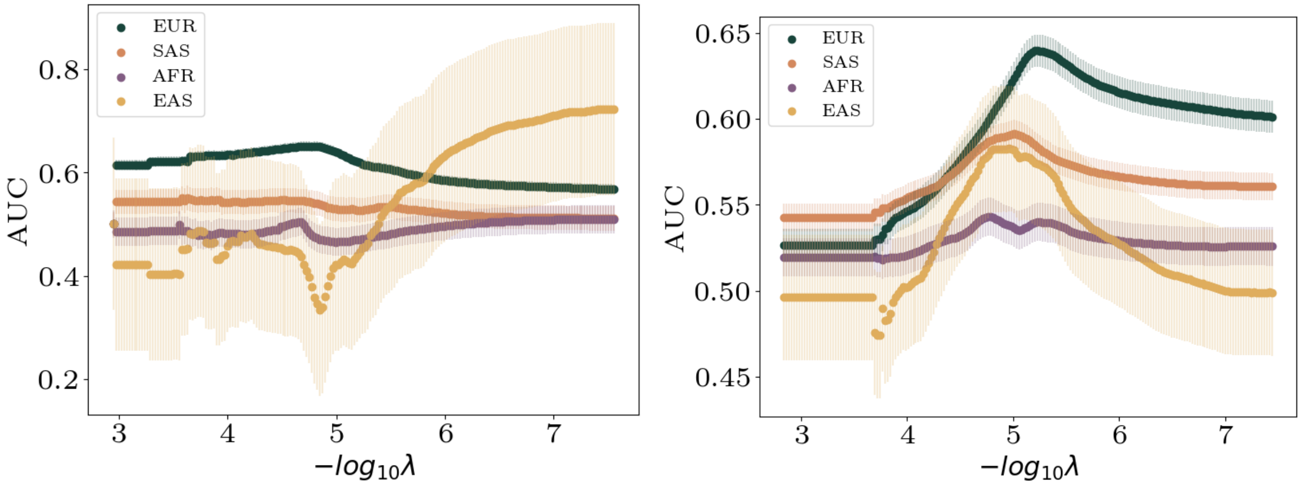

Supplemental Figure 63: Validation paths for UKB PGS trained on a European population but then applied to other ancestry groups for type 1 diabetes (left) and type 2 diabetes (right).

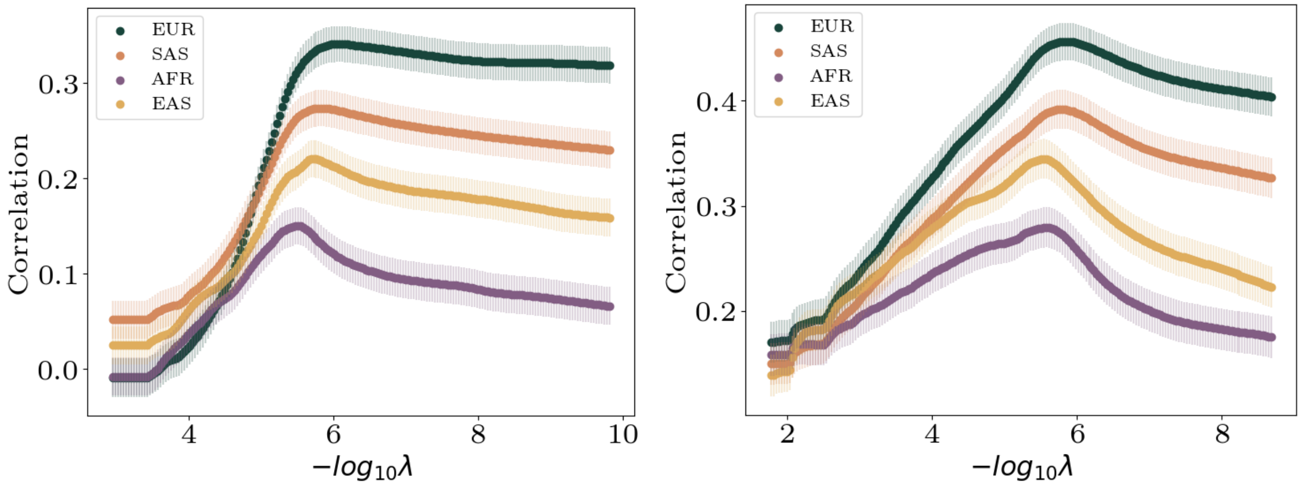

Supplemental Figure 64: Validation paths for UKB PGS trained on a European population but then applied to other ancestry groups for BMI (left) and HDL (right).

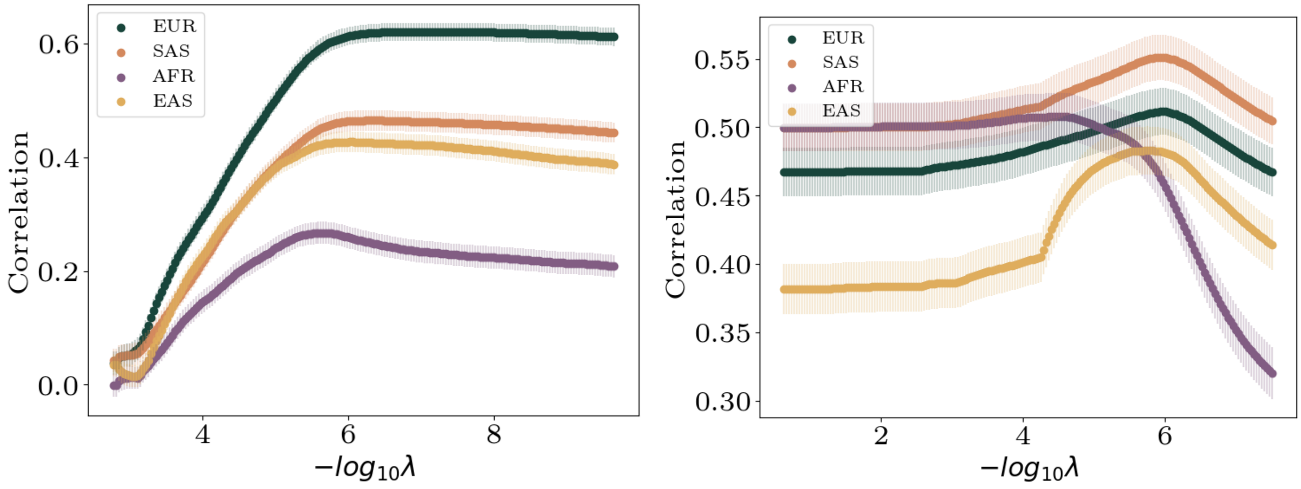

Supplemental Figure 65: Validation paths for UKB PGS trained on a European population but then applied to other ancestry groups forheight (left) and total bilirubin (right).

References

1. Tuyeras, R. *HaploDynamics: A python library to develop genomic data simulators* `http://web.archive.org/web/20080207010024/http://www.808multimedia.com/winnt/kernel.htm`. (Version 0.4-beta.1) [Computer software]. 2023 (cit. on p. 19).

2. Privé, F., Aschard, H. & Blum, M. Efficient Implementation of Penalized Regression for Genetic Risk Prediction. *Genetics* **212,** 65–74 (2019) (cit. on p. 19).

3. Privé, F. *et al.* High-resolution portability of 245 polygenic scores when derived and applied in the same cohort. *medRxiv* (2021) (cit. on p. 19).
